# Uncovering the signaling networks of disseminated glioblastoma cells in vivo with INSIGHT

**DOI:** 10.1038/s41467-026-76587-0

**Published:** 2026-08-08

**Authors:** Ryuhjin Ahn, Alicia D. D’Souza, Lawrence Long, Yufei Cui, Jennifer Gantchev, Gerard Baquer, Danielle Burgenske, Katrina K. Bakken, Lauren L. Ott, Brett L. Carlson, Grace Zhou, Ishwar N. Kohale, Charles A. Whittaker, Heidi Temple, Tomer M. Yaron-Barir, Jeffrey Wyckoff, Terry C. Burns, Rachael A. Vaubel, Cameron T. Flower, Wei Huang, Ann Tuma, Jared L. Johnson, Nathalie Y. R. Agar, Josie Ursini-Siegel, Jann N. Sarkaria, Forest M. White

**Affiliations:** 1 David H. Koch Institute for Integrative Cancer Research, Massachusetts Institute of Technology (MIT), Cambridge, MA USA; 2 Department of Biological Engineering, MIT, Cambridge, MA USA; 3 MIT-Harvard Health Sciences and Technology, Cambridge, MA USA; 4 Department of Neurosurgery, Brigham and Women’s Hospital, Harvard Medical School, Boston, MA USA; 5 Department of Electronic Engineering, Rovira i Virgili University, Tarragona, Spain; 6 Mayo Clinic, Rochester, MN USA; 7 MIT, Cambridge, MA USA; 8 Barbara K. Ostrom (1978) Bioinformatics and Computing Core Facility of the Swanson Biotechnology Center, David H. Koch Institute for Integrative Cancer Research, Cambridge, USA; 9 Columbia University Vagelos College of Physicians and Surgeons, New York, NY USA; 10 Microscopy Core Facility of the Swanson Biotechnology Center, David H. Koch Institute for Integrative Cancer Research, Cambridge, USA; 11 Program in Computational and Systems Biology, MIT, Cambridge, MA USA; 12 Preclinical Imaging and Testing Facility of the Swanson Biotechnology Center, David H. Koch Institute for Integrative Cancer Research, MIT, Cambridge, MA USA; 13 Department of Cell Biology, Harvard Medical School, Boston, MA USA; 14 Dana-Farber Cancer Institute, Harvard Medical School, Boston, MA USA; 15 Department of Radiology, Brigham and Women’s Hospital, Harvard Medical School, Boston, MA USA; 16 Department of Cancer Biology, Dana-Farber Cancer Institute, Boston, MA USA; 17 Division of Experimental Medicine, McGill University, Montreal, QC Canada; 18 Lady Davis Institute for Medical Research, Jewish General Hospital, Montreal, QC Canada

**Keywords:** Metastasis, Cancer microenvironment

## Abstract

Dysregulation of intracellular signaling networks underpins cancer. Yet, resolving signaling networks within distinct or rare cell types in cancer in vivo has been unattainable. Here we develop INSIGHT by integrating cell sorting with mass spectrometry to enable quantitative phosphoproteomics and proteomics of discrete cell types from fixed tissues. Using INSIGHT, we map the signaling network within disseminating glioblastoma cells from patient-derived xenografts implanted in mice. Disseminating tumor cells undergo a proteome-wide shift from proliferative to mesenchymal, neural progenitor-like cell states. In parallel, signaling network and global kinase activity are rewired, transitioning from cell cycle-associated circuitries to those governing synaptic function, neuronal migration, and ion channel activity. Changes begin at the tumor margin and persist in distant brain parenchyma. Hornerin and phosphorylation of Ca²⁺-permeable GluA2 at Y876 were identified as mediators of glioblastoma progression. INSIGHT enables systems-level dissection of cell-type-specific signaling circuitries in vivo across wide range of biological systems.

## Abstract (web summary)

Glioblastoma recurrence is driven by rare tumor cells that disseminate into the brain, but their signaling programs in vivo are poorly defined. Here, authors develop INSIGHT to map cell-type-specific signaling networks and show that disseminated cells rewire signaling network and global kinase activity toward neuronal and invasion associated pathways.

## Introduction

Dysregulation of intracellular signaling networks drives cancer^1,2^. Cellular signaling is mediated by dynamic phosphorylation and dephosphorylation of proteins by kinases and phosphatases primarily on tyrosine, serine, and threonine residues, which directly modulate protein function^3–5^. The significance of phosphorylation in cancer is underscored by kinase inhibitors representing one of the largest classes of FDA-approved cancer drugs^6^. Currently, the systems-level analysis of phosphorylated proteins, or phosphoproteomics, is best achieved by mass spectrometry. However, due to the rapid dynamics of phosphorylation^7^, phosphoproteomics requires rapid sample processing^8,9^, which restricts the analysis to bulk tissues. This obscures the signaling networks originating from rare and diverse cell subpopulations, leaving a substantial blind spot in our understanding of heterogeneous cellular functions and intercellular communications that collectively shape the biological response in vivo. In particular, the low cellular stoichiometry of tyrosine phosphorylation ( < 1% of the phosphoproteome^10^), requires a large sample input (1–15 mg^11,12^) for informative analysis, preventing in-depth phosphotyrosine phosphoproteomic analysis at the resolution of single-cell or low numbers of cells in vivo. Phosphoprotein-specific antibody-based approaches like multiplexed tissue imaging^13^ do not allow unbiased discovery of signaling networks. Thus, these technical barriers have impeded the systems-level elucidation of how signaling networks of distinct cell subpopulations contribute to cancer progression in vivo.

Glioblastoma (GBM) remains a lethal cancer, with recurrence driven by invasive subpopulations of tumor cells that disseminate brain tissue and evade surgical resection, rendering even drastic interventions like hemispherectomy ineffective^14^. GBM cells migrate along anatomical structures including white matter tracts as singlets or clusters^15^. Remarkably, preclinical GBM patient-derived xenograft (PDX) tumor cells retain this behavior, with rare, discrete tumor subpopulations disseminating throughout the mouse brain^16^. Seminal studies have established that GBM cells exploit both neuronal program-dependent and independent mechanisms to disseminate^17–22^. Yet, we lack a systems-level understanding of how the signaling network of GBM orchestrates dissemination in vivo, how signaling circuitries are connected within disseminated cells, and which kinases are driving dissemination of tumor cells from the core tumor. This critical knowledge gap has limited our ability to comprehensively exposing the therapeutic vulnerabilities of GBM.

In this work, to enable the quantification of signaling networks in specific cell subpopulations within complex tissues, including that of rare, disseminated tumor cells that underlie GBM recurrence, we demonstrate INSIGHT (INvestigating SIGnaling network of specific cell subpopulation in Heterogeneous Tissue), a discovery-based phosphoproteomic and protein expression analysis platform technology that combines ultra-sensitive liquid chromatography-tandem mass spectrometry (LC-MS/MS) and targeted fluorescence-activated cell sorting (FACS) with broad applicability. In INSIGHT, physiological phosphorylation sites are preserved by freezing and subsequently fixing before dissociation and cell sorting, enabling systems-level characterization of signaling networks within rare or discrete targeted cell subpopulations. We demonstrate the utility of INSIGHT by analyzing the oligodendroglial cell-specific signaling network in the mouse brain. We then apply INSIGHT to invasive GBM PDX models, and quantify thousands of phosphoproteins and proteins that compose the signaling network of rare, disseminated GBM subpopulations. We capture interconnections between their diverse signaling circuitries, validate key phosphoproteins altered during dissemination using microscopy, and infer their active kinases to reveal possible molecular mechanisms underpinning GBM dissemination in vivo.

## Results

### Phosphoproteome and whole proteome are preserved by fixation and remains stable through enzymatic dissociation to enable antibody- or fluorescence tag-based cell sorting

**Fig. 1:**
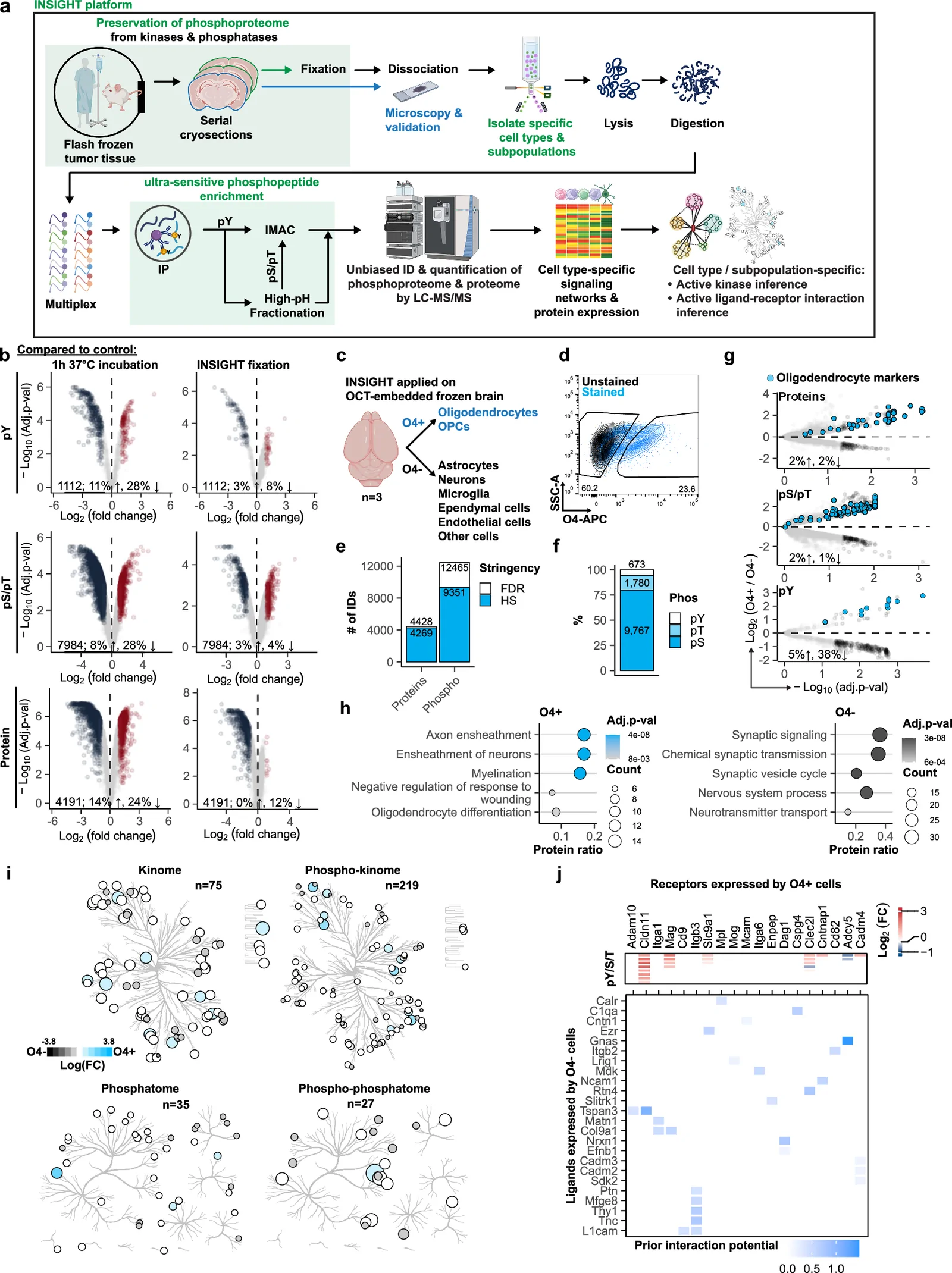
INSIGHT enables cell subpopulation-specific signaling network and whole proteome analysis in vivo. **a** Schematic of the INSIGHT platform. Flash-frozen tissue is cryosectioned, fixed, dissociated into single cells, and sorted by FACS using fluorophore-conjugated antibodies or endogenous fluorophores. Proteins are digested, tandem mass tag-labeled, and analyzed by LC-MS/MS. Phosphotyrosine peptides are enriched by immunoprecipitation and IMAC, whereas phosphoserine and phosphothreonine peptides undergo high-pH fractionation and IMAC enrichment. Part of the figure was created with BioRender. **b** Differential abundance of phosphopeptides and proteins relative to flash-frozen mouse brain tissue. Total identifications and percentages of significantly up- and downregulated IDs (fold-change ≥ 2, adjusted *p* ≤ 0.05) are shown. **c** O4-positive oligodendroglial cells were sorted from O4-negative cells in mouse brains. Part of the figure was made using BioRender. **d** Representative FACS profile of O4+ and O4− populations showing side scatter area and O4 fluorescence intensity. **e** Numbers of proteins and phosphopeptides identified at 1% FDR and high-stringency filtering. **f** Percentages of pY, pS, and pT phosphopeptides identified in (**e**). **g** Differentially expressed phosphopeptides and proteins between O4+ and O4− cells. The upper quadrant represents IDs enriched in O4+ cells. Oligodendrocyte markers are shown in blue. Differentially expressed IDs (1.5-fold change, adjusted *p* ≤ 0.05) are shown in black, with percentages indicated. **h** Top enriched biological processes in O4+ and O4− proteomes based on differentially expressed proteins (1.5-fold change, adjusted *p* ≤ 0.05). Counts indicate matched genes. **i** Kinome and phosphatome tree depicting the level of O4-positive and O4-negative enriched total and phosphorylated kinases and phosphatases. Node sizes are based on the log-transformed fold change of O4-positive to O4-negative expression data. **j** Heatmap showing predicted receptor-ligand interactions between O4+ and O4− cells involved in myelin maintenance using NicheNet. Relative pY, pS, and pT abundance for receptors identified in O4+ cells is shown above. Source data are provided as a Source Data file. For (**b**, **g**), statistical significance was assessed using FDR-adjusted two-sided *P*- values. For **h**, adjusted P values were calculated using the Benjamini-Hochberg method.

To enable the signaling network analysis of discrete cell populations of interest in vivo, we established INSIGHT, a platform technology that preserves the physiological phosphorylation throughout the analysis^7–9^. INSIGHT uses frozen tissue, which is cryosectioned at −20 °C, fixed with a commercial fixative, and enzymatically and physically dissociated into single cells to allow the sorting of antibody-stained or fluorescently-labeled specific cell types before phosphoproteomic and protein expression analyses by ultra-sensitive LC-MS/MS (Fig. 1a). To establish this workflow, we first evaluated the effectiveness of fixation in preserving physiological phosphorylation sites compared to the rapid standard sample processing workflow for LC-MS/MS and conventional tissue dissociation conditions. Frozen brain tissue sections were subjected to five distinct conditions: (1) 37 °C for 2 h, (2) 37 °C for 1 h, (3) cold PBS rinse, (4) 10% buffered formalin fixation, and (5) Cytofast (Biolegend) fixation (Fig. 1b, Suppl. Fig. 1a, b). The 37 °C incubation, common in single-cell tissue studies^23^, significantly altered protein expression and phosphorylation compared to the control, whereas fixation methods, required for preserving physiological signaling, were associated with distinct, smaller magnitude changes in both the phosphoproteome and the whole proteome (Fig. 1b). Across all conditions, GO terms associated with metabolic processes were altered compared to control (Suppl. Fig. 1b). The preserving effect of fixation for the whole proteome was consistent with a recent in vitro study^24^. Next, to find the most effective fixative, we benchmarked 10% buffered formalin and Cytofast to four other fixatives: 1.5% paraformaldehyde in PBS, Cytofix (BD Biosciences), Fix/Perm (Biolegend), and Lyse/Fix (BD Biosciences) (Suppl. Fig. 1c). We observed that Cytofast showed superior identification of antibody-targeted cells and recovery during dissociation while preserving the phosphoproteome (Suppl. Fig. 1a–c). Therefore, Cytofast was selected as the fixative for the rest of this study.

Next, to dissociate the fixed tissues for downstream cell-type sorting, we evaluated the impact of enzymatic dissociation on the phosphoproteome and whole proteome of fixed tissues to ensure that they do not degrade peptides before LC-MS/MS analysis. After testing collagenase I, II, III, and IV, correlation analysis showed that collagenase III best preserved the fixed peptides (Suppl. Fig. 2a), leading us to use it for the rest of this study. To assess the integrity of the cells and the protein loss from INSIGHT-induced membrane permeabilization or disruption, the molecular weight distribution of proteins and phosphoproteins quantified by MS in dissociated and undissociated cells was analyzed (Suppl. Fig. 2b). No significant differences were found, indicating that any depletion was likely not due to the selective loss of smaller proteins from INSIGHT-induced membrane permeabilization or leakiness. Regardless, processing led to the depletion of cell projection, vesicular, and cytoplasmic proteins, likely due to sectioning and plasma membrane permeabilization by Cytofast, with the accompanying enrichment of nuclear, plasma membrane, endoplasmic reticulum, and mitochondrial proteins, and associated kinases (Suppl. Fig. 2c, d). We also quantified cell yields from the mouse liver, lung, spleen, and brain, demonstrating the potential applicability of INSIGHT across different tissues (Suppl. Fig. 2e).

To confirm that epitopes remain intact for cell isolation by antibody-based sorting and that INSIGHT does not cause nonspecific binding or background signals during flow cytometric analysis, we tested the approach on intracranially injected, epidermal growth factor receptor (EGFR)-amplified, TdTomato-tagged GBM PDX tumors (Suppl. Fig. 2f). Anti-human EGFR and anti-HLA-A/B/C antibodies specifically bound the TdTomato+ cells in the dissociated PDX-bearing mouse brain, confirming the preservation of surface protein epitopes with no detectable unspecific binding (Suppl. Fig. 2f). Cytoplasmic pan-cytokeratin was also detected in 4T1 breast cancer cells from tumor-bearing mice (Suppl. Fig. 2g). In addition, INSIGHT was performed using CD15, a marker expressed in subsets of glioblastoma cells, together with the myeloid cell marker CD84, in glioblastoma patient tumor tissue specimens (Suppl. Fig. 3a–d). Collectively, these results highlight that fluorescently tagged proteins or cytoplasmic proteins may be targeted by INSIGHT to characterize the phosphoproteome and protein expression of cells, broadening the applicability of INSIGHT in genetically engineered model organisms.

### INSIGHT can map cell type-specific signaling networks in vivo

As a proof of principle to demonstrate that INSIGHT can characterize the signaling networks of specific cell subpopulations from tissues, we focused on oligodendrocyte lineage cells of the brain. Oligodendrocyte lineage cells (oligodendrocytes and oligodendrocyte progenitor cells), serve critical roles in myelination and provide metabolic support to neurons and tumors^25,26^. We leveraged the cell-specific marker O4 antigen to isolate O4-positive oligodendrocyte lineage cells and O4-negative cells for analysis by INSIGHT (Fig. 1c, d). This resulted in the identification of 4428 unique proteins and 12,465 phosphopeptides at a 1% FDR threshold. To ensure high-quality phosphopeptide and protein identification, peptide spectral matches (PSMs) were filtered using a high-stringency (HS) ion score cutoff of 20, established through extensive manual validation of spectra^27^. This refinement yielded 4,269 proteins and 9,351 phosphopeptides (Fig. 1e), comprising 9,767 phosphoserine (pS), 1,780 phosphothreonine (pT), and 673 phosphotyrosine (pY) sites (Fig. 1f). Oligodendrocyte marker proteins were specifically enriched in O4-positive proteome and phosphoproteome (Fig. 1g). Moreover, O4-positive cells were enriched in oligodendroglial processes, while O4-negative cells were enriched in neuronal processes (Fig. 1h). Collectively, these results confirmed that INSIGHT successfully profiled the signaling network of enriched oligodendrocyte lineage cells from fixed brain tissues. Furthermore, INSIGHT mapped O4-positive and negative cell-specific expression of kinases, phosphatases, and their phosphorylated forms, revealing their expression patterns and potential activation status (Fig. 1i). Next, leveraging cell-type specific protein expression data, we used NicheNet^28^ to infer ligand-receptor interactions between O4-negative and O4-positive cells that may regulate myelin maintenance within O4-positive cells. We then integrated ligand-receptor interaction predictions with receptor phosphorylation data to further refine the predicted interaction landscape. This strategy enabled us to infer potential ligand-receptor signaling dynamics across the two subpopulations that contribute to myelin maintenance (Fig. 1j), highlighting the potential of INSIGHT in uncovering physiologically relevant cell-cell communication mechanisms at the protein and phosphoprotein-level. Taken together, we validated the specificity of the isolation strategy of INSIGHT by mapping the signaling network of oligodendroglial cells and demonstrated the utility of INSIGHT in inferring physiologically relevant information in vivo.

### Signaling network of rare, disseminated glioblastoma cells is uncovered by INSIGHT

**Fig. 2:**
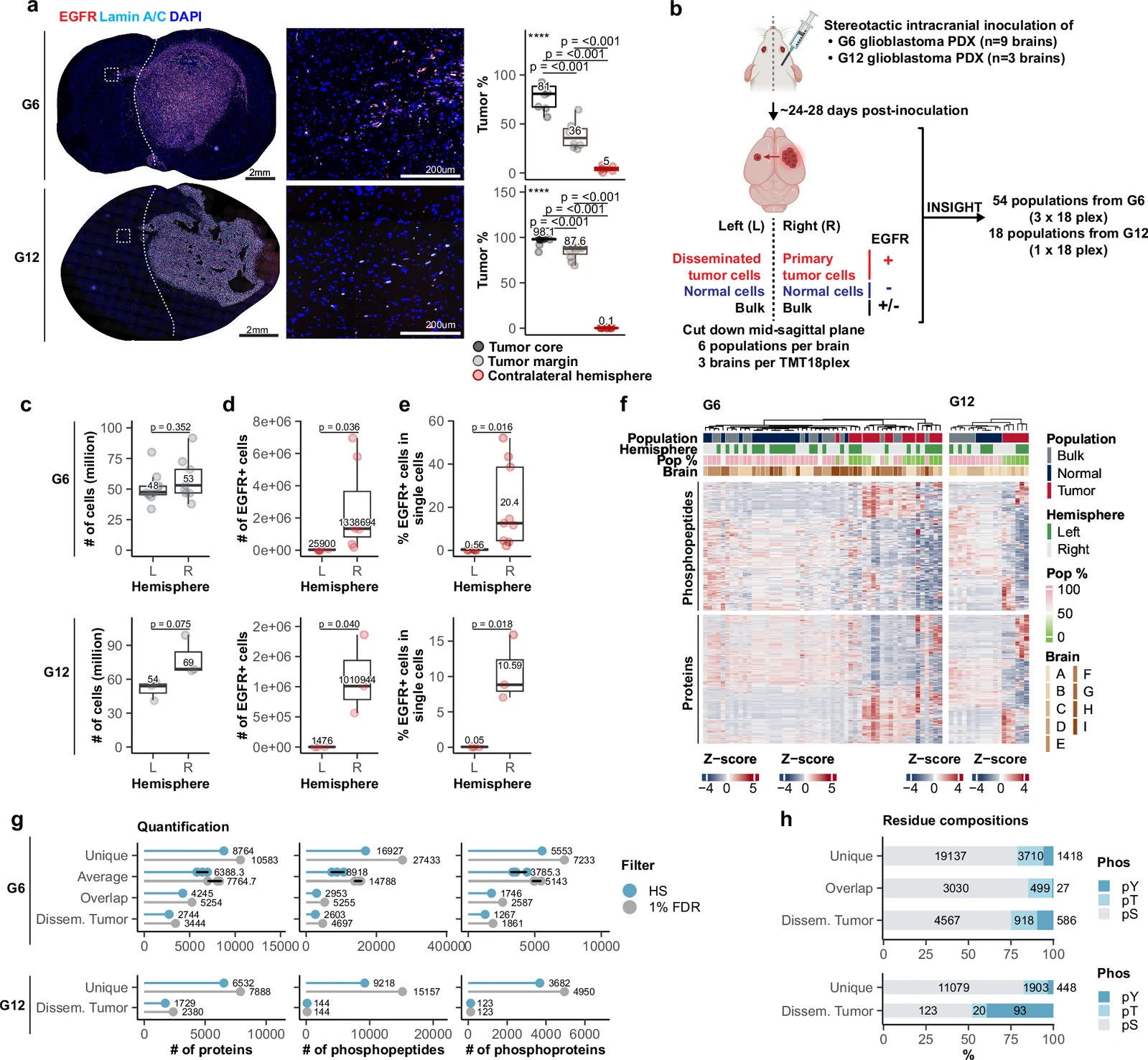
Application of INSIGHT on GBM PDX models. **a** Representative coronal sections from G6 and G12 GBM PDX-bearing brains stained for human-specific EGFR, human-specific Lamin A/C, and DAPI. White dotted lines indicate the brain midline. Middle panels show magnified regions. Tumor cell percentages (EGFR+ Lamin A/C + DAPI + ) were quantified in the primary tumor core, tumor margin, and left hemisphere containing disseminated tumors. Scale bars, 2 mm (whole section) and 200 μm (magnified). Statistical significance was determined using two-sided one-way ANOVA with Tukey’s multiple comparisons adjustment. Biological replicates: G6, contralateral hemisphere, *n* = 7; core, *n* = 8; margin, *n* = 8. G12 contralateral hemisphere, *n* = 12; core, *n* = 12; margin, *n* = 11. **b** Experimental schematic. EGFRvIII-amplified G6 and EGFR-amplified G12 GBM PDXs were stereotactically implanted into the right hemisphere of mice (*n* = 9 and *n* = 3, respectively). After 24–28 days, disseminated tumor cells migrated into the left hemisphere. Hemispheres were dissociated, stained with human-specific EGFR antibody, sorted by FACS, and analyzed by LC-MS/MS phosphoproteomics and proteomics. Part of the figure was created with BioRender. **c** Total cells recovered from left and right hemispheres of G6 (*n* = 9) and G12 (*n* = 3) tumor-bearing brains. **d** Number of EGFR+ cells retrieved from G6 (*n* = 7) and G12 (*n* = 3) by FACS. Median values are annotated. **e** Percentage of EGFR+ cells in single cells from G6 (*n* = 7) and G12 (*n* = 3) by FACS. Median values are annotated. **f** Hierarchical clustering of G6 and G12 samples processed by INSIGHT using identified proteins and phosphopeptides. Only overlapping high-stringency identifications were included. PSMs with missing values were removed except for disseminated tumor-specific IDs, where missing values were imputed for comparison. Pop % indicates population percentage relative to total sorted single cells. **g** Numbers of proteins, phosphopeptides, and phosphoproteins passing high-stringency and 1% FDR filtering across G6 and G12 experiments, including unique IDs, shared IDs across G6 runs, and IDs detected in disseminated tumor cells. Mean ± SD. **h** Percentages of pY, pS, and pT phosphopeptides passing high-stringency filtering in (**g**). Numbers indicate uniquely mapped IDs, shared IDs, and disseminated tumor-specific IDs across three runs. Source data are provided as a Source Data file. Box plots show median, quartiles, and 1.5× interquartile range whiskers. For Fig. (**c**–**e**), statistical significance was assessed using two-sided pairwise comparisons.

Having demonstrated the feasibility of INSIGHT for cell subpopulation-specific phosphoproteomics, we leveraged INSIGHT to characterize the signaling networks of invasive, disseminated EGFR-amplified GBM cells in vivo. EGFR overexpression, in its wild-type or constitutively active EGFRvIII mutant form, is found in over 40% of GBM patients and drives tumorigenicity^29,30^. We used two PDX models, GBM6 (G6) and GBM12 (G12), derived from untreated primary IDH-wildtype GBM^31^. G12 harbors wild-type EGFR amplification, whereas G6 carries an EGFRvIII mutation^32,33^. Implantation of PDX tumors into the right hemispheres of mice led to contralateral tumor dissemination via white matter tracks of the corpus callosum as seen in patients^34^ (Fig. 2a). Notably, G6 primary tumor cells showed greater tumor spread, more diffuse boundaries, and higher normal cell content compared to G12 primary tumor cells at the coronal section with the highest primary tumor burden (Fig. 2a, Suppl. Fig. 3e-f), indicating a more invasive phenotype. We then set out to isolate and analyze the tumor cells from each hemisphere by enriching for human EGFR-expressing cells (Fig. 2b). To do so, G6 and G12 PDX tumor-bearing brains were resected at moribund, flash-frozen, and cut down the mid-sagittal plane. Each hemisphere was cryosectioned, fixed, and dissociated to yield single cells (Fig. 2c). Cells were then stained with a human-specific anti-EGFR antibody that also detects EGFRvIII and sorted to isolate EGFR-positive rare, disseminated tumor cells ( < 1%) from the left hemisphere and EGFR-positive primary tumor cells from the right hemisphere (Fig. 2b, d, e, Suppl. Fig. 3g, h). The higher number of disseminated cells in G6 compared to G12 corroborated the co-immunofluorescence (co-IF) staining result (Fig. 2a, e, Suppl. Fig. 3e, f). Additionally, EGFR-negative cells (non-malignant cells; “normal cells”) and bulk unsorted mixtures of cells were taken from each hemisphere for comparison. In total, we analyzed nine G6 tumor-bearing brains and three G12 tumor-bearing brains, all of which were subjected to phosphoproteomic and protein expression analysis via LC-MS/MS (Fig. 2b).

Upon unsupervised hierarchical clustering of the MS data, primary and disseminated tumors clustered together and away from bulk mixtures and normal cells, indicating that the signaling profiles of tumor cells, regardless of location, were similar to each other and distinct from normal cells (Fig. 2f). The bulk cells clustered with EGFR-negative normal cells, suggesting that a significant proportion of unsorted cells consisted of normal cells, as expected. Across all G6 samples, we identified 8764 unique proteins, with an average of 6389 proteins across plexes and 4245 proteins detected in all plexes (Fig. 2g). In G12, we identified 6532 unique proteins and 9218 phosphopeptides (Fig. 2g). The HS-filtered data contained 24,265 unique phosphosites across 16,927 phosphopeptides mapping to 5553 proteins across all G6 runs, while in G12, 13,430 unique phosphosites across 9218 phosphopeptides were mapped to 3,682 proteins (Fig. 2g, h). When analyzing only those IDs that were expressed in disseminated tumor cells, 2,744 and 1729 unique proteins were detected in G6 and G12, respectively, and 2603 and 144 unique phosphopeptides were identified in G6 and G12, respectively (Fig. 2g). Thus, despite limited cell numbers, INSIGHT enabled systems-level quantification of phosphopeptides and proteins from both tumor and normal cells in the left and right hemispheres.

### INSIGHT quantifies and differentiates the signaling networks of tumor and normal cells in PDX models to enable the inference of tumor-specific kinase activity

**Fig. 3:**
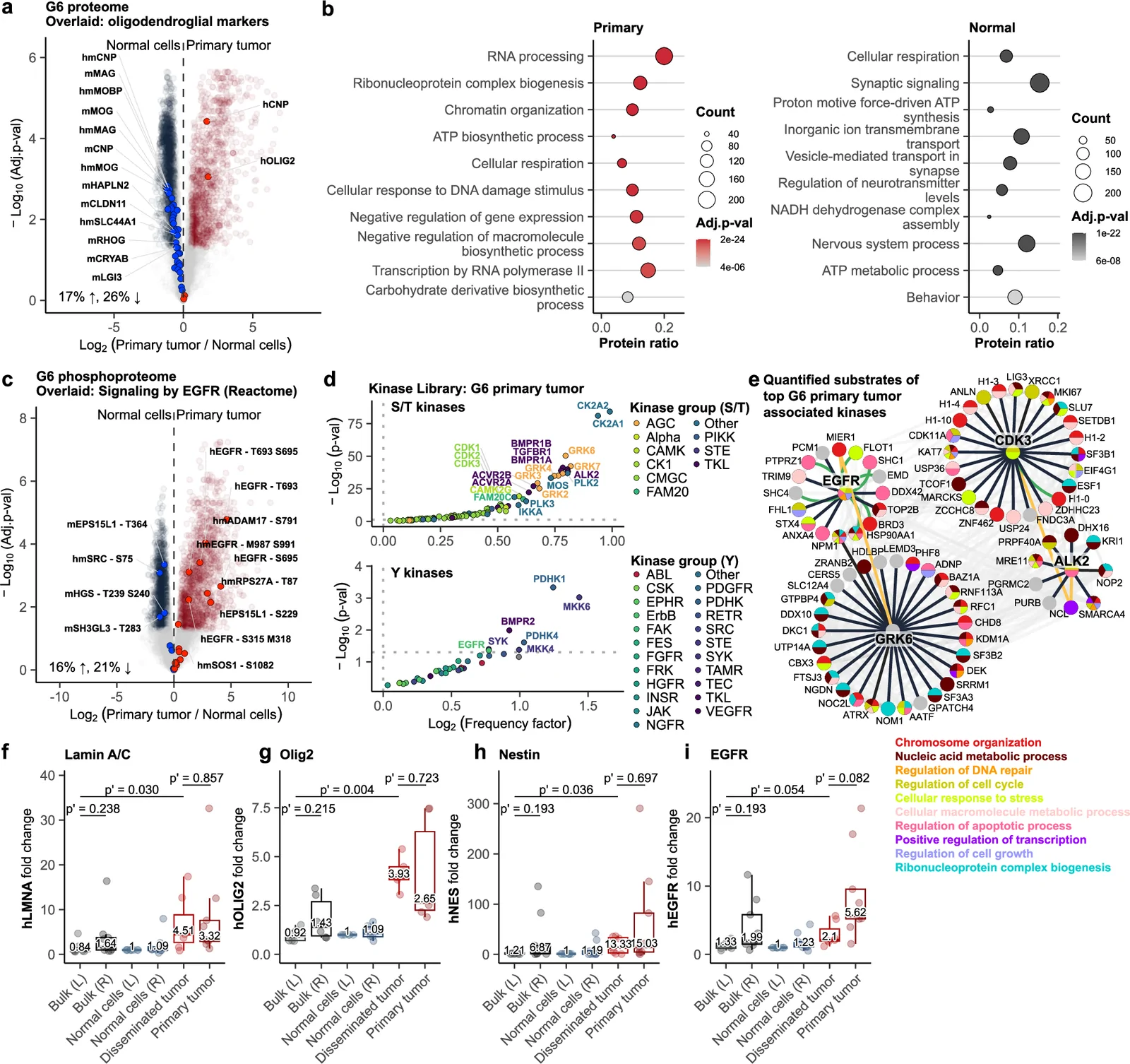
The signaling networks of primary tumor cells and normal cells in vivo assessed by INSIGHT. **a** Differentially enriched proteins between EGFR-positive primary tumor cells and normal cells from the left hemisphere of the G6 PDX model, with oligodendrocyte markers overlaid. The percentages of significantly differentially expressed IDs are shown in the bottom left corner. Lowercase prefixes indicate proteins specific to humans (h), mice (m), or shared between humans and mice (hm). **b** Biological processes enriched in G6 primary tumor cells and normal cells from the right hemisphere based on their protein expression. **c** Differentially enriched phosphopeptides in primary tumor cells and normal cells from the right hemisphere of the G6 PDX model. EGFR signaling components (Reactome) are labeled. Prefixes indicate human-specific (h), mouse-specific (m), or shared (hm) proteins. **d** Phosphopeptides enriched in G6 primary tumors versus normal cells were analyzed using Kinase Library^4^ to predict active kinases. **e** Proteins whose phosphorylations were predicted by Kinase Library to be mediated by top-enriched kinases in **d** were analyzed by STRING. Node colors indicate enriched biological processes (adjusted *p* ≤ 0.05). Gray edges indicate known protein-protein associations, black edges indicate predicted kinase-substrate relationships, green edges indicate overlap between predicted kinase-substrate and known STRING interactions, and yellow edges indicate proteins regulated at two phosphorylation sites by two kinases. **f**–**i** Levels of human Lamin A/C (LMNA), Olig2, Nestin (NES), and EGFR across bulk cell mixtures, normal cells (EGFR-), and EGFR+ tumor cell populations from the left (L) and right (R) hemispheres of the G6 PDX model by INSIGHT. Missing values in cells other than disseminated tumor cells were imputed with the lowest m/z intensity in each PSM at the level of each TMT experiment to enable comparison. Adjusted *P*-values were calculated using the BH method. Following the concatenation of three G6 TMT experiments, all samples detecting Lamin A/C, Nestin, EGFR (primary *n* = 9, disseminated *n* = 7), and Olig2 (primary *n* = 6, disseminated *n* = 5) were plotted. Source data are provided as a Source Data file. Box plots show median, quartiles, and 1.5× interquartile range whiskers. For Fig. (**a**, **c**, **f**–**i**), significance was assessed using BH-adjusted two-sided *P*-values. For Fig. (**b**, **d**), one-sided *P*-values were BH-adjusted.

To evaluate whether the INSIGHT workflow effectively identified the tumor cell-specific signaling network, we performed differential expression analysis of the phosphoproteome and proteome between primary tumor cells and normal cells that were spatially intermixed within the same tissue, a task that has been previously impossible with tissue phosphoproteomics. Pronounced differences were observed between the proteome of tumor cells and normal cells in both G6 and G12 models, as expected (Fig. 3a, Suppl. Fig. 4a). Importantly, murine oligodendrocyte-specific proteins were specifically enriched in the murine normal cell proteome, while human Olig2 was expressed by human primary G6 tumor cells, consistent with a previous report^35^. Moreover, primary tumor cells of both PDX models upregulated biological processes related to enhanced transcriptional and proliferative activity (Fig. 3b, Suppl. Fig. 4b). In contrast, normal cells were enriched in GO terms reflecting metabolic homeostasis and neurobiological functions of brain cells (Fig. 3b, Suppl. Fig. 4b). These results indicate that INSIGHT successfully separated human tumor cells from the murine normal brain cells, which has been previously impossible in tissue phosphoproteomics of PDX models^36^.

The phosphoproteome of primary tumor cells also significantly differed from that of normal cells in both PDX models (Fig. 3c, Suppl. Fig. 4c). Still, a large proportion of phosphopeptides were shared between the tumor cells and normal cells in both PDX models based on the percentage of statistically significant up- and down-regulated phosphopeptides, highlighting that their accurate quantification would be impossible without physical separation using INSIGHT. Importantly, phosphorylated EGFR was significantly enriched in tumor cells, consistent with EGFR amplification in both PDX models, underscoring the effectiveness of INSIGHT in isolating tumor-specific phosphoproteome in vivo (Fig. 3c, Suppl. Fig. 4c). Using the quantified tumor phosphoproteome and the Kinase Library (contains sequence motif specificities of the human kinome^4,5^), the most active kinases in tumor cells were inferred based on the prevalence of their phosphorylated substrates (Fig. 3d, Suppl. Fig. 4d). This analysis predicted EGFR as top-ranking tyrosine kinase (Fig. 3d bottom, Suppl. Fig. 4d bottom), consistent with EGFR amplification in both PDX models. Additional serine/threonine kinases were predicted to be enriched in GBM cells, including G protein-coupled receptor kinases (GRKs) and cyclin-dependent kinases (CDKs)^37–40^ as well as others that may contribute to GBM progression. Proteins with phosphorylation sites predicted to be mediated by these kinases showed significant enrichment in interconnected, tumor-related biological processes, highlighting extensive crosstalk in signaling circuitries that are regulated by top-predicted kinases (Fig. 3e). Together, INSIGHT enabled the differentiation and quantification of signaling networks in normal and tumor cells within PDX models, allowing for the inference of potentially active kinases specific to tumor cells.

### Disseminated tumor cells retain tumor-specific marker expression while downregulating EGFR and pT693-EGFR relative to primary tumor cells

Next, we assessed the expression of previously reported tumor-specific markers^35,41^ (Olig2, Nestin, and Lamin A/C) and found they were comparably expressed in both G6 primary and disseminated tumors, confirming marker retention at distant sites and successful tumor cell isolation (Fig. 3f–h). Intriguingly, EGFR expression (mostly EGFRvIII in G6^33^) and EGFR phosphorylation at the T693 residue, e.g., pT693-EGFR, showed a downward trend in the disseminated tumor cells relative to primary tumor cells by INSIGHT, flow cytometry, and co-IF (Fig. 3i, Suppl. Fig. 5a-f). Notably, G6 disseminated tumor cells migrating as singlets exhibited lower, but detectable, EGFR and pT693-EGFR expression compared to those traveling in clusters, suggesting that the EGFR pathway is downregulated when cells disperse (Suppl. Fig. 5c–f). The downregulation of EGFRvIII expression in primary GBM at recurrence has been reported in patients^42–44^, drawing parallels between G6 disseminated tumors and the recurrent patient tumors originating from infiltrating cells post-surgery. Reductions in EGFR and pT693-EGFR levels were also seen in G12 disseminated tumor cells compared to G12 primary tumor cells using microscopy, flow cytometry, and INSIGHT (Suppl. Fig. 6a–d). Furthermore, disseminated G12 tumor cells, unlike G6, showed reduced Lamin A/C expression compared to primary cells by both INSIGHT and co-IF, while housekeeping proteins remained stable (Suppl. Fig. 4e, f). These findings reveal differences between disseminated G6 and G12 cells. EGFR downregulation was reported to facilitate epithelial to amoeboid transition during tumor invasion^45^, suggesting that EGFR signaling downregulation may be part of the G6 and G12 dissemination process regardless of EGFR mutation status. Results across multiple modalities revealed EGFR signaling suppression in disseminating GBM cells, confirming the robustness of INSIGHT and suggesting that disseminated PDX cells partially resemble recurrent patient GBM.

### Disseminated GBM cells engage neuron-associated programs and signaling networks

**Fig. 4:**
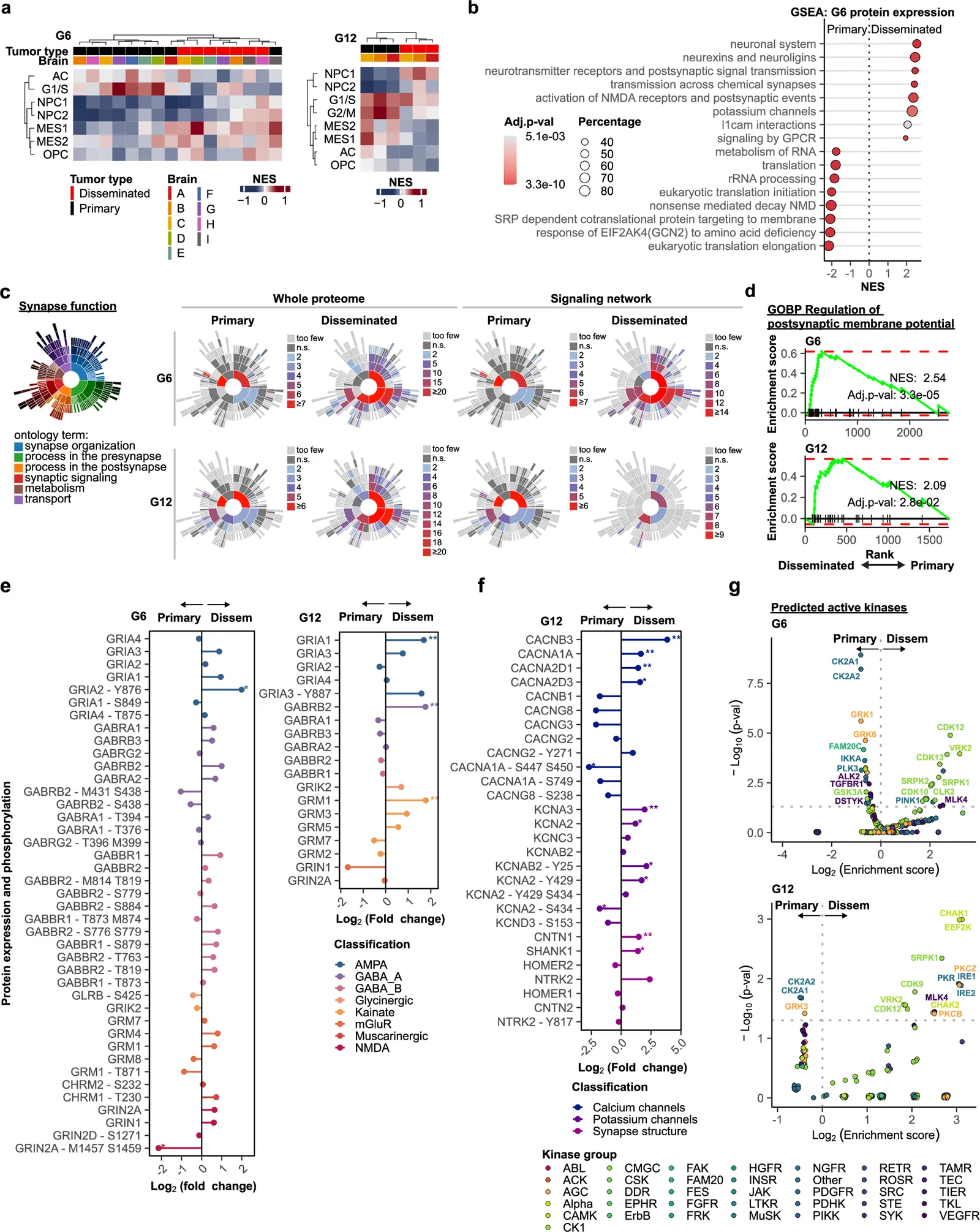
Global changes in signaling networks and whole proteome between disseminated and primary glioblastoma in vivo. **a** Glioblastoma cell state module^46^ enrichment scores (NES) of primary and disseminated tumor cells based on their whole proteome. NPC (neural progenitor cell-like), OPC (oligodendrocyte progenitor-like), MES (mesenchymal cell-like), G1/S, G2/M, and AC (astrocyte-like) signatures were used. **b** GSEA results on the proteins quantified in the primary and disseminated G6 tumors (log_2_ protein expression ratios) based on the Reactome database. **c** Ontology analysis of synaptic functions (annotated by SynGO^129^) in primary and disseminated tumor cells based on their whole proteomes and signaling networks. Sunburst plot (left) illustrates the first-level ontology terms. Significantly enriched (-log10 q-value) ontology terms are colored. **d** GSEA plots for the gene set “GOBP Regulation of postsynaptic membrane potential” comparing disseminated versus primary tumor cells using their proteome abundance. Normalized enrichment score (NES) is shown on the y-axis, with the ranked list of genes from disseminated (left) to primary (right) on the x-axis. The green line indicates the running enrichment score, while the vertical black lines represent individual genes contributing to the enrichment. **e** Relative protein and phosphoprotein expression of neurotransmitter receptors between disseminated and primary tumor cells. * = adj.p-val ≤ 0.05. **f** Relative protein and phosphoprotein levels of ion channels between disseminated and primary G12 tumor cells. * = adj.p-val ≤ 0.05. **g** Active serine, threonine, and tyrosine kinases were predicted in primary and disseminated tumor cells using the Kinase library. Source data are provided as a Source Data file. For (**b**, **d**), statistical significance was assessed using permutation-based one-sided Kolmogorov-Smirnov-like enrichment statistics with false discovery rate correction for multiple comparisons. For (**e**, **f**), statistical significance was assessed using FDR-adjusted two-sided P values. For (**g**), Statistical significance was calculated using one-sided enrichment-based P values with Benjamini-Hochberg correction.

The proteome-level cell states of primary and disseminated GBM cells in vivo have remained unknown, leaving questions about the degree of tumor cell plasticity and its functional connection to invasive tumor cell behavior in vivo. Therefore, we leveraged the whole proteome data from INSIGHT to analyze the enrichment of GBM cell state modules^46^. GBM cells shifted from G1/S or G2/M to mesenchymal (MES), neuronal progenitor-like (NPC), or oligodendrocyte progenitor-like (OPC) cell states in both PDX models upon dissemination, demonstrating that dissemination is coupled to a coordinated exit from proliferative programs and entry into lineage- and invasion-associated cell states (Fig. 4a). Disseminated tumor cells did not show consistent enrichment for published stemness gene signatures derived from tumor cores or in vitro models (Suppl. Fig. 7a). Furthermore, Gene Set Enrichment Analysis (GSEA) on the whole proteome revealed that signatures related to neuronal programs, including postsynaptic signal transmission, neurotransmitter receptors, and potassium channels were significantly enriched in disseminated cells compared to primary cells in both PDX models (Fig. 4b, Suppl. Fig. 7b–d). Recent studies have demonstrated that GBM cells can form functional synapses with neurons and/or co-opt neuronal programs to promote tumor progression, critically affecting GBM patient outcome^11,17–19,22^. Importantly, although GBM has been shown to express both presynaptic and postsynaptic proteins^47–49^, their general function during GBM progression remain an active area of investigation. We therefore examined the systems-level abundance changes in phosphoproteins and proteins involved in synaptic functions, as annotated by SynGO (Fig. 4c). The differences between G6 and G12 may have arisen from both technical and biological sources. Separate mass spectrometry experiments can introduce batch effects and stochastic missing values, while genuine differences in proteome and phosphoproteome compositions can further reduce the overlap by altering peptide detectability (Suppl. Fig. 7e, f; Supplementary Data 6). Nevertheless, proteins and phosphopeptides that consistently overlapped provided valuable insight into shared regulatory programs. Based on synaptic component location, both bulk RNAseq and INSIGHT quantified a broad spectrum of presynapse and postsynapse-associated proteins and phosphoproteins in the two PDX models (Fig. 4c, Suppl. Fig. 7e–g). While primary tumor cells were preferentially enriched in synapse-associated metabolic functions, disseminated tumor cells exhibited significant enrichment of postsynaptic, presynaptic, and synaptic signaling processes compared to primary tumor cells, highlighting a convergent remodeling of the synaptic signaling machinery with GBM dissemination in vivo irrespective of EGFR mutational status (Fig. 4c).

### Synapse and invasion-associated signaling circuitries converge

Concordantly, the ‘regulation of postsynaptic membrane potential’ gene signature was enriched in disseminated cells of both PDX models compared to their primary counterparts, reinforcing that GBM cells enhance postsynaptic regulation as they disseminate (Fig. 4d). Intriguingly, top-ranked genes from GSEA contributing to the enriched postsynaptic signatures in G6 disseminated cells were linked to EPH-ephrin-mediated repulsion and ion homeostasis, which have been implicated in tumor invasiveness outside of GBM^50–52^ (Suppl. Fig. 7c-d). This observation prompted us to examine the synaptic genes in signatures related to tumor spread, including migration, invasion, and epithelial-to-mesenchymal transition (Suppl. Fig. 8a). Querying the MSigDB database using keywords, we found that 45% of synaptic genes overlapped with the genes in tumor dissemination-related signatures. Moreover, phosphoprotein such as MAPK1, DLG2 differentially expressed in disseminated cells compared to primary tumors appeared in multiple signatures (Suppl. Fig. 8b). These findings highlight an unrecognized extent of crosstalk between signaling circuitries governing tumor invasiveness and neuronal programs.

### Neurotransmitter receptor signaling is rewired during GBM dissemination

The capacity of glioma cells to express neurotransmitter receptors and integrate rapidly with neuronal circuits have been recently reported^53^. Analyzing the neurotransmitter receptors in our INSIGHT dataset revealed a statistically significant increase in the abundance of select proteins and phosphorylation across primary and disseminated GBM cells in vivo (Fig. 4e). The two PDX models showed different patterns of neurotransmitter receptor expression and phosphorylation during tumor dissemination, with G6 increasing GRIA2/GluA2-pY876 while G12 instead increasing GRIA1/GluA1, GABRB2, and GRM1 protein expressions (Fig. 4e). Additionally, G12 tumors exhibited statistically significant alterations in the expression and phosphorylation of multiple ion channel-associated proteins, distinguishing them from G6 tumors, which showed no significant changes (Fig. 4f). Thus, although both PDX models engaged neurotransmitter receptor signaling during dissemination, they did so through distinct signaling routes.

### Primary and disseminated glioblastoma cells have diverging global kinase activity

Systems level changes in global kinase activities between disseminated and primary GBM in vivo have remained unknown despite their critical therapeutic implication. Leveraging the phosphoproteomic data from INSIGHT, we inferred differentially active kinases between disseminated and primary tumor cells (Fig. 4g, Suppl. Fig. 8c), and revealed VRK2 and MLK4 as some of the top-ranking kinases in disseminated tumor cells (Fig. 4g). VRK2 and MLK4 have been reported to be preferentially enriched in stem-like mesenchymal GBM tumors^54^, with VRK2 linked to glioma cell cycle regulation^55^ and MLK4 being essential for mesenchymal GBM stem cell survival^54^. These findings suggest that additional inferred kinases with previously uncharacterized roles in GBM may also contribute to invasive dissemination.

### Phosphorylation-driven modulation of neuronal programs underpins GBM dissemination

**Fig. 5:**
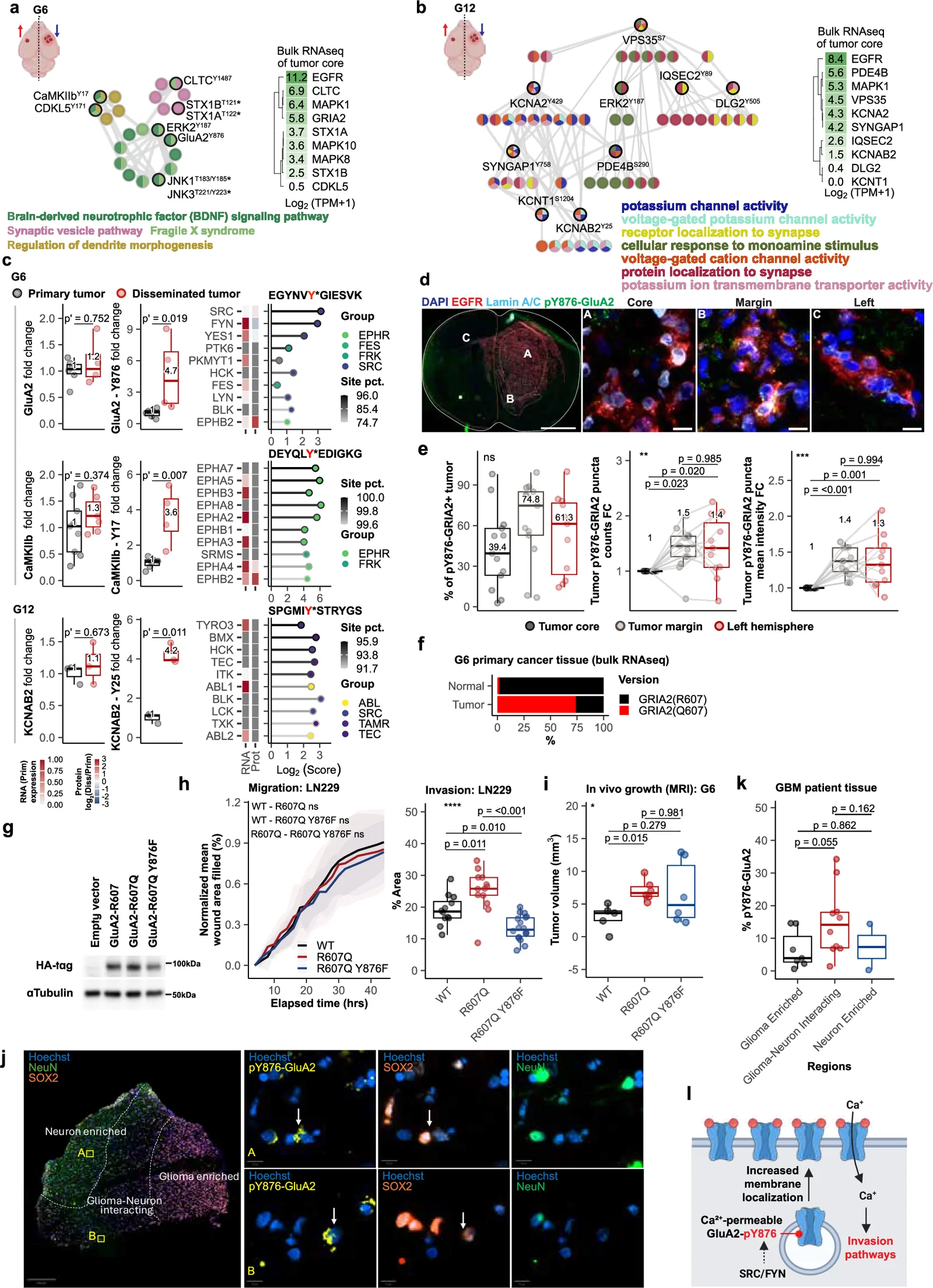
Disseminated GBM cells engage postsynaptic signaling network in vivo*.* **a** Pathway enrichment analysis of phosphoproteins upregulated in disseminated versus primary tumors in the G6 PDX model (FC ≥ 1.5, adjusted *P* ≤ 0.05). Nodes represent phosphoproteins colored by pathway membership. Related pathways sharing phosphoproteins shown as additional nodes. *Shared tryptic phosphopeptides prevented distinction between JNK1/JNK3 and STX1A/STX1B. Heatmap corresponds to human-specific transcript levels from bulk RNA-seq^31^. **b** Same as in a for G12. **c** Select phosphorylation sites and protein levels in disseminated (D) and primary (P) tumors from G6 and G12 PDX models. BH-adjusted two-sided *P*-values shown. Predicted kinases from Kinase Library shown (right). Heatmaps indicate transcript abundance and relative protein expression. Gray indicates undetected proteins or transcripts with Log₂(TPM + 1) < 0.1. Biological replicates were CAMK2B: D (*n* = 7), P (*n* = 9); pY-CAMK2B, GRIA2, and pY-GRIA2: D (*n* = 4), P (*n* = 6); and KCNAB2 and pY-KCNAB2: D and P (*n* = 3 each). **d** Co-IF of sections from G6 PDX-bearing brains. Scale bars, 10 μm (magnified) and 2 mm (whole). **e** Percentage of pY876-GluA2+ tumor cells, puncta density, and mean intensity in d. Adjusted *P*-values were determined using Tukey multiple comparisons following two-sided one-way ANOVA(*** = 2.4e-04, ** = 9.6e-03; ns = 0.21). *n* = 11 contralateral hemisphere, 13 core, and 13 margin biological replicates. **f** Percentages of GluA2(Q) and GluA2(R) in primary G6 tumors from bulk RNAseq. **g** LN229 cells expressing empty vector, HA-GluA2 variants. Experiment repeated twice. **h** Wound scratch and matrigel invasion assay of LN229. Data represent *n* = 3 technical replicates and *n* = 4 fields taken per technical replicate. Significance was assessed using linear regression with pair-wise comparisons of estimated marginal slopes. Two-sided *P*-values were calculated (0.26, 0.14, and 0.93). **i** Intracranial implantation of G6 tumor cells expressing GluA2 variants into mice, followed by MRI (*n* = 5 WT, 6 R607Q, and 6 R607Q/Y876F biological replicates). One-way Welch ANOVA with Games-Howell post hoc testing was performed. **j** CyCIF analysis of human GBM tissues showing tumor-, neuron-, and tumor-neuron interaction-enriched regions. Hoechst, pY876-GluA2, SOX2, and NeuN indicated. Arrows point to pY876-GluA2^+^ tumor cells. Scale bar = 10 μm (magnified), 400μm (whole). **k** Percent pY876-GluA2^+^ cells in *n* = 20 samples in (**j**). Kruskal-Wallis test for multiple comparisons was done. **l** Working model of GluA2-mediated GBM invasion. Source data are provided as a Source Data file. Box plots show median, quartiles, and 1.5× interquartile range whiskers. Figures (**a**, **b**, **l**) were partially created with BioRender. TPM, transcripts per million.

Phosphorylation of numerous proteins typically associated with neuronal function, including synaptic function (e.g., SYNGAP1, CaMKIIb, GluA2), neuronal migration (e.g., CDKL5, CLTC), and ion homeostasis (e.g., KCNAB2), was significantly increased in disseminated tumors compared to primary tumor cells in both PDX models (Fig. 5a, b, Suppl. Fig. 9a). Notably, this increase occurred without changes in total protein expression (Fig. 5c, Suppl. Fig. 9a–c), demonstrating that phosphorylation-driven signaling network regulation contributes to GBM dissemination in vivo (Fig. 5a–c, Suppl. Fig. 9a–c). Furthermore, in G6, total protein and doubly phosphorylated T185/Y187-ERK2 remained unchanged between primary and disseminated tumors (T185/Y187-ERK2 was undetected in G12) while pY187-ERK2 increased in both G6 and G12, indicating selective and differential phosphorylation on ERK2 during tumor spread, a phenomenon that has not yet been explored and is challenging to distinguish without mass spectrometry (Suppl. Fig. 9c). Many of these proteins exhibited moderate transcript levels in primary tumor samples in vivo relative to amplified EGFR, indicating that primary tumor cells have inherent capacity to express these proteins prior to dissemination (Fig. 5a, b).

To identify the potential upstream regulators of the phosphorylation events that are increased in disseminated tumor cells, we used Kinase Library^4,5^ to predict kinase-substrate relationships. Src was predicted to phosphorylate Y876-GluA2 (Fig. 5c), consistent with previous reports^56–58^. Src was detected by both RNAseq (primary tumor) and INSIGHT in G6 primary and disseminated tumors (Fig. 5c), supporting the potential role of Src in GluA2 phosphorylation in vivo. EPHR kinases were predicted to potentially phosphorylate Y17-CaMKIIb, implicating a potential role for their activity supporting GBM dissemination (Fig. 5c). Additionally, the phosphorylation of KCNAB2, a potassium channel recently implicated in GBM invasion^59^ and elevated in G12 disseminated tumor cells, ranked highly as a substrate of TYRO3, a kinase involved in synaptic plasticity and tumor invasion^60,61^. This places KCNAB2 and TYRO3 at the cross-section of the two signaling circuitries and implicates pY25 in GBM dissemination (Fig. 5c). Notably, disseminated G12 tumor cells exhibited significantly reduced tyrosine phosphorylation of the activation loop in GSK3A/B compared to primary G12 cells, indicating attenuation of GSK3 signaling and a shift toward signaling states associated with increased cellular plasticity and invasive potential^62^ (Suppl. Fig. 8d, Supplementary Data 7). Together, we uncovered previously uncharacterized phosphorylation sites and potential kinases involved in GBM dissemination and collectively highlight that phosphorylation-driven modulation of signaling circuitries underpins GBM dissemination.

### Y876-GluA2 phosphorylation promotes GBM invasion

Phosphorylation of Y876-GluA2 is essential for synaptic plasticity in neurons by changing the surface localization of GluA2 to the postsynaptic density^63–67^. However, its role in cancer has been unknown. Given that pY876-GluA2 significantly increased in disseminated G6 tumor cells compared to the tumor core (not observed in G12), we set out to validate our findings. By co-IF, the percentage of tumor cells that contained at least one pY876-GluA2 positive punctum did not significantly differ across tumor core, tumor margin, and the disseminated tumor cells in the left hemisphere (Fig. 5d-e, Suppl. Fig. 9d, e). However, consistent with the INSIGHT data, the number and the mean intensity of pY876-GluA2-positive puncta were significantly higher in tumor cells at the margin and the left hemisphere compared to those at the core, suggesting tumor cells may leverage pY876-GluA2 to regulate synaptic plasticity as they encounter the brain microenvironment including neurons while spreading (Fig. 5d-e, Suppl. Fig. 9d, e).

In mature neurons, the RNA editing of *GRIA2* by ADAR converts its glutamine (Q) to arginine (R), rendering it Ca^2+^-impermeable^68^. Intriguingly, glioma has been reported to under-edit *GRIA2*, thus increasing the GluA2(Q) expression^17,68^, enhancing Ca^2+^ influx, and allowing synaptic transmission-dependent tumor growth and synaptic transmission-independent invasion and migration^69,70^. Therefore, we analyzed the bulk RNAseq data of primary G6 tumor cells to verify the editing status of *GRIA2*. We observed that GluA2(Q) transcripts constituted a striking 74% of the total *GRIA2* expressed, indicating a predominant expression of Ca^2+^-permeable GluA2 by G6 tumor cells in vivo, which likely helps their growth and/or invasion (Fig. 5f). *GRIA2* was not detected in G12 primary tumor cells from bulk-RNAseq.

Based on these findings, we wanted to investigate the functional relevance of GluA2 phosphorylation at Y876 in the context of the Ca^2+^-permeable GluA2-R607Q variant in LN229 human GBM cells (Fig. 5g). Although the increased Ca^2+^-permeable GluA2 expression did not affect migration, it markedly enhanced its invasive capacity in vitro, which was significantly attenuated by the loss of pY876 through Y876F substitution (Fig. 5h). Furthermore, orthotopic implantation of GBM6 cells expressing GluA2 wild-type, the R607Q mutant, or the Y876F/R607Q mutant demonstrated that enhanced Ca^2+^ permeability via R607Q increased tumor growth, whereas the contribution of Y876 phosphorylation to tumor growth could not be conclusively established (Fig. 5i). To assess the clinical relevance of GluA2-pY876 in human GBM, we examined surgical specimens from five patients undergoing tumor resection. For each patient, two biopsies were collected from three to four spatially distinct regions, yielding a total of 20 specimens. Cyclic immunofluorescence (CyCIF) analysis of the tissue samples revealed that pY876-GluA2^+^ cells were enriched in tumor regions characterized by close apposition of glioma cells and neurons, defined by the co-occurrence of >20% tumor cells (SOX2⁺, OLIG2⁺, and/or SOX9⁺) and >20% NeuN⁺ neurons. In contrast, the frequency of pY876-GluA2^+^ cells was lower in glioma-enriched regions ( > 20% SOX2⁺, OLIG2⁺, and or SOX9⁺ with < 5% NeuN⁺) and in neuron-enriched regions ( > 20% NeuN⁺ with <5% SOX2⁺, OLIG2⁺, and SOX9⁺). These findings indicate that Y876-GluA2 phosphorylation is preferentially associated with microenvironments where glioma-neuron interactions occur, supporting a role for this signaling event in tumor-neural crosstalk (Fig. 5j-k). Collectively, our findings suggest that GBM cells increase the Y876 phosphorylation on Ca^2+^-permeable GluA2 to regulate tumor invasion at the GBM margin and at distant sites (Fig. 5l). Furthermore, the absence of GluA2 phosphorylation and GluA2(Q) expression in G12 cells suggests G12 cells are likely to engage a distinct signaling mechanism of dissemination from G6 cells.

### Cell cycle-associated signaling network is downregulated in disseminated GBM cells

**Fig. 6:**
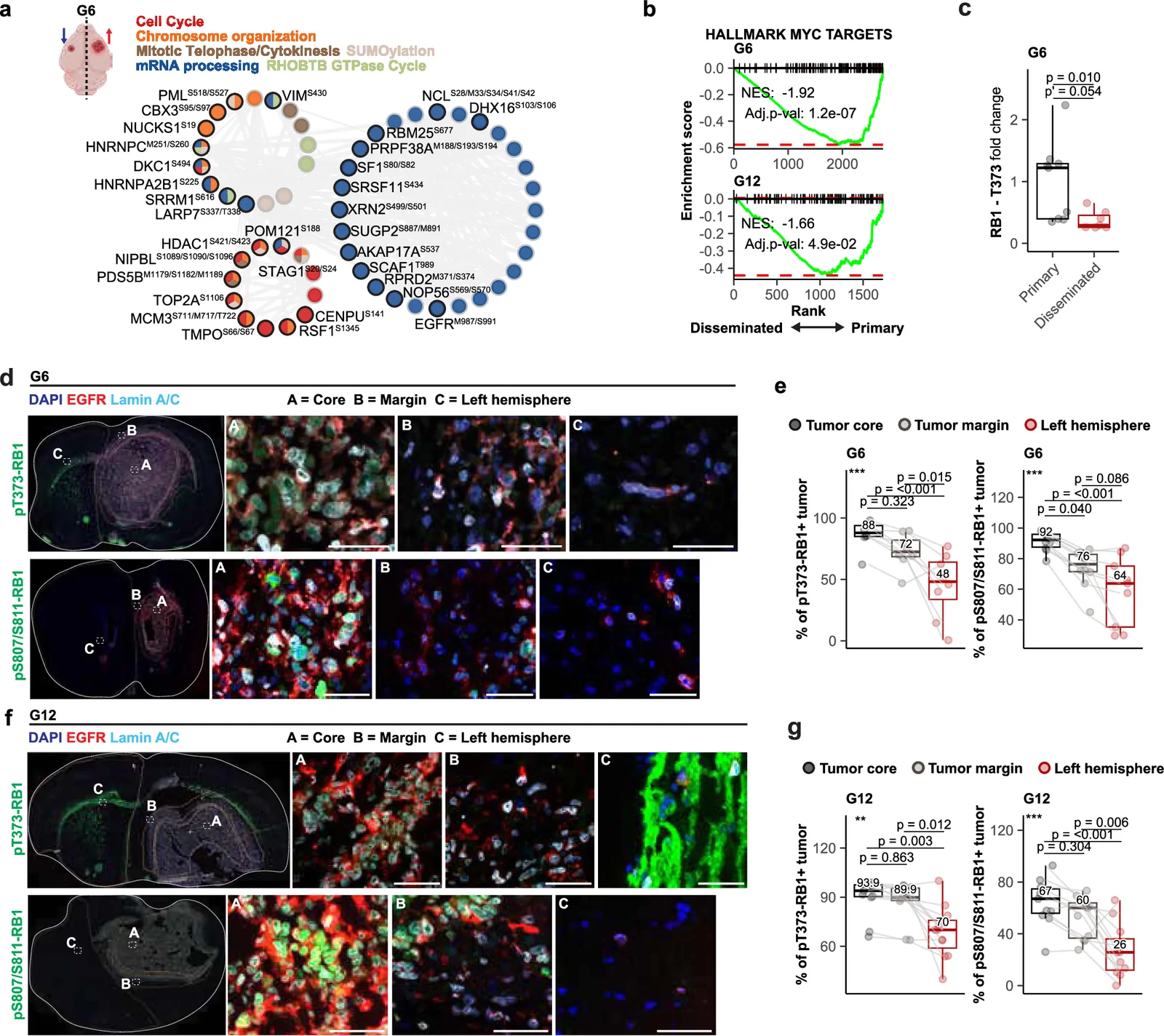
Proliferation-associated signaling network is suppressed in disseminated GBM cells. **a** Pathway enrichment analysis of phosphoproteins significantly downregulated in disseminated G6 tumors compared to primary G6 tumors. Membership of the phosphoproteins (nodes) in each pathway is denoted by the same colors as the pathway annotated (top). Pathway terms with the lowest P values were annotated, and those that shared the phosphoproteins and had similar pathway terms were shown as extra nodes. **b** GSEA plots for the gene set “Hallmark Myc targets v1” comparing disseminated vs. primary tumor cells. BH adjusted one-sided P value for each sample are indicated. **c** Fold changes of pT373-RB1 as quantified by INSIGHT of G6 tumor cells. BH-adjusted two-sided *P*-values (p’) and unadjusted two-sided *P*-value (p) shown. *n* = 9 primary and 7 disseminated biological replicates. **d** G6 PDX tumor-bearing brains analyzed by co-IF. Staining for pT373-RB1 (green), and pS807/S811-RB1 (green), with DAPI (blue, nuclei), human EGFR (red), and human Lamin A/C (cyan) shown. The whole section (left) with dotted lines delineates the left hemisphere, the core, and the invasive margin of the primary tumor. The magnified regions (right) shown are labeled in the whole section: A (tumor core), B (tumor margin), and C (left hemisphere). Scale bar = 40 µm. **e** The percentage of pT373-RB1+ and pS807/811-RB1 + G6 tumor cells stained in d. Biological replicates for pS807/811-RB1+ were contralateral hemisphere (*n* = 9), core (*n* = 10), and margin (*n* = 10), and for pT373-RB1+ were contralateral hemisphere, core, and margin (*n* = 8 each). Two-sided one-way ANOVA P values are shown at the top (left, *p* = 5.9 × 10⁻⁴; right, *p* = 2.9 × 10⁻⁴). **f** Same analysis as in (**d**) performed for G12 PDX model. **g** Same analysis as in **e** performed for G12 PDX model stained in (**f**). ANOVA P values: left, *p* = 1.8 × 10⁻³; right, *p* = 1.3 × 10⁻⁴. Source data are provided as a Source Data file. The box plots have center lines indicating the median, box limits indicating the upper and lower quartiles, and whiskers indicating 1.5× the interquartile range. For (**e**–**g**), Tukey’s multiple comparisons adjustment was done.

To identify signaling circuitries suppressed during tumor dissemination, we analyzed proteins with decreased phosphorylation in disseminated tumor cells compared to their primary counterparts. This analysis revealed that proliferation-associated signaling pathways were downregulated in G6 and G12 disseminated cells relative to the tumor core (Fig. 6a, Suppl. Fig. 10a). Moreover, MYC transcriptional targets, crucial for cell proliferation^71^, were downregulated in disseminated cells of both models compared to their primary counterparts by GSEA (Fig. 6b). These findings align with our GBM module enrichment result where primary tumor cells shifted from G1/S or G2/M cell states to other cell states upon dissemination (Fig. 4a). Notably, disseminated G6 tumor cells had reduced pT373-RB1 expression (undetected in G12) (Fig. 6c). RB1 phosphorylation at T373 by CDKs allows G1/S phase cell cycle progression and cell proliferation^72–74^. Indeed, by co-IF, the percentage of nuclear pT373-RB1-positive tumor cells was significantly decreased in the disseminated tumors relative to primary tumors in both G6 and G12 models (Fig. 6d–g, Suppl. Fig. 10b). Probing for pS807/811-RB1, which follows pT373-RB1 to secure G0 phase exit, similarly showed a marked decrease in disseminated tumor cells in G6 and G12 relative to their primary tumors^75,76^ (Fig. 6d–g, Suppl. Fig. 10c). Thus, our findings indicate that disseminating tumor cells undergo reduced cell cycling. These results support reports that tumor cells shift between proliferative and migratory states, decreasing proliferation to invade surrounding tissues^77,78^, and highlight that this transition is also reflected in their signaling networks at the systems level in vivo, which has not been previously recognized. Interestingly, we observed a smaller reduction of percent positive pS807/811-RB1, along with greater sustainment of pS807/8011 and pT373 levels in G6 compared to G12, indicating that G6 tumor cells likely continue to proliferate in the left hemisphere after dissemination (Fig. 6d–g). This aligns with the greater dissemination observed in G6 compared to G12 (Fig. 3a, Suppl. Fig. 3e, f). In addition, like pY876-GluA2 (Fig. 5e, f), pRB1 in tumor cells was reduced at the margin (Fig. 6e, g), indicating that tumor cells at the margin resemble those in the left hemisphere.

### Hornerin promotes GBM invasion and growth

**Fig. 7:**
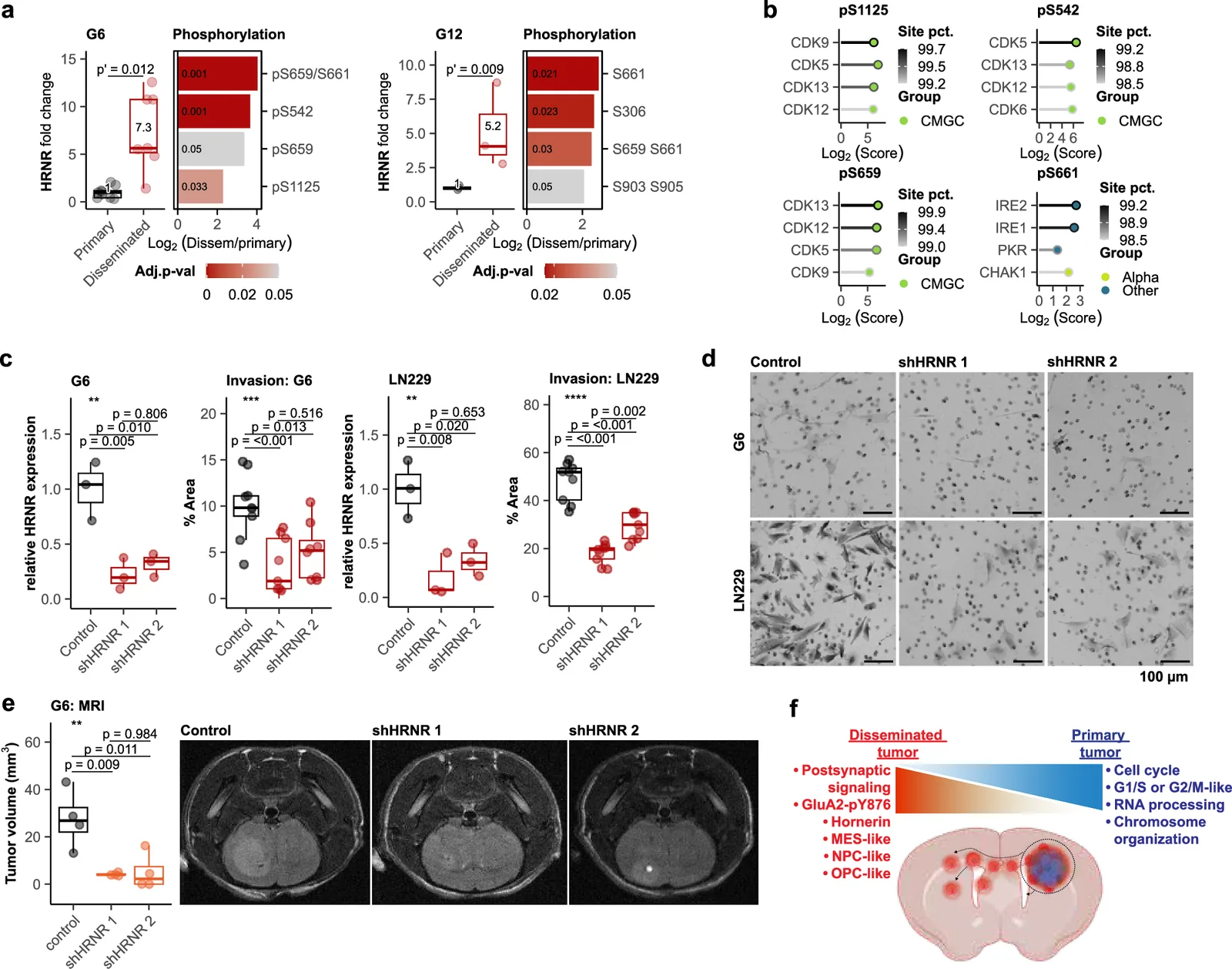
Hornerin promotes glioblastoma growth and invasion. **a** Fold change in the level of HRNR protein (Left) and phosphorylation at indicated residues (right) within disseminated (*n* = 7) and primary tumors (*n* = 9). The color intensity represents the adjusted P value. BH adjusted two-sided P values (p’) are shown. **b** Lollipop plots show the top kinases predicted by Kinase Library to phosphorylate each residue in (**a**), with their kinase groups indicated in the dot and the site percentile (pct.) score indicated by the color of the stick. **c** HRNR was knocked down in G6 and LN229 glioblastoma cells using two different shRNAs and the matrigel invasion assay was performed in vitro. One-way ANOVA *P*-values are indicated at the top (G6 HRNR level = 4.1e-03; G6 invasion = 7.2e-04; LN229 HRNR level = 7.08e-03; LN229 invasion = 1.3e-09). n = 3 technical replicates for expression analyses. For G6 invasion, *n* = 9 control, 9 shHRNR 1, and 8 shHRNR 2 technical replicates. For LN229, *n* = 9 control, 9 shHRNR 1, and 9 shHRNR 2 technical replicates. **d** Image of the invading cells in invasion chambers as assessed in (**c**). Scale bar = 100 µm. **e** G6 tumor cells knocked down with HRNR shRNA as shown in **c** were injected into Nu/Nu mice and MRI was performed. *N* = 4 biological replicates. **f** Disseminated (left hemisphere) and primary tumors (right hemisphere) as characterized by INSIGHT on GBM PDX models. Tumor cells (left, red) increased the engagement of synapse-associated signaling circuitries, GluA2 phosphorylation, and HRNR expression as they disseminated the normal brain. On the other hand, primary tumor cells (right, blue) engage in proliferation-associated signaling circuitries. Made using BioRender. Source data are provided as a Source Data file. The box plots have center lines indicating the median, box limits indicating the upper and lower quartiles, and whiskers indicating 1.5× the interquartile range. For (**c**, **e**), statistical significance was assessed using two-sided one-way ANOVA followed by Tukey’s multiple comparisons test.

In both PDX models, hornerin (HRNR), a calcium-binding S100 protein previously implicated in cell cycle regulation^79^ and invasive tumor progression through unknown mechanisms^80–84^ exhibited the highest upregulation during dissemination (Fig. 7a). Furthermore, CDKs implicated in neuronal migration, synaptic plasticity (CDK5)^85^ and transcriptional regulation (CDK12)^86^ were predicated as kinases for HRNR phosphorylation which were also elevated (Fig. 7b). Knocking down HRNR via shRNA significantly reduced the invasive capacity of G6 and LN229 GBM cells in vitro (Fig. 7c, d) and reduced the tumor growth of G6 in vivo (Fig. 7e). Together, our data implicate HRNR in promoting GBM invasion and growth and highlight its potential to participate in the cross-talk between neuronal, cell cycle, transcriptional regulation, and invasion-associated signaling circuitries.

## Discussion

We developed INSIGHT, a platform technology that enables discovery-based, systems-level signaling network characterization of specific cell types or subpopulations from complex tissues without requiring genetic engineering, positioning it as a valuable tool for both basic and translational research. The consistency between orthogonal experimental methods across key findings on GBM PDX models highlights the robustness of INSIGHT even for rare cell subsets. INSIGHT also offers opportunities for monitoring perturbations of kinases or phosphatases and their phosphorylated substrates specific to those cells, which could enhance our understanding of heterogeneous cellular functions and precision in therapeutic targeting in vivo. We highlighted several kinases that may exhibit high activity and could be ideal therapeutic targets in primary and disseminated GBM cells, including ALK2 in the former and MLK4 in the latter. Furthermore, inferring ligand-receptor interactions from signaling networks as we have done will enhance our understanding of cell-cell communication, as we can use phosphorylation status of receptors as a proxy for receptor activation, which cannot be gleaned from sequencing-based analysis. The development of computational tools that optimally leverage such data will be important. Moreover, INSIGHT overcomes various challenges faced by bulk phosphoproteomics of PDX models^36^, including distinguishing human and mouse proteins, highlighting its utility in phosphoproteomics of mixed-species models.

Using INSIGHT on preclinical, EGFR-driven GBM PDX models, we characterized thousands of phosphoproteins and proteins within disseminated GBM cells, providing new insights into the signaling networks underpinning GBM dissemination. Further investigation across a larger and more diverse cohort of EGFR driven and non-EGFR driven GBM xenograft models and patient specimens will be essential to define the broader mechanisms underlying GBM dissemination. INSIGHT provides a robust framework for systematically interrogating these processes in vivo. Nonetheless, our systems-level analysis of GBM signaling network rewiring in vivo with tumor spread showed a global transition from proliferative to neuronal program-associated signaling (Fig. 7f). We also highlight that signaling circuitries of neuronal and non-neuronal programs may potentially interconnect during GBM dissemination by predicting numerous phosphoproteins, proteins, and kinases to have relationships within those circuitries, which remain to be validated in the future. Alterations in phosphorylation rather than total protein expression were seen in many signaling components, underscoring the importance of quantifying post-translational modifications in vivo to fully understand mechanisms underlying disease progression. Importantly, our work points to a previously unrecognized role of pY876-GluA2 in GBM dissemination. In neurons, pY876-GluA2 mediates synaptic upscaling and long-term potentiation (LTP) by promoting the accumulation of GluA2 at the postsynaptic density^63–67,87,88^. Phosphorylation of Y876-GluA2(Q) may regulate neuron-GBM synaptic plasticity through similar mechanisms, particularly for those GBM cells with multi-synaptic contact with neurons^19^, facilitating tumor growth or invasion via neuronal interaction, while separately promoting invasion through neuron-independent but GluA2(Q)-dependent mechanisms that rely on increased GluA2 at the membrane. Further studies are needed to ascertain whether phosphorylation occurs on both GluA2(Q) and GluA2(R) and to define their functional relevance in GBM dissemination.

The relevance of interrogating the systems-level signaling networks within contralaterally disseminated GBM PDX cells for insights into invasive and recurrent GBM biology has been unclear. We clarify this by demonstrating that key phosphoproteins show consistent regulatory patterns in disseminated cells and those at the tumor margin—the dominant site of recurrence^14^. Disseminated cells also adopt a mesenchymal cell state and downregulate EGFR signaling, reflecting features of recurrent GBM^42–44,89,90^. Combining INSIGHT with microscopy-based validation therefore may offer a framework for investigating invasive GBM mechanisms contributing to recurrence. Furthermore, while temporal dynamics are not explicitly addressed here, INSIGHT provides a scalable framework for future studies aimed at reconstructing signaling trajectories across dissemination. Accordingly, this work defines a molecular baseline for the disseminated state that can be extended to incorporate temporal and therapeutic perturbations. Furthermore, the signaling programs enriched in disseminated GBM cells are consistent with phenotypes previously associated with relative resistance to cytotoxic therapies such as temozolomide, suggesting a testable framework for future studies interrogating treatment-induced state transitions using INSIGHT.

Several limitations of our study and of the INSIGHT platform remain, and addressing these through continued methodological refinement and further investigation are needed. First, fixation can negatively impact the epitope recognition of some antibodies. Antibodies targeting cytoplasmic markers and those used for immunohistochemistry will broaden the range of detectable markers. Second, we employed data-dependent acquisition, which, while effective for discovery-based proteomics, is inherently limited by stochastic precursor selection, impacting the reproducibility across experiments. Targeted acquisition strategies will increase sensitivity, reproducibility, and precision^91,92^. Third, dyes that rely on membrane integrity for live/dead cell discrimination become ineffective with fixation. While INSIGHT performed effectively in both normal and tumor-bearing tissues, strategies to discern live/dead cells without relying on membrane impermeability would minimize noise. Fourth, given that INSIGHT utilizes cryosections, sampling parallel tissue sections with spatial-omics technologies will increase the depth of biological insight. Concurrently, tissue sectioning likely results in underrepresentation of cell populations with extensive projections, owing to their increased susceptibility to structural disruption during processing. Fifth, as with all flow cytometric and microfluidic platforms, the identification and isolation of rare cell populations from large cellular backgrounds remains technically challenging, particularly when balancing processing speed, throughput, and feasibility with the goal of achieving near complete purity. Even with these current considerations, INSIGHT advances in vivo MS-based phosphoproteomics for targeted, cell subset-specific signaling network analysis, providing a platform to uncover mechanistic insights with broad applications in both basic and translational research. There is growing interest in enabling phosphoproteomic analyses at increasingly small spatial and cellular scales^93^. Collectively, these considerations place INSIGHT within a broader movement toward increasingly sensitive and spatially resolved phosphoproteomic analyses.

## Methods

### Animal studies

GBM6 (G6) and GBM12 (G12) PDX tumor intracranial inoculations were performed in female Nude mice as previously described^41,94^. Associated clinical information can be found in Supplementary Data 1. Maximal tumor burden, which is when mice reach a moribund state, was not exceeded. All PDX lines were established and maintained by the Mayo Clinic Brain Tumor PDX National Resource. Detailed methods related to PDXs are available online (https://www.mayo.edu/research/labs/translational-neuro-oncology/mayo-clinicbrain-tumor-patient-derived-xenograft-national-resource/protocols). G6 was maintained in FBS-based media, and G12 was maintained in stem cell media prior to injection. TdTomato-positive G6 PDX tumor cells were generated as previously described^95^. All animal work related to PDX-tumor generation was done in compliance with the Mayo Institutional Animal Care and Use Committee and Committee on Animal Care (CAC/IACUC) at MIT. Detailed information on the clinical and molecular characteristics of G6 and G12 PDXs is available on the Mayo Clinic Brain Tumor PDX National Resource maintained by the Sarkaria lab. For the studies using normal organs, male and female C57BL/6 mice were acquired from Taconic Biosciences, maintained, and necropsied in compliance with protocols approved by the Committee on Animal Care (CAC/IACUC) at MIT. The mice were housed on a 12/12 light cycle with a temp range of 70 + /− 2 and a humidity range of 30-70%. Mice ages ranging from 6 to 12 weeks were used.

### Magnetic resonance imaging

MRI was performed in vivo as previously described in ref. ^96^. Briefly, MRI was done using a 7 T Bruker AV4 NeoBioSpec70-20USR scanner equipped with a 114 mm, 660 mT/m actively shielded gradient and a QSN075/040 RF coil (Bruker BioSpin, Rheinstetten, Germany). Mice were anesthetized with 2.5% isoflurane by inhalation and maintained at 2–2.5% throughout imaging. Body temperature and respiration were monitored using a SAII monitoring and gating system (Small Animal Instruments Inc., Stony Brook). Anatomical brain images were acquired and reconstructed with Bruker Paravision PV360 v2.0. Axial T2-weighted images were obtained using TurboRARE (TR/TE = 3000/30 ms, averages = 4, RARE factor = 8), with a 256 × 256 matrix, 25 × 25 mm² field of view, 20 interleaved slices, 0.5 mm slice thickness with no gap. Images were converted to DICOM format and analyzed in ImageJ/Fiji(v1.54p). A polygon tool was used to delineate tumors for quantification in each slice. The summed area (mm2) was multiplied by 0.5 mm for the total tumor volume (mm3).

### Tissue culture

GBM6, LN229, and HEK293T cells were maintained in Dulbecco’s modified Eagle’s medium (Life Technologies) supplemented with 10% fetal bovine serum (Life Technologies). Cells were screened to be mycoplasma-free via MycoAlert Mycoplasma Detection Kit (Lonza). For in vitro assays, GBM6 cells were maintained in culture for less than two weeks on plates that were pre-coated with laminin (Sigma) at 1 ug/cm^2^.

### Patient biopsy

WHO grade IV glioblastoma patient tissue (fibrillary and gemistocytic astrocytoma) excised from right temporal lobe and preserved in O.C.T compound at the Mayo clinic was fixed and dissociated using INSIGHT and sorted using anti-CD15(HI98 clone)-BV786 (cat# 563838; Biolegend) and anti-CD84(Cd84.1.21 clone)-PE (cat# 326007; Biolegend) antibodies for mass spectrometry analysis. For MRI-guided multiregional biopsy of five GBM patients (2 tissue biopsies from 3-4 tumor locations; n = 20 specimens), all patients underwent pre-operative MRI to identify biopsy targets, and intra-operative image-guided Stealth neuro-navigation was used to define the image-based location of each sample. Each core biopsy was flash frozen. Patients were enrolled in the clinical trial (ClinicalTrials.gov identifier: NCT03071913) and tumor samples were obtained upon informed consent. All study procedures were performed and approved by the Mayo Clinic Institutional Review Board.

### Cyclic immunofluorescence (CyCIF)

Cyclic immunofluorescence (CyCIF)^13^ on fresh frozen core biopsy tissues was performed as previously described^97^. Briefly, fresh frozen patient glioblastoma samples (*n* = 20) were sectioned at 10μm using a Cryostat (Leica) and thaw-mounted on SuperfrostTM Plus microscope slides (Thermo). Mounted specimens were fixed with 4% paraformaldehyde/PBS, washed with PBS, permeabilized with 0.1% detergent solution (Thermo Scientific Cat. No. 28316) and blocked with SuperblockTM (Thermo). Specimens were stained using CyCIF, rounds of primary antibody staining and scanning followed by photobleaching to inactivate fluorophore signals before incubating with a next round of conjugated primary antibodies. Nuclear labeling was achieved with Hoechst 33342 (Cat. H3570) diluted 1:10,000. Phosphorylated GluA2 at tyrosine 876 was detected using a rabbit monoclonal antibody against GluA2 pY876 (Cat. 4027S, clone pY876; unconjugated) at a 1:100 dilution. Neuronal nuclei were labeled with a rabbit anti-NeuN antibody (Cat. ab190195, clone EPR12763) directly conjugated to Alexa Fluor 488 at 1:100. Other antibodies used were rabbit anti-OLIG2 (Cat. ab300730, clone EPR2673) conjugated to Alexa Fluor 555 at 1:100, rabbit anti-SOX2 (Cat. ab300548, clone EPR22952-70) conjugated to Alexa Fluor 555 at 1:100, and rabbit anti-SOX9 (Cat. ab196184, clone EPR14335) conjugated to Alexa Fluor 647 at 1:100. All primary antibodies were diluted in Hoechst 33342 and incubated overnight at 4oC in the dark. Specimens were photobleached in a solution of 4.5% H_2_O_2_, 24 mM NaOH in PBS for 1 hour under an LED light source to inactivate fluorophores. Scanning was performed on EVOS S1000 Spatial Imaging System (Thermo), stitching and registration of cycles were done in MCMICRO^98^ using ASHLAR^99^ modules. Additional details and code can be at found at www.cycif.org.

### Analyses for CyCIF

Multiplex cyclic immunofluorescence (CyCIF) datasets were analyzed using custom R scripts (v4.3 + ) to quantify spatial relationships between pGluA2-positive cells and tumor or neuronal cell populations. Single-cell segmentation outputs containing centroid coordinates and marker intensities were imported from tabular files and standardized by extracting cell identifiers and spatial coordinates. Datasets corresponding to pGluA2 and tumor–neuron marker panels were spatially registered using custom MATLAB functions. Tumor and neuronal cell identities were assigned using marker intensity thresholds (SOX9, SOX2, and OLIG2 for tumor; NeuN for neurons). Tumor enriched region ( > 40% SOX2 + , SOX9 + , OLIG2+ cells and 0% NeuN+ neurons), tumor-neuron interacting ( > 20% tumor cells and >40% neurons), and neuron enriched (0% tumor cells and > 40% neurons) per 50 μm squared were defined. Spatial analyses were performed by binning cell centroids into a regular grid (50 µm resolution) and computing per-grid counts of cell classes. Within each grid square, relative cellular composition was calculated, and regions were classified as neuron-enriched, tumor-enriched, or mixed based on predefined proportion thresholds. For each spatial region, pGluA2 abundance was quantified as total counts, mean counts per grid square, and composition-normalized abundance.

### Co-immunofluorescence microscopy and analysis

Frozen whole brains embedded in OCT compound were coronally sectioned at 5 µm, and Lamin A/C (Abcam) stain was performed at intervals to identify the location of the most prominent primary tumor and disseminated tumor to count the number and percentage of tumor cells in each region identified. Two sections from two different coronal depths were stained and analyzed per PDX tumor-bearing brain for 6 brains (total *n* = 12 sections). Tumor burden varied across depths. When disseminated tumor cells were absent, analysis of the primary tumor and margins was still performed. Sections lacking a clear midline to delineate the left hemisphere or those with extensive primary tumor growth that pushed into the left hemisphere were excluded, consistent with the INSIGHT analysis. The tumor core, margin, and left hemisphere were drawn manually for each section for analysis. Tissues were fixed with 2% paraformaldehyde/PBS, washed with PBS, permeabilized with 0.1% Triton100/PBS, and blocked with normal serum. Tissues were stained with primary antibodies targeting the pT373-RB1 (Abcam, cat# ab52975; 1:100. We note that pT373-RB1 antibody from Invitrogen did not work), pS807/811-RB1 (CST, cat# 8516S; 1:100), pY876-GluA2 (CST, cat# 4027S; 1:100), pT693-EGFR (CST, cat# 3056S; 1:100) and were probed with goat anti-rabbit IgG-AF488 (H + L) secondary antibody (Thermo). We note that strong staining in the corpus callosum was seen for the Abcam pT373-RB1 antibody. Sections were blocked with normal serum and incubated with rabbit anti-human Lamin A/C-AF594 (Abcam, cat# ab215324; 1:400) and mouse anti-human EGFR-APC (Biolegend, cat# 352906; 1:200). Sections were stained with DAPI after being subjected to TrueView (Vectors) to distinguish nuclei, mounted using Vectashield vibrance antifade mounting medium, and images were taken using a TissueFAXS SL slide scanner (TissueGnostics) coupled to a Zeiss upright microscope with a 20x or 40x objective with z-stack enabled. All images from TissueFAXS SL were analyzed using StrataQuest (v7.1.1.143). A nuclei mask was used for the quantification of pT373-RB1 and pS807/811-RB1 and Lamin A/C while a cellular mask was used for the quantification of EGFR and pT693-EGFR. For pY876-GluA2, the FISH dot algorithm was used. For all image analyses of disseminated tumors in the left hemisphere of the G12 PDX model, each tumor cell was visually validated. For confocal microscopy, Olympus FV1200 Laser Scanning Confocal Microscope was used at 60x objective with 2x zoom, and the images were analyzed using Fiji.

### Isolation of single cells from fixed tissue

Flash-frozen brains (normal or PDX-tumor bearing) embedded in OCT compound were cut down the mid-sagittal plane, and each hemisphere was sectioned at 50 µm using a Cryostat (Leica) and fixed for 40 min using Cytofast (Biolegend) (or other fixatives as noted during optimization). Fixatives tested were prepared or purchased as follows: 10% buffered formalin diluted from 20% buffered formalin that contains methanol stabilizer, 4% paraformaldehyde (PFA) diluted in PBS, 1.5% PFA diluted in PBS, commercial fixative Cytofast (Biolegend) used as 1X, Cytofix as 1X(BD Biosciences), Foxp3 fix/perm buffer as manufacturer protocol (Biolegend), and Phosflow lyse/fix buffer as manufacturer protocol (BD Biosciences). Normal brain and PDX tumor-bearing brain tissues were washed with RPMI (Corning) after fixation and incubated with 2 mg/ml Collagenase III (Stemcell) (or other enzymes as noted during method development) for a maximum of 30 minutes with periodic vortexing. For the enzyme test, collagenase I (Thermo), II (Thermo), and IV (Thermo) were tested. For permeabilization, Foxp3 perm buffer was used. Undissociated tissues were briefly sonicated for 5–15 sec in specific locations within a bath sonicator (VWR) to yield single-cell suspension. Dissociated cells were filtered using a 40 µm filter (VWR) and 30 µm filter (Miltenyi Biotec) and washed with 0.5% BSA/1 mM EDTA/PBS.

### Flow cytometry and sorting

All flow cytometric analyses were done using BD High Throughput Sampler (HTS) coupled to LSRFortessa (BD Biosciences). Anti-EGFR-PE (AY-13) antibody (Biolegend, cat# 352904; 1:200) was used to stain and identify tumor cells from normal cells for the samples subjected to mass spectrometry. For all sorting experiments, Sony MA900 (70 µm nozzle) was used to sort cells into 5 ml tubes. Cells were stained with anti-O4 antibody-APC (Miltenyi cat# 130-119-897; 1:100) to isolate oligodendroglial cells. For PDX tissues, normal mouse serum (Invitrogen), Human TruStain FcX Fc Receptor Blocking Solution and TruStain FcX™ (anti-mouse CD16/32) Antibody (Biolegend), and True Stain Monocyte Blocker (Biolegend) were added according to manufacturer instructions before staining cells for minimum 30 min. For testing fixatives, anti-EGFR-BV421 was used (cat# 352911; 1:200) with a gating strategy shown in Suppl. Fig. 11a. Mixture of normal and TdTomato+ tumor cells were stained with anti-HLA-A/B/C-APC-Cy7 [W6/32] antibody (Biolegend, cat# 311426; 1:2000) or anti-EGFR-BV421 antibody (Biolegend, Cat# 352911, 1:100-1:200) (gating strategy in Suppl. Fig. 11b). Anti-mouse pan cytokeratin antibody (AE1/AE3) – Alexa Fluor 488 (cat# 53-9003-82; Thermo; 1:200) was used to stain the 4T1 breast tumor cells. For dissociated PDX tumor-bearing brains, cells were first gated on FSC-A vs. SSC-A to exclude debris and aggregates, followed by FSC-W vs. FSC-H and SSC-H vs. SSC-A to isolate singlets, after which cells were gated on EGFR-PE vs. SSC-A to separate EGFR-positive and EGFR-negative populations (Suppl. Fig. 11c). Sorted cells were spun down to remove supernatant and pellets were stored at −80 °C before sample processing for mass spectrometry.

### Liquid chromatography-tandem mass spectrometry (LC-MS/MS)

For all PDX-related experiments (e.g., normal (EGFR-), bulk mixture of cells, tumor cells), a maximum of 1.5 million cells per sample was used. Three brains were analyzed by INSIGHT (processed, sorted, and analyzed by MS) at a time. All fixed samples were lysed in 50 mM Tris/5%SDS (pH 8.5), heated at 80 °C, sonicated, reduced with 10 mM DTT, alkylated with 55 mM IAA, and bound to s-trap (Protifi), washed with 50% chloroform/50% methanol, washed with 100 mM TEAB/90% methanol, and digested with trypsin overnight before elution. After briefly drying down in a vacuum centrifuge, samples were then lyophilized. For TMT labeling, lyophilized peptides were resuspended in 50 mM HEPES, combined with TMT (Thermo), and incubated for 2 h before being quenched with hydroxylamine and pooled. Labeled samples were dried down in a vacuum centrifuge and lyophilized overnight. For tyrosine phosphoproteome, the enrichment of pY peptides was performed by resuspending the TMT labeled samples in immunoprecipitation buffer (100 mM Tris/1% NP-40) and incubating digested peptides with 60 μl of protein G agarose beads (Sigma) pre-conjugated to 24 μg of 4G10 (Bioxcell) and 6 ul of PT66 (Thermo) antibodies overnight. Subsequently, immunoprecipitated peptides were eluted with trifluoroacetic acid and enriched for phosphopeptides using ferric nitrilotriacetate (Fe-NTA) columns (Thermo). Eluates from the Fe-NTA columns were dried using a vacuum centrifuge, reconstituted in 3% acetonitrile/0.1% formic acid, and manually loaded using a column-packing device without introducing air onto a 12 cm analytical column containing 3 μm C18 beads freshly hand-packed and conditioned on the day of each experiment. Analytical columns were laser-pulled, fritted with Lithisil and formamide, and flamed to open the frits on the day of each experiment. Only a freshly packed column was used for each pY run or fractions. Agilent 1100 Series HPLC connected to Orbitrap Exploris 480 Mass Spectrometer was operated at 0.2 ml/min flow rates with a precolumn split to attain nanoliter flow rates through the analytical column and nano-electrospray ionization tip. Peptides were eluted with the increasing concentrations of buffer B using the following 140-minute gradient settings (Buffer A: 0.1% acetic acid; Buffer B: 70% acetonitrile/0.1% acetic acid): 0 min: 0% B; 10 min: 11% B; 110 min: 32% B; 125 min: 60% B; 130 min: 100% B; 128 min: 100% B; 130 min: 0% B. The MS parameters were: ESI spray voltage, 2.5 kV; no sheath or auxiliary gas flow; heated capillary temperature, 275 °C, data-dependent acquisition mode with the scan range of 380–2000 m/z. MS1 scans were acquired at 60,000 resolution, maximum injection time of 100 ms, normalized AGC target of 300%, and only included the precursor charge states of ≥ 2 and ≤ 6. For every full scan, MS/MS spectra were collected during a 3-second cycle time. For MS/MS, ions were isolated (0.4 m/z isolation width) with the the maximum IT of 250 ms, normalized AGC target of 100%, HCD collision energy of 33% at a resolution of 60,000, and the dynamic exclusion time of 35 seconds. Half of the supernatant from pY-IP was subjected to high-pH reverse-phase fractionation on a Kromasil® C18 HPLC Column (5 μm particle size, pore size 100 Å, L × ID 250 mm × 4.6 mm) using buffer A (10 mM triethylammonium bicarbonate (TEAB), pH 8) and buffer B (10 mM TEAB, pH 8, 99% acetonitrile) over an 85-minute gradient (0 min: 1% B, 1 min: 1% B, 5 min: 5% B, 65 min: 40% B, 75 min: 70% B, 84 min: 70% B, 85 min: 1%) into 10 fractions (concatenated) using a Gilson FC 204 Fraction Collector and Agilent 1100 Series HPLC. Fractionation was performed at a flow rate of 1 ml/min and 1 min per fraction between 10-85 min portion of the gradient. For each fraction, 10% was allocated for protein expression analysis, and 90% was allocated for global phosphoproteomic (pS/pT and residual pY not already captured by 4G10 and PT66 antibodies) analysis. For simplicity, data generated from the global phosphoproteomic analysis were denoted as “pS/pT” while those from pY-IP-enrichment were denoted as “pY” in the study. Fractions for global phosphoproteome analysis were resuspended in 0.2% TFA and subjected to Fe-NTA column-based phosphopeptide enrichment, after which they were eluted, dried, and resuspended in 5% acetonitrile/0.1% formic acid for injection by Dionex UltiMate 3000 Autosampler (Thermo) on a 1.9 μm C18 bead-packed analytical columns.

### Mass spectrometry data analysis

Mass spectra were searched using Mascot (v2.8) and Proteome Discoverer (v3.0) against the Swiss-Prot database (*Mus musculus* proteome for murine tissues, and concatenated *Homo sapiens* and *Mus musculus* proteome (2023_06 and 2024_03) appended with common Repository of Adventitious Proteins (cRAP) fasta sequences for PDX or mixed tissues). Raw files were searched with two or fewer missed cleavages, precursor and fragment ion matched with 10 ppm and 20mmu mass tolerances, and fixed modifications of carbamidomethyl (Cys), TMT-pro ( + 304.2071) at peptide N-terminus and lysine and dynamic modification of oxidation (Met). For phosphoproteomic data, additional dynamic modifications of phosphorylation (Ser, Thr, Tyr) were added. TMT reporter ion intensities were corrected with isotope correction factors provided by Thermo Fisher (lot # for each run can be found on PRIDE). A target-decoy search strategy was used to adjust Peptide Spectral Matches (PSMs) to 1% FDR, and the Percolator^100^ node was used in the processing step to improve the sensitivity and accuracy of peptide identification and the resulting q-values were used to keep only those PSMs with q-value ≤ 0.01 (“1% FDR” reported in figures). The ptmRS^101^ node was used to confidently assign the phosphorylated and oxidized sites (filtered for peptides with localization probability ≥ 90% after search). PSMs were further filtered with ion scores ≥20, a cutoff determined in-house through extensive manual validation of spectra for higher-stringency identification (“HS”) compared to 1% FDR^27^. TMT intensities for common PSMs were summed for peptide identification and quantification. Proteins mapped to only one protein accession per species were used for all analyses. For protein accessions without associated gene names, protein accession itself was used for graphing. The residue positions of the phosphorylation (Y, S, T) and oxidation (M) were extracted using the full-length sequence of the longest isoform of the protein. To adjust for differential sample loading, all protein expression and phosphoproteomic data were normalized first using the median intensity of each TMT channel from their matching protein expression data. To join all the multiplexed sets derived from the PDXs, the samples within each plex were normalized to the normal cells from the left hemisphere with the assumption that those cells stay relatively consistent across all brains. For the analysis of O4 positive cells, primary tumors, and normal cells from the left hemisphere, all missing values were filled in with the lowest TMT intensity from the corresponding spectrum that was extracted and divided 2-fold and used to impute the missing values (“MGF method”) as previously described in ref. ^102^ with the assumption that the majority of these values were not detected because they were absent or low in abundance (e.g., O4-positive cells may not express proteins that O4-negative cells express, and vice versa). If the lowest m/z intensity was larger than the lowest isotope impurity-corrected TMT reporter intensity within the PSM, ½ of the latter was used instead. To do this, MGF (Mascot Generic Format) files were read in with the MSnbase (2.28.1)^103^, and the intensities were assigned based on the First Scan number. For the analysis of disseminated tumor cells, all PSMs with missing values across all samples were removed from the analysis (with the assumption that most of the missing value arose due to low sample input and not because they did not express those peptides), except for those PSMs where missing values existed only in samples other than the disseminated tumor samples (here the missing values were replaced using the MGF method as above). This was done to avoid bias introduced by imputation. This allowed the plotting of the relative changes of tumor-specific markers (human lamin A/C (LMNA), human OLIG2, human Nestin (NES), human EGFR) across all samples. To retain maximum information without imputing or excluding phosphopeptide or protein identifications (IDs) with missing values, IDs with equal combinations of sample sizes per condition were grouped and subjected to differential expression analysis. To remove potential doublets and debris that may be of murine origin for isolated tumor cells, the following criteria were used to filter the protein expression data for each multiplexed set: 1) when proteins arose from peptides that uniquely mapped to human (ph) or both human and mouse (phm) proteomes, only ph-derived proteins and their TMT intensities were kept; 2) when the same protein arose from both phm and pm, Pearson correlation analysis across all samples across plexes was done to remove the phm intensities that had a correlation coefficient (*r*) > 0 to pm regardless of the adjusted P value, aside for those with 0 ≤ *r* ≤ 0.2 and adjusted P value > 0.3; 3) peptides that uniquely mapped to mouse proteome (pm) were removed. For the phosphoproteome data, all values associated with phosphopeptides whose total proteins in the protein expression data were filtered out using the aforementioned criteria were also removed. Furthermore, phosphopeptides that mapped to both human and mouse proteins were removed if the protein expression data only detected the murine version of its matching total protein. Phosphopeptides that were present in less than three disseminated tumor samples and three primary tumor samples were removed from consideration. EGFR-positive tumor populations from two left hemispheres (A and F) were excluded from data analysis, as they represented portions of the primary tumors that extended into the left hemisphere and inadvertently included during the mid-sagittal dissection, resulting in their high percentage of tumor cells compared to other left hemispheres during sorting (Suppl. Fig. 3g). The same phenomenon was observed in the batches of tumor-bearing brains analyzed by microscopy, and these cases were excluded from analysis as detailed in the microscopy methods section.

### RNAseq

Previously published bulk RNAseq data^31^ for intracranially implanted core G6 and G12 PDX tumors were retrieved and re-processed. Data processing was done in part using Nextflow-based nf-core workflows^104^. RNA-seq data from bioproject PRJNA548556 with sample IDs SRR9294059 (G6) and SRR9294060 (G12) were obtained using the nf-core/fetchngs version 1.12.0^105^ and used to quantify transcripts from a combined human hg38 and mouse mm39 genome assembly with Ensembl version 112 annotations using the nf-core/rnaseq workflow revision 3.14.0^106^. Gene level summaries were prepared from the star_salmon quantitation using tximport version 1.32.0^107^ running under R version 4.4.1^108^ with tidyverse version 2.0.0^109^. The R image used for this work is available (docker://bumproo/bulk_r441). Genomic alignments were visualized using the Integrated Genomics Viewer version 2.17.0^110^. Human-specific protein-coding gene expression values were reported as log_2_-transformed Transcripts Per Million (TPM) with a + 1 offset in all figures and tables (Suppl. Data 3). For the analysis of amino acid 607 codon sequence coverage in human *GRIA2* and mouse *Gria2*, reported percentages included all aligned reads.

### Generation of HRNR knockdown cells

Control and human *HRNR*-silenced cells were generated using MISSION® pLKO.1-puro-CMV-TurboGFP lentiviral plasmids (Sigma). The control vector (SHC003) and shRNAs targeting *HRNR* (HRNR1 clone ID TRCN0000159629; HRNR2 clone ID TRCN0000159662) were used. Lentiviral particles were produced in HEK293T cells using polyethylenimine. Transduction of all cells was performed for 24 h (polybrene added at 8 ug/ml) and polyclonal cells were selected with puromycin (2 ug/ml) and visually confirmed to express GFP. Quantitative PCR was performed to confirm knockdown using the following primers: HRNR Forward 5’ CAGCTATGGTGAGCAAAACTCC 3’ and Reverse 5’ GACTTGTGTTGACTAAAGCCAGA 3’; GAPDH Forward 5’CCACTCCTCCACCTTTGACG3’, Reverse 5’CCACCACCCTGTTGCTGTAG3’; TBP Forward 5’GGTTTGCTGCGGTAATCA3’ and Reverse 5’TGTTCTTCACTCTTGGCTCCTGT3’.

### Generation and verification of GluA2 plasmids

Human *GRIA2*/GluA2 (NM_001083619) plasmid was purchased from Origene (Cat# RC212599). Mutations at R607Q and Y876F were performed using Q5 Site-Directed Mutagenesis Kit (cat# E0554S, NEB) and primers ordered from IDT and Eton Bioscience. pENTR/D-TOPO cloning kit (cat# K240020, Thermo) was used to create pENTR plasmids and LR reaction was carried out using Gateway™ LR Clonase™ II Enzyme mix (cat# 11791020, Thermo) and pInducer20-Blast-HA-tag containing plasmid (#109334 Addgene). Sanger sequencing of the plasmids were performed at Genome Quebec. For the intracranial injection of G6 PDX expressing GluA2 wild-type and mutants tagged with V5, the same Gateway entry clones (pENTR vectors) were recombined into the lentiviral destination vector pLX_307 (Broad Institute) using an LR reaction and verified by sequencing and western blot.

### Immunoblot

To verify the proper expression of GluA2 wild-type and mutants, cells were lysed (lysed in phsopholipase-C-γ lysis buffer^111^. Lysates were analysed by immunoblot analysis using the following antibodies: HA-Tag (C29F4) Rabbit mAb (#3724, CST) and alpha Tubulin Monoclonal Antibody (DM1A) (cat#62204, Thermo). The blots were incubated with horseradish peroxidase-conjugated secondary antibodies (Jackson Laboratories) and visualized by Pierce™ ECL Western Blotting Substrate (cat#32106, Thermo).

### Invasion and migration assay

BioCoat Matrigel Invasion Chambers with 8.0 µm PET Membranes (cat# 354480 Corning or #62405-744 VWR) were used to assess the invasion capacity of cells according to manufacturer protocol. Minimum of three wells per experimental condition were used. Images were taken using a Zeiss Axio Observer inverted microscope (10x objective) coupled with an Axiocam 506 monochrome CCD camera. Three or four fields were taken per well and analyzed using Fiji. Stacks were converted to a binary image (automation method = “Huang”, background = “light”) and the Analyze Particle option (size = 10-infinity; pixel units) was used. The percentage of area plotted represents the percentage of positive pixel counts of the cells over the total area analyzed. A well without cells was used as a control to subtract the positive pixel counts from the pores (equally present in all conditions). Migration assay was performed on a 96 well plate (*n* = 4 per condition) using Incucyte and Incucyte wound maker. For GluA2 plasmid expressing cells, cells were first selected with Blasticidin S for three days and treated with doxycycline (100 ng/ml final) for 24 h prior to the assessment of invasion and migration capacity of cells.

### Data analysis

All data were processed and analyzed using R (v4.3.1) and R studio. Differential expression analysis was performed using the limma R package^112^ with a significance cutoff of adjusted *P*-value ≤ 0.05. A minimum of *n* = 3 data points per condition was required for each phosphopeptide and protein to be assessed for differential expression and statistical significance by limma and for downstream analyses. To retain maximum information without imputation or excluding IDs with any missing values, IDs with equal sample sizes per condition were grouped and subjected to differential expression analysis and multiple testing correction. For stemness signature enrichment analysis, the list of genes were curated from the literature^113–117^. ClusterProfiler^118^ v4.8.3 (with OrgDb from March 2023) and EnrichR^119^ (v3.2) were used for gene ontology term enrichment analysis, with P values adjusted by the Benjamini-Hochberg procedure. Redundant terms were removed using the simplify function in ClusterProfiler. Custom backgrounds were made using the list of proteins in each mass spectrometry data from which the enriched IDs were extracted. For GO Biological Process, Molecular Function, and Cellular Component databases, v2023 was used. Oligodendrocyte-specific markers were retrieved from PanglaoDB^120^ and Tabula Muris^121^, and markers that had evidence in the literature to be expressed highly in other cell types of the brain from these datasets were filtered out. GSEA on protein expression data was done using the fgsea R package^122^, with the minimum size of the signature set at 20. For GO term enrichment analyses of significantly differentially expressed IDs (FC ≥ 1.5 and adj. *p*-val ≤ 0.05) and GSEA, all phosphoproteins and proteins that were detected at least three times across conditions being tested were used. Heatmaps represent only the IDs that had no missing values across all samples of given conditions presented. To assess the subcellular localization of proteins that are altered under various experimental conditions, subcellular localization of each protein was retrieved from Uniprot and collapsed into high-level GO cellular component parent categories. PLS-DA was performed using the mixOmics R package. Genes with VIP scores above +/− log_2_(1.7) were considered relevant and were used for pathway enrichment analysis. In Supplementary Data 2, the Altered.by.fixation column denotes whether proteins and phosphopeptides were altered or unaltered by fixation, as determined by comparison of fixed and unfixed mouse brain control samples. Proteins and phosphopeptides detected exclusively in the xenograft experiments were annotated as unknown.

The visualization of the kinome and phosphatome was done using Coral^123^ and CoralP^124^ in R. Only murine kinases and phosphatases with corresponding human homologs were included in the plot (kinases and phosphatases that only exist in mice and not human were not detected in the experiment). The visualization of STRING networks^125^ and the Enrichment analysis (ClueGo 2.5.9^126^) on signaling networks were done in Cytoscape (3.9.1)^127^ using Biological processes (2022.05.25) and WikiPathways (2022.02.23) databases. GO term with the lowest term P value within the ontology group was shown. NicheNetR^28^ was used to evaluate ligand-receptor relationships between O4-negative (sender) and O4-positive cells (receiver) cells, aiming to identify those potentially regulating myelination in O4-positive cells. A signature associated with myelin maintenance was retrieved from MSigDB. O4-positive cells cell-specific proteins were defined as proteins that had log_2_(O4-positive cells/O4-negative cells) greater than 0 (adj. *p*-val ≥ 0.25), and O4-negative cell-specific proteins were defined as proteins that had log_2_(O4-positive cells/O4-negative cells) less than 0 in the protein expression data. All quantified phosphorylation sites by INSIGHT on receptors expressed by O4-positive cells were shown, irrespective of whether phosphorylation levels were higher in O4-positive or O4-negative cells. Thus, in cases where the receptors were more abundant in O4-positive cells but exhibited higher phosphorylation in O4-negative cells, suggestive of shared protein expression between the two populations, the phosphorylation levels were represented as negative values in the heatmap. The predictive power of each ligand in driving oligodendrocyte differentiation protein expression in the O4-positive cells relative to background protein expression was assessed to find ligands with the highest regulatory potential using NicheNetR. Receptors expressed in O4-positive cells that could bind the prioritized ligands from O4-negative cells and whose phosphorylation sites were mapped were then plotted.

To calculate the GBM module enrichment scores on protein expression data, the genes specific to each GBM module (neural-progenitor-like (NPC), oligodendrocyte-progenitor-like (OPC), astrocyte-like (AC), hypoxia-independent mesenchymal-like (MES1), hypoxia-dependent mesenchymal-like (MES2), G1/S, and G2/M cell cycle states) were retrieved from Neftel et al.^46^. Then, only the corresponding proteins that were detected and quantified by INSIGHT across all samples were used to calculate the enrichment scores using the single sample GSEA (ssGSEA) function from the GSVA package^128^. For the Sankey plot, the module that had the highest enrichment score in each tumor sample was plotted using networkD3.

To assess the synaptic proteins and phosphoproteins expressed by G6 and G12 PDX tumor cells, the SynGO annotations (version 20231201) were accessed and downloaded through the SynGO website^129^. For bulk RNAseq data, only the genes with log_2_(TPM + 1) ≥ 0.1 were considered for SynGo analysis. To assess the overlap between the genes associated with metastasis, migration, invasion, epithelial-to-mesenchymal or mesenchymal-to-epithelial transition (EMT/MET), and synapse, lists of gene sets were curated from MSigDB using the msigdbr package (species = Homo Sapiens) and manually verified and curated using keywords (e.g., invasion), except for synapse-associated genes, which were downloaded from SynGO^129^.

To infer active kinases in the primary tumors, the pY, pS, and pT motifs (7 amino acid residues flanking the phosphorylated residues) were extracted from peptides that mapped to human proteome and analyzed through the Kinase Library^4,5^ with predetermined foreground ( ≥ 1.5-fold change, adj. *p*-val ≤ 0.1) and background sets (the rest of the phosphopeptide motifs that mapped to human proteome in the same experiment). For the inference of kinases that mediate the phosphorylation of specific residue sites, the Score Multiple Sites tab on the Kinase Library website was used. For the differential kinase activity of primary and disseminated tumor cells, phosphoproteomic enrichment analysis option was used with fold change threshold of 1.5x, *P*-value threshold of 0.05, score percentile threshold of 95%. For each kinase, only substrates ranked 1 (i.e., the highest-confidence predicted substrates based on percentile rank) were included in the enrichment analysis. For all analyses, non-canonical kinases were included. Kinase activity was inferred using an independent substrate-based enrichment analysis leveraging curated kinase-substrate relationships from PhosphoSitePlus (database from 2021). Regulatory site information on kinases quantified were assessed using SIGNOR (2025) and PhosphoSitePlus (2024). Site-resolved phosphoproteomic measurements were first mapped to reported kinase substrates based on UniProt accession and modified residue position. Phosphosites detected in the dataset were ranked by log2 fold-change between disseminated and primary tumor cells, and kinases were tested for coordinated shifts in phosphorylation across their reported substrates using rank-based gene set enrichment analysis (fgsea). Normalized enrichment scores (NES) and false discovery rate–adjusted P values were computed for each kinase.

Various R packages for data processing, visualization, and plotting included paletteer (1.6.0), patchwork^130^ (1.3.0), magrittr (2.0.3), reshape2 (1.4.4), ggplotify (0.1.2), matrixStats (1.4.1), ggrepel (0.9.6), ggpubr (0.6.0), qdap (2.4.6), RColorBrewer (1.1-3), qdapTools (1.3.7), qdapRegex (0.7.8), qdapDictionaries (1.0.7), rlang (1.1.4), plyr (1.8.9), readxl (1.4.3), colorRamp2 (0.1.0), stringi (1.8.4), shadowtext (0.1.4), data.table (1.16.2), stringr (1.5.1), dplyr (1.1.4), purrr (1.0.2), readr (2.1.5), tidyr (1.3.1), tibble (3.2.1), tidyverse (2.0.0), ggplot2 (3.5.1), and ComplexHeatmap^131^ (2.18.0). complexupset^132^ (1.3.3), ggVennDiagram^133^ (1.3.0). Schematics were created in https://BioRender.com and Adobe Illustrator.

### Statistics and reproducibility

All statistical analyses were done using R (v4.3.1) in R studio. The statistical significance in plots of proteins and phosphopeptides identified by mass spectrometry are FDR-adjusted two-sided P values (p’). Non-adjusted P values were denoted as two-sided p only in the plots where quantifications were derived from LC-MS/MS. For co-immunofluorescence images, one-way ANOVA was used to test whether there were any overall differences among the cells in the core, margin, and left hemispheres, followed by Tukey’s Honest Significant Difference (HSD) test to perform two-sided pairwise comparisons between conditions and control the family-wise error rate. Plots show adjusted two-sided *P*-values between conditions, and asterisks in the top left corner represent the P values from ANOVA (**** = *P*-value ≤ 0.0001, *** = *P*-value ≤ 0.001; ** = *P*-value ≤ 0.01; * = *P*-value ≤ 0.05, ns = *P*-value > 0.05). All box plots show the median, interquartile range (IQR, 25th to 75th percentile), and whiskers (up to 1.5 × IQR).

### Reporting summary

Further information on research design is available in the Nature Portfolio Reporting Summary linked to this article.

## Supplementary information

Supplementary Information

Description of Additional Supplementary Files

Supplementary Data 1

Supplementary Data 2

Supplementary Data 3

Supplementary Data 4

Supplementary Data 5

Supplementary Data 6

Supplementary Data 7

Reporting Summary

Transparent Peer Review file

## Source data

Source data

## Data Availability

The mass spectrometry proteomics data have been deposited to the ProteomeXchange Consortium via the PRIDE [1] partner repository with the dataset identifier PXD056965 and https://www.ebi.ac.uk/pride/archive/projects/PXD056965/public. GBM6 (SRR9294059) and GBM12 (SRR9294060) RNAseq raw data can be obtained from https://www.ncbi.nlm.nih.gov/sra/?term=SRR9294059 and https://www.ncbi.nlm.nih.gov/sra/?term=SRR9294060. Source data are provided with this paper.

## Code availability

Codes used in this study are available at https://github.com/ryuhjinahn. The R image used for the RNA-seq data analysis is also available (docker URI: bumproo/bulk_r441; link: https://hub.docker.com/r/bumproo/bulk_r441).
