# Author Correction: Uncovering the signaling networks of disseminated glioblastoma cells in vivo with INSIGHT

**DOI:** 10.1038/s41467-026-78236-y

**Published:** 2026-09-29

**Authors:** Ryuhjin Ahn, Alicia D. D’Souza, Lawrence Long, Yufei Cui, Jennifer Gantchev, Gerard Baquer, Danielle Burgenske, Katrina K. Bakken, Lauren L. Ott, Brett L. Carlson, Grace Zhou, Ishwar N. Kohale, Charles A. Whittaker, Heidi Temple, Tomer M. Yaron-Barir, Jeffrey Wyckoff, Terry C. Burns, Rachael A. Vaubel, Cameron T. Flower, Wei Huang, Ann Tuma, Jared L. Johnson, Nathalie Y. R. Agar, Josie Ursini-Siegel, Jann N Sarkaria, Forest M. White

**Affiliations:** 1 David H. Koch Institute for Integrative Cancer Research, Massachusetts Institute of Technology (MIT), Cambridge, MA USA; 2 Department of Biological Engineering, MIT, Cambridge, MA USA; 3 MIT-Harvard Health Sciences and Technology, Cambridge, MA USA; 4 Department of Neurosurgery, Brigham and Women’s Hospital, Harvard Medical School, Boston, MA USA; 5 Department of Electronic Engineering, Rovira i Virgili University, Tarragona, Spain; 6 Mayo Clinic, Rochester, MN USA; 7 MIT, Cambridge, MA USA; 8 Barbara K. Ostrom (1978) Bioinformatics and Computing Core Facility of the Swanson Biotechnology Center, David H. Koch Institute for Integrative Cancer Research, Cambridge, USA; 9 Columbia University Vagelos College of Physicians and Surgeons, New York, NY USA; 10 Microscopy Core Facility of the Swanson Biotechnology Center, David H. Koch Institute for Integrative Cancer Research, Cambridge, USA; 11 Program in Computational and Systems Biology, MIT, Cambridge, MA USA; 12 Preclinical Imaging and Testing Facility of the Swanson Biotechnology Center, David H. Koch Institute for Integrative Cancer Research, MIT, Cambridge, MA USA; 13 Department of Cell Biology, Harvard Medical School, Boston, MA USA; 14 Dana-Farber Cancer Institute, Harvard Medical School, Boston, MA USA; 15 Department of Radiology, Brigham and Women’s Hospital, Harvard Medical School, Boston, MA USA; 16 Department of Cancer Biology, Dana-Farber Cancer Institute, Boston, MA USA; 17 Division of Experimental Medicine, McGill University, Montreal, QC Canada; 18 Lady Davis Institute for Medical Research, Jewish General Hospital, Montreal, QC Canada

**Keywords:** Metastasis, Cancer microenvironment

Correction to: *Nature Communications* https://doi.org/10.1038/s41467-026-76587-0, published online 8 August 2026

In this article the authors name Jann N Sarkaria was incorrectly written as Sarkaria Jann. The original article has been corrected.

