# Supplementary material for: Uncovering the signaling networks of disseminated glioblastoma cells in vivo with INSIGHT
Source: Nat Commun. 2026 Aug 8;17:9536. doi: 10.1038/s41467-026-76587-0 (PMC13547291; doi:10.1038/s41467-026-76587-0)
Supplement: Supplementary file 1 [file 41467_2026_76587_MOESM10_ESM.pdf]

nature portfolio

Corresponding author(s): Forest M. White
Ryuhjin Ahn

Last updated by author(s): Feb 18, 2026

Reporting Summary

Nature Portfolio wishes to improve the reproducibility of the work that we publish. This form provides structure for consistency and transparency in reporting. For further information on Nature Portfolio policies, see our Editorial Policies and the Editorial Policy Checklist.

Statistics

For all statistical analyses, confirm that the following items are present in the figure legend, table legend, main text, or Methods section.

n/a	Confirmed	
☐	☒	The exact sample size (n) for each experimental group/condition, given as a discrete number and unit of measurement
☐	☒	A statement on whether measurements were taken from distinct samples or whether the same sample was measured repeatedly
☐	☒	The statistical test(s) used AND whether they are one- or two-sided<br>*Only common tests should be described solely by name; describe more complex techniques in the Methods section.*
☐	☒	A description of all covariates tested
☐	☒	A description of any assumptions or corrections, such as tests of normality and adjustment for multiple comparisons
☐	☒	A full description of the statistical parameters including central tendency (e.g. means) or other basic estimates (e.g. regression coefficient) AND variation (e.g. standard deviation) or associated estimates of uncertainty (e.g. confidence intervals)
☐	☒	For null hypothesis testing, the test statistic (e.g. F, t, r) with confidence intervals, effect sizes, degrees of freedom and P value noted<br>*Give P values as exact values whenever suitable.*
☒	☐	For Bayesian analysis, information on the choice of priors and Markov chain Monte Carlo settings
☐	☒	For hierarchical and complex designs, identification of the appropriate level for tests and full reporting of outcomes
☐	☒	Estimates of effect sizes (e.g. Cohen's d, Pearson's r), indicating how they were calculated

Our web collection on statistics for biologists contains articles on many of the points above.

Software and code

Policy information about availability of computer code

Data collection	Thermo Scientific XCalibur (commercial license) for mass spectrometry data collection.
Data analysis	Data analysis was done using R (4.3.1) and R studio (2023.06.0). R packages and softwares used for data processing, visualization, and plotting included paletteer (1.6.0), patchwork121 (1.3.0), magrittr (2.0.3), reshape2 (1.4.4), ggplotify (0.1.2), matrixStats (1.4.1), ggrepel (0.9.6), ggpubr (0.6.0), qdap (2.4.6), RColorBrewer (1.1-3), qdapTools (1.3.7), qdapRegex (0.7.8), qdapDictionaries (1.0.7), rlang (1.1.4), plyr (1.8.9), readxl (1.4.3), colorRamp2 (0.1.0), stringi (1.8.4), shadowtext (0.1.4), data.table (1.16.2), stringr (1.5.1), dplyr (1.1.4), purrr (1.0.2), readr (2.1.5), tidyr (1.3.1), tibble (3.2.1), tidyverse (2.0.0), ggplot2 (3.5.1), and ComplexHeatmap118 (2.18.0). complexupset119(1.3.3), ggVennDiagram120(1.3.0), CORAL, CORALP, MSnbase (2.28.1), cluego(2.5.9), and cytoscape (. 3.9.1). Schematics were made using Biorender.com and Adobe Illustrator. RNAseq analysis: Nextflow and nf-core workflows handled data acquisition and processing, with alignments visualized using IGV. Mass spectrometry data processing: Mascot (v2.8) and Proteome Discoverer (v3.0), Percolator and ptmRS were part of Proteome Discoverer processing nodes.

For manuscripts utilizing custom algorithms or software that are central to the research but not yet described in published literature, software must be made available to editors and reviewers. We strongly encourage code deposition in a community repository (e.g. GitHub). See the Nature Portfolio guidelines for submitting code & software for further information.

Data

Policy information about availability of data

All manuscripts must include a data availability statement. This statement should provide the following information, where applicable:

- Accession codes, unique identifiers, or web links for publicly available datasets
- A description of any restrictions on data availability
- For clinical datasets or third party data, please ensure that the statement adheres to our policy

Raw mass spectrometry files and TMT channel mapping information can be accessed on the PRIDE database (PXD056965). Reviewers can access the data by using the following account on PRIDE: reviewer_pxd056965@ebi.ac.uk, password: k9P7cnKLj1Yb. The R image used for the RNA-seq data analysis is also available (docker://bumproo/bulk_r441).

Research involving human participants, their data, or biological material

Policy information about studies with human participants or human data. See also policy information about sex, gender (identity/presentation), and sexual orientation and race, ethnicity and racism.

Reporting on sex and gender	Sex and gender were not considered in the study design. The analyses were performed on de-identified human GBM tissue sections used solely as biological substrates for multiplexed CyCIF imaging. No stratification or comparison based on sex or gender was performed.
Reporting on race, ethnicity, or other socially relevant groupings	Race, ethnicity, or other socially relevant groupings were not considered in the study design. These variables were not available for the samples analyzed and were not relevant to the technical or analytical objectives of the study.
Population characteristics	Not applicable. This study did not involve the characterization of a human population.
Recruitment	Patients were enrolled in the clinical trial (ClinicalTrials.gov identifier: NCT03071913) and tumor samples were obtained upon informed consent. All study procedures were performed and approved by the Mayo Clinic Institutional Review Board.
Ethics oversight	The use of de-identified human GBM tissue samples complied with institutional guidelines and approvals associated with the original sample collection, as detailed in the Methods section of the manuscript. Patients were enrolled in the clinical trial (ClinicalTrials.gov identifier: NCT03071913) and tumor samples were obtained upon informed consent. All study procedures were performed and approved by the Mayo Clinic Institutional Review Board.

Note that full information on the approval of the study protocol must also be provided in the manuscript.

Field-specific reporting

Please select the one below that is the best fit for your research. If you are not sure, read the appropriate sections before making your selection.

☒ Life sciences ☐ Behavioural & social sciences ☐ Ecological, evolutionary & environmental sciences

For a reference copy of the document with all sections, see nature.com/documents/nr-reporting-summary-flat.pdf

Life sciences study design

All studies must disclose on these points even when the disclosure is negative.

Sample size	The sample size selection was guided by our prior studies indicating that n=9 was sufficient to detect drug effects in these PDX models. Given our new platform technology incorporates enrichment and physical separation to address tissue heterogeneity, we determined that n=9 would yield even more robust results for INSIGHT analysis than drug treatment studies.
Data exclusions	For all samples, phosphopeptides or proteins not detected in at least three samples per condition were excluded to ensure data robustness. EGFR-positive tumor populations from two left hemispheres (A and F) were excluded from data analysis, as they represented portions of the primary tumors that extended into the left hemisphere and inadvertently included during the mid-sagittal dissection, resulting in their high percentage of tumor cells compared to other left hemispheres during sorting (Extended Data Fig. 3c). The same phenomenon was observed in the batches of tumor-bearing brains analyzed by co-IF microscopy, and these cases were excluded from analysis as detailed in the microscopy methods section.
Replication	Experiments were designed to ensure reproducibility by analyzing multiple brains for each PDX model (GBM6 and GBM12). Key findings were further validated using complementary methods, including co-immunofluorescence and flow cytometry.
Randomization	Randomization was not relevant to this study as the experimental groups were based on inherent biological differences (primary tumor cells vs. disseminated tumor cells) within each PDX model. However, the study design controlled for potential confounding factors by comparing different cell types within the same brain to account for intra-sample variability.

Blinding	Blinding was not employed in this study, as group allocation was inherently defined by the biological characteristics of the cells (e.g., primary tumor cells vs. disseminated tumor cells) rather than by an experimental condition that could be randomized.

Reporting for specific materials, systems and methods

We require information from authors about some types of materials, experimental systems and methods used in many studies. Here, indicate whether each material, system or method listed is relevant to your study. If you are not sure if a list item applies to your research, read the appropriate section before selecting a response.

Materials & experimental systems

n/a	Involved in the study
☐	☒ Antibodies
☐	☒ Eukaryotic cell lines
☒	☐ Palaeontology and archaeology
☐	☒ Animals and other organisms
☐	☒ Clinical data
☒	☐ Dual use research of concern
☒	☐ Plants

Methods

n/a	Involved in the study
☒	☐ ChIP-seq
☐	☒ Flow cytometry
☒	☐ MRI-based neuroimaging

Antibodies

Antibodies used

Frozen whole brains embedded in OCT compound were coronally sectioned at 5 µm, and Lamin A/C (Abcam) stain was performed at intervals to identify the location of the most prominent primary tumor and disseminated tumor to count the number and percentage of tumor cells in each region identified. Two sections from two different coronal depths were stained and analyzed per PDX tumor-bearing brain for 6 brains (total n = 12 sections). Tumor burden varied across depths. When disseminated tumor cells were absent, analysis of the primary tumor and margins was still performed. Sections lacking a clear midline to delineate the left hemisphere or those with extensive primary tumor growth that pushed into the left hemisphere were excluded, consistent with the INSIGHT analysis. The tumor core, margin, and left hemisphere were drawn manually for each section for analysis. Tissues were fixed with 2% paraformaldehyde/PBS, washed with PBS, permeabilized with 0.1% Triton100/PBS, and blocked with normal serum. Tissues were stained with primary antibodies targeting the pT373-RB1 (Abcam, cat# ab52975; 1:100. We note that pT373-RB1 antibody from Invitrogen did not work), pS807/811-RB1 (CST, cat# 8516S; 1:100), pY876-GluA2 (CST, cat# 4027S; 1:100), pT693-EGFR (CST, cat# 3056S; 1:100) and were probed with goat anti-rabbit IgG-AF488 (H+L) secondary antibody (Thermo). We note that strong staining in the corpus callosum was seen for the Abcam pT373-RB1 antibody. Sections were blocked with normal serum and incubated with rabbit anti-human Lamin A/C-AF594 (Abcam, cat# ab215324; 1:400) and mouse anti-human EGFR-APC (Biolegend, cat# 352906; 1:200). Sections were stained with DAPI after being subjected to TrueView (Vectors) to distinguish nuclei, mounted using Vectashield vibrance antifade mounting medium, and images were taken using a TissueFAXS SL slide scanner (TissueGnostics) coupled to a Zeiss upright microscope with a 20x or 40x objective with z-stack enabled. All images from TissueFAXS SL were analyzed using StrataQuest (v7.1.1.143). A nuclei mask was used for the quantification of pT373-RB1 and pS807/811-RB1 and Lamin A/C while a cellular mask was used for the quantification of EGFR and pT693-EGFR. For pY876-GluA2, the FISH dot algorithm was used. For all image analyses of disseminated tumors in the left hemisphere of the G12 PDX model, each tumor cell was visually validated. For confocal microscopy, Olympus FV1200 Laser Scanning Confocal Microscope was used at 60x objective with 2x zoom, and the images were analyzed using Fiji.

Cyclic immunofluorescence (CyCIF)97 on fresh frozen core biopsy tissues was performed as previously described98. Briefly, fresh frozen patient glioblastoma samples (n=20) were sectioned at 10µm using a Cryostat (Leica) and thaw mounted on SuperfrostTM Plus microscope slides (Thermo). Mounted specimens were fixed with 4% paraformaldehyde/PBS, washed with PBS, permeabilized with 0.1% detergent solution (Thermo Scientific Cat. No. 28316) and blocked with SuperblockTM (Thermo). Specimens were stained using CyCIF, rounds of primary antibody staining and scanning followed by photobleaching to inactivate fluorophore signals before incubating with a next round of conjugated primary antibodies. Nuclear labeling was achieved with Hoechst 33342 (Cat. H3570) diluted 1:10,000. Phosphorylated GluA2 at tyrosine 876 was detected using a rabbit monoclonal antibody against GluA2 pY876 (Cat. 4027S, clone pY876; unconjugated) at a 1:100 dilution. Neuronal nuclei were labeled with a rabbit anti-NeuN antibody (Cat. ab190195, clone EPR12763) directly conjugated to Alexa Fluor 488 at 1:100. Other antibodies used were rabbit anti-OLIG2 (Cat. ab300730, clone EPR2673) conjugated to Alexa Fluor 555 at 1:100, rabbit anti-SOX2 (Cat. ab300548, clone EPR22952-70) conjugated to Alexa Fluor 555 at 1:100, and rabbit anti-SOX9 (Cat. ab196184, clone EPR14335) conjugated to Alexa Fluor 647 at 1:100. All primary antibodies were diluted in Hoechst 33342 and incubated overnight at 4oC in the dark. Specimens were photobleached in a solution of 4.5% H2O2, 24mM NaOH in PBS for 1 hour under an LED light source to inactivate fluorophores. Scanning was performed on EVOS S1000 Spatial Imaging System (Thermo), stitching and registration of cycles were done in MCMICRO99 using ASHLAR100 modules. Additional details and code can be at found at www.cycif.org.

Flow cytometry and sorting
All flow cytometric analyses were done using BD High Throughput Sampler (HTS) coupled to LSRFortessa (BD Biosciences). Anti-EGFR-PE (AY-13) antibody (Biolegend, cat# 352904; 1:200) was used to stain and identify tumor cells from normal cells for the samples subjected to mass spectrometry. For all sorting experiments, Sony MA900 (70 µm nozzle) was used to sort cells into 5ml tubes. Cells were stained with anti-O4 antibody-APC (Miltenyi cat# 130-119-897; 1:100) to isolate oligodendroglial cells. For PDX tissues, normal mouse serum (Invitrogen), Human TruStain FcX Fc Receptor Blocking Solution and TruStain FcX™ (anti-mouse CD16/32) Antibody (Biolegend), and True Stain Monocyte Blocker (Biolegend) were added according to manufacturer instructions before staining cells for minimum 30 minutes. For testing fixatives, anti-EGFR-BV421 was used (cat# 352911; 1:200) with a gating strategy shown in Supplementary Figure 11a. Mixture of normal and TdTomato+ tumor cells were stained with anti-HLA-A/B/C-APC-Cy7 [W6/32]

	antibody (Biolegend, cat# 311426; 1:2000) or anti-EGFR-BV421 antibody (Biolegend, Cat# 352911, 1:100-1:200) (gating strategy in Supplementary Figure 11b). Anti-mouse pan cytokeratin antibody (AE1/AE3) – Alexa Fluor 488 (cat# 53-9003-82; Thermo; 1:200) was used to stain the 4T1 breast tumor cells. For dissociated PDX tumor-bearing brains, cells were first gated on FSC-A vs. SSC-A to exclude debris and aggregates, followed by FSC-W vs. FSC-H and SSC-H vs. SSC-A to isolate singlets, after which cells were gated on EGFR-PE vs. SSC-A to separate EGFR-positive and EGFR-negative populations (Supplementary Figure 11c). Sorted cells were spun down to remove supernatant and pellets were stored at -80°C before sample processing for mass spectrometry.
Validation	All antibodies used except for CyCIF were cross validated using an orthogonal method(s). The data obtained from microscopy, flow cytometry, and mass spectrometry demonstrated strong alignment, validating the specificity and reliability of the antibodies used.

Eukaryotic cell lines

Policy information about cell lines and Sex and Gender in Research

Cell line source(s)	G6 and G12 cell lines were derived from GBM patients. LN229 was acquired from ATCC. HEK293T cells already existed in the lab and we cannot be sure of the original source
Authentication	HEK293T cells were not authenticated. LN229 was authenticated by ATCC
Mycoplasma contamination	All cell lines were tested negative for mycoplasma
Commonly misidentified lines (See ICLAC register)	*Name any commonly misidentified cell lines used in the study and provide a rationale for their use.*

Animals and other research organisms

Policy information about studies involving animals; ARRIVE guidelines recommended for reporting animal research, and Sex and Gender in Research

Laboratory animals	C57BL/6. Nude mice.
Wild animals	NA
Reporting on sex	The study utilized female nude mice for intracranial inoculations of GBM6 and GBM12 PDX tumors, as specified in the methods. Sex was considered in the study design to align with prior work on glioblastoma models, where we employed female mice to control for potential sex-specific effects in tumor behavior. No additional sex-based analyses were conducted, as the focus was on tumor cell signaling networks and not on sex-related differences in glioblastoma dissemination.
Field-collected samples	All samples were derived from laboratory-maintained tumor-bearing animal models.
Ethics oversight	male and female C57BL/6 mice were acquired from Taconic Biosciences, maintained, and necropsied in compliance with protocols approved by the Committee on Animal Care (CAC/IACUC) at MIT. All animal work related to PDX-tumor generation was done in compliance with the Mayo Institutional Animal Care and Use Committee.

Note that full information on the approval of the study protocol must also be provided in the manuscript.

Clinical data

Policy information about clinical studies

All manuscripts should comply with the ICMJE guidelines for publication of clinical research and a completed CONSORT checklist must be included with all submissions.

Clinical trial registration	NCT03071913
Study protocol	ClinicalTrials.gov
Data collection	For MRI-guided multiregional biopsy of five GBM patients (2 tissue biopsies from 3-4 tumor locations; n=20 specimens), all patients underwent pre-operative MRI to identify biopsy targets, and intra-operative image-guided Stealth neuro-navigation was used to define the image-based location of each sample. Each core biopsy was flash frozen. Patients were enrolled in the clinical trial (ClinicalTrials.gov identifier: NCT03071913) and tumor samples were obtained upon informed consent. All study procedures were performed and approved by the Mayo Clinic Institutional Review Board.
Outcomes	The outcome was irrelevant for our manuscript

Plants

Seed stocks: NA

Novel plant genotypes: NA

Authentication: NA

Flow Cytometry

Plots

Confirm that:

☒ The axis labels state the marker and fluorochrome used (e.g. CD4-FITC).

☒ The axis scales are clearly visible. Include numbers along axes only for bottom left plot of group (a 'group' is an analysis of identical markers).

☒ All plots are contour plots with outliers or pseudocolor plots.

☒ A numerical value for number of cells or percentage (with statistics) is provided.

Methodology

Sample preparation: As part of the INSIGHT platform, frozen brains were cryosectioned, fixed with commercial fixatives, physically and enzymatically dissociated, filtered with 40 um and 30 um filters, stained with antibodies of choice, and sorted.

Instrument: Sony MA900

Software: Sony MA900 software

Cell population abundance: Disseminated tumor cells, primary tumor cells, and normal brain cell populations were isolated and subjected to phosphoproteomics and protein expression analysis by mass spectrometry. Tumor and normal cell-specific marker expressions were assessed along with their biological processes to determine the proper enrichment of the targeted cell subpopulations.

Gating strategy: for sorting, cells were first gated on FSC-A vs. SSC-A to exclude debris, aggregates and isolate cells, followed by FSC-W vs. FSC-H and SSC-H vs. SSC-A to isolate singlets. Finally, cells were gated on EGFR-PE fluorescence intensity vs. SSC-A to separate EGFR-positive and EGFR-negative populations.

☒ Tick this box to confirm that a figure exemplifying the gating strategy is provided in the Supplementary Information.
