# Supplementary material for: Uncovering the signaling networks of disseminated glioblastoma cells in vivo with INSIGHT
Source: Nat Commun. 2026 Aug 8;17:9536. doi: 10.1038/s41467-026-76587-0 (PMC13547291; doi:10.1038/s41467-026-76587-0)
Supplement: Supplementary file 2 [file 41467_2026_76587_MOESM11_ESM.pdf]

nature portfolio

Peer Review File

Uncovering the signaling networks of disseminated glioblastoma cells in vivo with INSIGHT

Corresponding Author: Professor Forest White

This file contains all reviewer reports in order by version, followed by all author rebuttals in order by version.

Version 0:

Reviewer comments:

Reviewer #1

(Remarks to the Author)
In the manuscript titled "Uncovering the signaling networks of disseminated glioblastoma cells in vivo with INSIGHT," the authors present a novel phosphoproteomic platform, INSIGHT, to investigate how glioblastoma (GBM) cells disseminate throughout the brain. They focus on rare tumor cell populations that are thought to drive recurrence and disease progression. Their analysis shows that these disseminated cells activate synaptic and neuronal migration pathways and shift toward mesenchymal and neural progenitor-like states. Interestingly, despite EGFR amplification in the primary tumor, EGFR signaling appears suppressed in the disseminated cells. The authors identify GluA2 (pY876) and Hornerin (HRNR) as potential drivers of GBM invasion.

This is a technically impressive and ambitious study, but there are several points that could be strengthened:

The clinical relevance of GluA2-pY876 is not yet clear. It would be important to test this finding in more representative GBM models and to evaluate its presence or activity in human tumor samples.

The study only examines one relatively late time point. Including earlier time points could offer valuable insights into how signaling changes develop during the dissemination process.

The work is based on just two PDX models (GBM6 and GBM12), both of which are EGFR-driven. It's unclear whether the findings apply to other GBM subtypes that don't rely on EGFR signaling.

While some functional validation is provided, additional in vivo experiments would strengthen the conclusions. For example, testing phospho-dead mutants of key candidates (such as GluA2) in orthotopic models would add important evidence.

The role of glioblastoma stem-like cells (GSCs) in dissemination isn't addressed. Since GSCs are known to contribute to invasion and recurrence, it would be useful to know whether the disseminated populations studied here show enrichment for stem-like markers or features.

The question of whether disseminated GBM cells are more resistant to temozolomide is highly relevant but not explored. These cells are less proliferative and more mesenchymal, which are both features associated with treatment resistance. Testing TMZ sensitivity or at least discussing the potential implications would add to the translational impact of the work.

Reviewer #2

(Remarks to the Author)
This paper presents an important advance in our ability to measure the functional state of disseminated tumor cells using proteomics and phosphoproteomics analysis. Although the great majority of traditional studies on the functional "state" of in vivo tumor cells have used transcriptomics profiling, transcriptomics only provides a crude approximation of cell state. Proteomics and phosphoproteomics analyses are widely acknowledged to be superior in their ability to assess cell state because they can directly measure the abundance and modification state of key regulatory proteins. Unfortunately, most techniques for isolating tumor cells have a profoundly negative impact on proteomics and phosphoproteomics profiling. Thus, assessing the functional state of disseminated tumor cells has mostly been restricted to cyclical immunofluorescence

or similar antibody-based techniques, which are both complex and of limited use for discovery-oriented research.

The current paper provides a proof-of-concept for a technique that could profoundly improve our ability to understand how tumors change their functional state in both different parts of a tumor and during metastasis. They demonstrate that it is possible to fix cells in frozen tissue sections, enzymatically dissociate them and then isolate significant quantities of cells for analysis by flow cytometry. Frankly, I am very surprised that the technique actually works, but they provide compelling evidence that it does. Hats off to the research team for coming up with this innovative approach! They then demonstrate that one can derive very important information from the proteomic/phosphoproteomic state of the cells, such as a shift from a cell proliferation to a more mesenchymal, neural progenitor-like state. There are numerous, other very interesting observations that they make regarding disseminated tumor cells, all which show the power of proteomics/phosphoproteomics profiling to yield important new insights into the physiological state of cancer cells.

The paper also well written and I found very little to find fault with, other than a bit of overselling their “platform” and results (understandable in todays ultra-competitive publishing environment). However, they should be more transparent in pointing out the current limitations of the technique and their results. First, they are only analyzing two xenograft models, a number that is far too small to reach any general conclusions about disseminated GBM cells, although their results are consistent with several prior studies. Other caveats that should be mentioned:

1. They have only tested this on brain tissue. The dissociation of other metastatic targets (e.g. lung, liver) might require an entirely different enzymatic digestion approach.

2. They are using abundant, GBM tumor-specific cell surface markers to isolate the tumor cells. It would be nice to show the ability to use cytoplasmic markers of some combination of tags for other types of tumors.

3. They are isolating human cells from mouse tissue and then excluding the mouse-specific peptides during the MS data analysis. This improves the apparent enrichment of the tumor cell achieved by flow cytometry. When this technique is applied to isolating human tumor cells from human tissues, they will not be able to use this “trick”. The authors should indicate the impact of filtering out mouse peptides on the overall enrichment they achieve for the tumor cells.

Reviewer #3

(Remarks to the Author)
Summary
In this study, Ahn et al. have developed an innovative approach to perform phosphoproteomics on subsets of cell types from tissue samples. The method involves fixing cells prior to dissociation, followed by cell sorting and sample preparation for mass spectrometry-based proteomics and phosphoproteomics. This method addresses key challenges in comparing phosphoproteomes across cell types, especially when cells are disseminated. The authors present their optimisation of their method, such as testing different fixatives. However, the approach does cause some changes to the proteome and phosphoproteome. Including analyses of these altered sites and proteins would help future users to understand the trade-offs in using this method, and guide future improvements to the approach. This could be achieved through additional bioinformatics analyses.
As proof-of-principle, the authors applied their method to compare oligodendrocyte lineage cells and non-oligodendrocyte lineage cells from mouse brain tissue. The main application was comparing disseminated and primary glioblastoma cells in two different xenograft models of glioblastoma in mice (either EGFR amplification or activating mutation). This application highlights the strength of the method, which enables purification of disseminated cell types. The authors identified altered phosphosites in disseminated compared to primary tumor cells, including GluA2 Y876 phosphorylation, which they experimentally validated as impacting invasion phenotypes in LN229 cells. The regulatory kinase is listed as Src, but this is based off predictions, which may be complemented through complementary analyses and clear reporting, as the kinase may also be Fyn.
With added bioinformatic analyses and transparent reporting of potential limitations of the method, this work could benefit many readers interested in cell type-specific phosphoproteomics.

Major comments
1. Lines 109-110 state that “fixation methods preserved both the phosphoproteome and the whole proteome (Fig. 1b).” This claim is a little strong. Fixation altered 12% of the measured proteome (~503 proteins), 7% of the measured pS/T phosphoproteome (~559 phosphopeptides) and 11% of the pY phosphoproteome (~122 phosphopeptides) (Fig. 1b). This difference isn’t a major concern, as trade-offs are common in sample preparation methods. Because this method enables analyses that aren’t possible with other approaches, the trade-off could likely be worthwhile for certain applications. A key addition that would strengthen the manuscript is:
A. A transparent presentation of this difference in the results section.
B. Analyses that would give researchers considering this approach the information they need about the trade-off involved and how they might address the method's current limitations through future adaptations. For example, I’d like to get a sense of the kinds of proteins that are lost (since this shift towards only losing proteins is an important technical artefact), in a more in-depth look than the current analysis comparing peptide length. For example, what gene ontology cellular components are they annotated as? Are they enriched in extracellular proteins? Similarly for the phosphosites, are they dominated by known and predicted substrates of certain kinases or phosphatases?
C. It would also strengthen the manuscript to flag proteins and phosphosites altered by fixation that were regulated in the xenograft experiments, ideally in a supplementary table. It would also be helpful to specify if this applies to any hits

mentioned in the results. Given that all samples in these xenograft experiments were fixed and sorted, it could have a consistent effect and not be a problem. However, since it hasn't been established how fixation-induced alterations may vary across cell types, it would be worth noting.

2. Lines 312-318 report that G6 increases GRIA2/GluA2-pY876, whereas G12 increases GRIA1/GluA1. However, in Fig. 4e, it appears that GRIA2 total protein levels are not regulated in either model, and GRIA2 Y876 is only quantified in G6, not G12. This highlights a broader issue: differences between G6 and G12 may arise from a lack of quantification rather than true regulation. Since G6 and G12 samples are measured in separate TMT batches (Fig. 1a), cross-batch comparisons could be impacted by missing values due to stochastic quantification. Clarifying whether differences in the significant hits in each model reflect true regulation or missing data would help readers interpret these results more confidently. The same clarification would be valuable for ion channels. How many of the ion channels listed as regulated in G12 were quantified in G6 but not regulated, rather than not quantified at all?

3. Several biological hypotheses are based on enrichment of predicted kinases, identified from kinase library motif specificies among the regulated phosphosites. These analyses are performed well. While the kinase library is a valuable resource and one effective approach, clarity would be improved by highlighting that these are predicted kinases for the sites, rather than bona fide kinases that have been experimentally validated (e.g., by inhibitor studies and in vitro kinase assays of the entire protein). Because of these, I recommend the following changes to the text, and bioinformatic analyses:
A. At multiple places in the text (lines 233-239, title in figure of Fig. 3e, line 347, 1264, 1302), clarity of the text could be improved by including the word predicted when referring to kinase-substrate relationships from the kinase library.
B. The predicted kinase enrichment analyses would be strengthened with a separate enrichment analysis of reported substrates (e.g. from the PhosphoSitePlus database), and global analyses of altered regulatory (activating or inhibiting) phosphosites on kinases. These provide crucial orthogonal information to give a more comprehensive view of the likely active kinases. Phosphosites can fit the motifs of many kinases (Johnson et al., 2023, Fig. 3d, https://doi.org/10.1038/s41586-022-05575-3), so these additional lines of evidence can help clarify which of multiple potential active kinases may be regulatory in this case.
C. Point 3B is particularly relevant to the finding of Y876-GluA2 phosphorylation, which is described as potentially being regulated by Src. The Log2(Score) in kinase library for phosphorylated Y876-GluA2 is 3.2 for Src and 3.0 for Fyn, suggesting that either kinase could be regulatory. It was helpful to supplement the results with the mRNA and protein levels for the potential regulatory kinases in this Fig. 5c. However, there are detectable levels of both Src and Fyn in the cells, and the relative level of a kinase does not often track with activity in the way that regulation of the activating or inhibitory sites do. Expanding this analysis to compare the abundances of any regulatory phosphosites on these kinases would help disentangle this further. In addition to this analysis, Fig. 5i should be edited so that the line between SRC and GluA2-pY876 is dashed to clarify that this kinase-substrate relationship is predicted, and hasn't been experimentally validated. Given the current evidence, it would be worth including Src/Fyn into the image, as the evidence does not clearly indicate only one of these is the regulatory kinase.

4. In line 340, it mentions that pY187-ERK2 increased in G6 and was undetected in G12. Why is MAPK1 Y187 presented in the G12 heatmap in Extended data Fig 9a if it wasn't detected in G12?

5. Lines 450-452: "We also highlight that signaling circuitries of neuronal and non-neuronal programs interconnect during GBM dissemination by uncovering numerous phosphoproteins, proteins, and kinases and their relationships within those circuitries." This line should be toned down to more accurately reflect that the potential regulatory kinases were predicted, and would require future experimental validation.

Minor comments
1. It would be helpful to mention in the title and abstract that the work is done on xenografts in mice
2. Line 56-57: to supplement the comment that "phosphoproteomics requires rapid sample processing, which restricts the analysis to bulk tissues", it would be helpful to mention the recent development of deep visual phosphoproteomics, in which phosphoproteomes of laser microdissected cell populations from frozen mouse brain tissue sections were measured (Oliinyk et al., 2025, https://doi.org/10.1101/2025.05.29.656770).
3. Figure 1J: The Log2FC color scale isn't centred at 0, so 0 Log2FCs appear as blue in the graph. Please regenerate the plot with a centred color gradient, so that 0 is the middle grey color.
4. Line 212-214: When the authors state "we performed differential expression analysis of the phosphoproteome and proteome between primary tumor cells and normal cells, a task that has been previously impossible with tissue phosphoproteomics". This statement may be more accurate if it referred to measure diffuse, invasive cells, rather than primary tumor cells. The CPTAC consortium have compared primary tumor cells to normal cells in multiple tissue types including comparing high-grade gliomas to normal brain tissue (e.g. Liu et al., 2024, https://doi.org/10.1016/j.ccell.2024.06.004)
5. Line 258, 285, 311, 399, 1182, 1298, 1300, 1445, 1476, 1499: The manuscript frequently refers to the "expression" of phosphopeptides. Given that "expression" generally describes the synthesis of mRNA or a protein, this usage may be misleading. Phosphopeptide levels are influenced by both phosphorylation and dephosphorylation, covering more processes than protein synthesis. I recommend revising the terminology to "abundance" or "levels" to improve accuracy.
6. It would be helpful if the methods section specified the threshold of the site percentile or Log2(Score) used from kinase library to create the sets of predicted substrates that were analysed in enrichment analysis.
7. Figure 4d: It could be helpful to specify that this enrichment was done on the proteome abundance ranks rather than mRNA levels, as it isn't explicitly mentioned in the results text or the figure legend, but the results currently mention a "gene signature" and "top-ranked genes", so it could be a place for confusion.
8. The article often describes results as "significantly increased" (line 334, 363) or "showed a downward trend" (line 251)

without sharing the fold-changes or p-values in the text. Including these would help readers interpret the findings.

Version 1:

Reviewer comments:

Reviewer #1

(Remarks to the Author)
The authors have addressed the concerns raised and the manuscript is improved.

Reviewer #2

(Remarks to the Author)
The authors successfully addressed the points that I raised in the original review as well as those of the other reviewers (IMHO). I really liked the original version and this one is even better. The authors are to be commended for such a prompt and responsive revision.

Reviewer #3

(Remarks to the Author)
The authors have done an excellent job addressing the comments. They have put in a substantial effort to strengthen their manuscript, increase the utility of their resource, and aid future researchers in successfully adopting this method. This work provides an exciting and highly valuable contribution towards understanding cell-specific signaling in complex tissues.

The authors have fully addressed my comments, and I have no further requests.

Open Access This Peer Review File is licensed under a Creative Commons Attribution 4.0 International License, which permits use, sharing, adaptation, distribution and reproduction in any medium or format, as long as you give appropriate credit to the original author(s) and the source, provide a link to the Creative Commons license, and indicate if changes were made.
In cases where reviewers are anonymous, credit should be given to 'Anonymous Referee' and the source.
The images or other third party material in this Peer Review File are included in the article's Creative Commons license, unless indicated otherwise in a credit line to the material. If material is not included in the article's Creative Commons license and your intended use is not permitted by statutory regulation or exceeds the permitted use, you will need to obtain permission directly from the copyright holder.
To view a copy of this license, visit https://creativecommons.org/licenses/by/4.0/

Response to reviewer comments

Ahn et al.
Nature Communications manuscript NCOMMS-25-39447-T

We sincerely appreciate the reviewers' comments on our manuscript entitled "Uncovering the signaling networks of disseminated glioblastoma cells in vivo with INSIGHT". As noted in the specific responses below, to address the concerns raised by the reviewers, we have added a substantial amount of new data to the manuscript that we believe further strengthens the novelty, impact, and broad applications of this methodology.

Reviewer #1 (Remarks to the Author): clinician, GBM biology and PDXs

In the manuscript titled "Uncovering the signaling networks of disseminated glioblastoma cells in vivo with INSIGHT," the authors present a novel phosphoproteomic platform, INSIGHT, to investigate how glioblastoma (GBM) cells disseminate throughout the brain. They focus on rare tumor cell populations that are thought to drive recurrence and disease progression. Their analysis shows that these disseminated cells activate synaptic and neuronal migration pathways and shift toward mesenchymal and neural progenitor-like states. Interestingly, despite EGFR amplification in the primary tumor, EGFR signaling appears suppressed in the disseminated cells. The authors identify GluA2 (pY876) and Hornerin (HRNR) as potential drivers of GBM invasion.

This is a technically impressive and ambitious study, but there are several points that could be strengthened:

1. The clinical relevance of GluA2-pY876 is not yet clear. It would be important to test this finding in more representative GBM models and to evaluate its presence or activity in human tumor samples.

   During revision, we have analyzed and incorporated multiregional GBM patient tissues in **Figure 5j-k**, demonstrating that GluA2-pY876 is significantly enriched at the neuron-GBM interface relative to the tumor core and normal tissue. Although the tumor markers available in patient samples limit definitive assignment of GluA2-pY876 to either tumor cells or neurons, these data provide the first evidence in patients that GluA2-pY876 is a prominent feature of the neuron-GBM junction. Supported by our PDX and in vitro findings, these results suggest that GluA2-pY876 may be involved in neuron-GBM interactions at the margin and at distant site and associated GBM invasiveness.

   The following text has been added to the Result section: "To assess the clinical relevance of GluA2-pY876 in human GBM, we examined surgical specimens from five patients undergoing tumor resection. For each patient, two biopsies were collected from three to four spatially distinct regions, yielding a total of 20 specimens. Cyclic immunofluorescence (CyCIF) analysis of the tissue samples revealed that pY876-GluA2+ cells were enriched in tumor regions characterized by close apposition of glioma cells and neurons, defined by the co-occurrence of >20% tumor cells ($SOX2^+$, $OLIG2^+$, and/or $SOX9^+$) and >20% $NeuN^+$ neurons. In contrast, the frequency of pY876-GluA2+ cells was lower in glioma-enriched regions (>20% $SOX2^+$, $OLIG2^+$, and or $SOX9^+$ with <5% $NeuN^+$) and in neuron-enriched regions (>20% $NeuN^+$ with <5% $SOX2^+$, $OLIG2^+$, and $SOX9^+$). These findings indicate that Y876-GluA2 phosphorylation is preferentially associated with

microenvironments where glioma-neuron interactions occur, supporting a role for this signaling event in tumor-neural crosstalk (Fig. 5j)."

2. The study only examines one relatively late time point. Including earlier time points could offer valuable insights into how signaling changes develop during the dissemination process.

   We agree with the reviewer that examining earlier time points during the dissemination process could provide invaluable insight into the temporal evolution of signaling programs. However, our primary objective in this work was to establish and apply, for the first time, a cell type-specific signaling network analysis framework to capture the molecular state of disseminated GBM cells *in vivo*. The systematic analysis across multiple time points, including in-depth biological analysis of these data and follow-up validation studies, would extend beyond the design and intent of this work and would constitute a separate set of manuscripts in its own right. Certainly, the framework presented here is explicitly suited for such future studies.
   We have expanded the Discussion to note that applying this approach across earlier and intermediate time points could enable reconstruction of the signaling trajectories that accompany dissemination, providing a powerful avenue for future investigation. In this context, the present study should be viewed as a foundational analysis that defines the disseminated state, upon which temporal and treatment-based extensions can be built. We believe this clarification appropriately acknowledges the importance of temporal dynamics while maintaining a focused and coherent scope for the current manuscript. The discussion now includes a text as follows: "While temporal dynamics are not explicitly addressed here, INSIGHT provides a scalable framework for future studies aimed at reconstructing signaling trajectories across dissemination. Accordingly, this work defines a molecular baseline for the disseminated state that can be extended to incorporate temporal and therapeutic perturbations."

3. The work is based on just two PDX models (GBM6 and GBM12), both of which are EGFR-driven. It's unclear whether the findings apply to other GBM subtypes that don't rely on EGFR signaling.

   Multiple lines of evidence suggest that our findings are likely not restricted to EGFR-driven glioblastoma. First, prior studies have reported the presence of GluA2 or increased GluA2-associated Ca2+ influx to promote tumor progression in both glioma and glioblastoma models that are not EGFR-driven (PMID: 31534222, 11717408, 31534219), yet the downstream signaling mechanisms and their functional importance remained undefined. Using INSIGHT, we delineate the in vivo signaling circuitry linked to GluA2 for the first time and demonstrate that the phosphorylation of Y876-GluA2 preferentially regulates invasion rather than migration, establishing functional relevance at the signaling level. Importantly, although both GBM6 and GBM12 are EGFR-driven, they exhibited divergent signaling behaviors at times (Figure 4e, 5a-b) in addition to their level of invasiveness (Extended Data Figure 3e,f) and only the disseminated GBM6 cells displayed differential GluA2 signaling activity. This dissociation between EGFR status and GluA2 signaling supports the notion that the engagement of this circuitry may reflect a broader invasive strategy that is not contingent on EGFR and may extend to other glioma subtypes. Second, HRNR has been linked to invasive phenotypes in several non-EGFR-dependent cancers, albeit with limited mechanistic characterization (PMID: 27382389, 30103712, 22727333, 28916756). Our identification of HRNR-associated signaling in GBM provides the first in vivo evidence for its involvement in GBM and supports a general role for HRNR related programs across tumor contexts, potentially including non-EGFR-driven GBMs. Third, the go-or-grow paradigm has been described across diverse non-EGFR tumor models, both within and beyond glioblastoma, primarily at transcriptional and phenotypic levels, and its underlying signaling architecture has not been resolved in vivo at the proteome or phosphoproteome scale.

Our data recapitulate hallmark features of disseminating and invasive states defined in those prior studies while newly revealing their coordinated signaling organization in vivo. Thus, we believe our data may represent dissemination--associated signaling modes that are not intrinsically tied to EGFR-driven GBM biology.

Future studies can directly test the generalizability of our findings across GBM subtypes by applying INSIGHT to non-EGFR tumors. For PDX models lacking EGFR expression, fluorescent genetic labeling of tumor cells combined with INSIGHT-based isolation, as illustrated in Extended Data Fig. 2d, provides a straightforward alternative to using an EGFR-specific antibody. During manuscript revision, we further demonstrated INSIGHT's flexibility by enabling the targeted isolation of tumor cells using cytoplasmic markers (**Extended Data Fig. 2f**). Furthermore, we implemented an alternative surface marker CD15, which has been reported to be expressed in subsets of GBM, to profile primary GBM patient samples that do not express EGFR (**Extended Data Fig. 3a-d**). Importantly, cells that are CD15 positive, but negative for myeloid marker CD84, closely resembled some of the human GBM cell lines as profiled by Cancer Cell Line Encyclopedia (927 cell line database). Together, these complementary strategies demonstrate that INSIGHT is broadly applicable beyond EGFR-driven tumors and PDX systems, enabling the study of diverse GBM tissues irrespective of EGFR status. Regardless, given approximately 40% of GBMs exhibit EGFR amplification and/or overexpression, the EGFR-based implementation presented here remains broadly relevant and represents an important use case for INSIGHT.

4. While some functional validation is provided, additional in vivo experiments would strengthen the conclusions. For example, testing phospho-dead mutants of key candidates (such as GluA2) in orthotopic models would add important evidence.

   As part of revision, we performed orthotopic intracranial injections of GBM6 cells expressing GluA2 wild-type, the R607Q mutant (increased Ca2+ influx), or the Y876F/R607Q double mutant (**Figure 5i**). While the expression of the R607Q mutant resulted in increased tumor growth at endpoint compared to wild-type, the Y876F/R607Q mutant did not reach statistical significance relative to either wild-type or R607Q alone, largely due to inter-animal heterogeneity. These data indicate that, in the context of the R607Q background, we cannot conclusively establish a requirement for Y876 phosphorylation in driving bulk tumor *growth* in vivo. Nevertheless, the consistent enrichment of GluA2-Y876 phosphorylation in disseminated tumor cells, the role of it increasing invasion and its strong association with neuron-tumor interface in patient-derived data (**Figure 5j-k**) support a model in which phosphorylation of GluA2-Y876 contributes to tumor-neuron interactions and invasion rather than primary tumor growth. We have now added the following text in the result section: "Orthotopic implantation of GBM6 cells expressing GluA2 wild-type, the R607Q mutant, or the Y876F/R607Q mutant demonstrated that enhanced Ca2+ permeability via R607Q increased tumor growth, whereas the contribution of Y876 phosphorylation to tumor growth could not be conclusively established."

5. The role of glioblastoma stem-like cells (GSCs) in dissemination isn't addressed. Since GSCs are known to contribute to invasion and recurrence, it would be useful to know whether the disseminated populations studied here show enrichment for stem-like markers or features.

   To address the contribution of glioblastoma stem-like cells to tumor dissemination, we assessed the degree of enrichment of glioblastoma or glioma stem-like signatures in disseminated tumor cells relative to primary tumor cells (newly added **Extended Data Fig. 7a**). To do so, we compiled five independently published glioma stemness gene signatures from the literature (Yan et al, Suvà et al, Patel et al, Tirosh et al, and Venteicher et al.) and evaluated their enrichment in our dataset.

While this analysis provides useful context, it is important to note that these signatures were defined at the transcriptomic level, whereas our data were defined at the proteomic and phosphoproteomic level. Given the poor concordance between the abundance of mRNA, protein, and post-translational modification, direct application of transcript-derived stemness programs to proteomic data has inherent limitations. Furthermore, the stemness signatures in the literature exhibited substantial methodological and biological heterogeneity. For example, Yan et al. defined a CD133-positive stem-like signature by comparing CD133-positive GBM cells to CD133-negative GBM and non-GBM cells from patient tumor cores using microarray-based differential expression. In contrast, Suvà et al. derived stemness programs from in vitro GBM models cultured under serum-free versus serum conditions, capturing a serum-regulated differentiation axis in which epigenetically stable tumor-propagating cells uniquely retain in vivo tumor-initiating capacity. Patel et al. used a similar serum-regulated culture system on dissociated patient samples to define stemness signatures from spherogenic GBM stem-like cultures capable of initiating tumors in mice. We additionally included signatures from Tirosh et al., in which stemness genes were defined by principal component analysis of IDH-mutant oligodendroglioma cells to identify poorly differentiated transcriptional states, and from Venteicher et al., who used single-cell analysis of IDH-mutant astrocytoma to assess the presence of stemness programs across brain tumor contexts.

When applied to our data, these signatures showed divergent behavior. The CD133 based signature from Yan et al. was strongly enriched in primary tumors, suggesting that disseminated tumor cells do not resemble CD133 positive stem like populations found in the tumor core. In contrast, the Patel et al. signature showed only a weak enrichment trend, highlighting inconsistencies across stemness programs. Overall, our data do not support a strong or consistent coupling between dissemination of PDX tumor cells and published glioblastoma or glioma stemness signatures.

In contrast, as shown in Figure 4a and Extended Data Figure 8a-b, disseminated GBM cells showed the consistent enrichment of mesenchymal GBM signature, a cell state that has been closely linked to cellular plasticity and therapeutic resistance in GBM. Together, our data indicate that while disseminated GBM cells do not strongly align with established transcriptional GSC signatures from tumor core or in vitro cultures, they exhibit proteomic and signaling features consistent with invasive, stem-adjacent states such as mesenchymal cell state implicated in recurrence and poor prognosis. We have added our observation on stemness signature in the Result section: “Disseminated tumor cells did not show consistent enrichment for published stemness gene signatures derived from tumor cores or in vitro models (Extended Data Fig. 7a).”

6. The question of whether disseminated GBM cells are more resistant to temozolomide is highly relevant but not explored. These cells are less proliferative and more mesenchymal, which are both features associated with treatment resistance. Testing TMZ sensitivity or at least discussing the potential implications would add to the translational impact of the work.

   We fully agree with the reviewer that the question of TMZ resistance in disseminated GBM cells is highly relevant and of clear translational interest. Indeed, this was an aim of a recently submitted R01 proposal. The observation that disseminated cells are less proliferative and exhibit mesenchymal features is indeed consistent with phenotypes previously associated with therapy resistance. However, as noted in our response to comment #1 above, this analysis of therapeutic response of disseminated tumor cells relative to the tumor core is well beyond the scope of the current manuscript.

In light of the importance of this question and the capacity of INSIGHT to address it, we have expanded the Discussion to explicitly highlight how the signaling programs enriched in disseminated GBM cells—particularly reduced proliferative signaling and enhanced mesenchymal-associated pathways—may predispose these cells to TMZ resistance. We emphasize that this raises a testable hypothesis for future studies, where the framework presented here could be directly applied to investigate treatment-induced state transitions and resistance mechanisms. The modified text is: “The signaling programs enriched in disseminated GBM cells are consistent with phenotypes previously associated with relative resistance to cytotoxic therapies such as TMZ, suggesting a testable framework for future studies interrogating treatment-induced state transitions using INSIGHT.” We hope that this discussion appropriately situates the translational relevance of TMZ resistance while maintaining a clear and focused scope for the current manuscript.

Reviewer #2 (Remarks to the Author): computational modeling of signaling network, multi-modal data integration, proteomics and some phosphoproteomics

This paper presents an important advance in our ability to measure the functional state of disseminated tumor cells using proteomics and phosphoproteomics analysis. Although the great majority of traditional studies on the functional “state” of in vivo tumor cells have used transcriptomics profiling, transcriptomics only provides a crude approximation of cell state. Proteomics and phosphoproteomics analyses are widely acknowledged to be superior in their ability to assess cell state because they can directly measure the abundance and modification state of key regulatory proteins. Unfortunately, most techniques for isolating tumor cells have a profoundly negative impact on proteomics and phosphoproteomics profiling. Thus, assessing the functional state of disseminated tumor cells has mostly been restricted to cyclical immunofluorescence or similar antibody-based techniques, which are both complex and of limited use for discovery-oriented research.

The current paper provides a proof-of-concept for a technique that could profoundly improve our ability to understand how tumors change their functional state in both different parts of a tumor and during metastasis. They demonstrate that it is possible to fix cells in frozen tissue sections, enzymatically dissociate them and then isolate significant quantities of cells for analysis by flow cytometry. Frankly, I am very surprised that the technique actually works, but they provide compelling evidence that it does. Hats off to the research team for coming up with this innovative approach! They then demonstrate that one can derive very important information from the proteomic/phosphoproteomic state of the cells, such as a shift from a cell proliferation to a more mesenchymal, neural progenitor-like state. There are numerous, other very interesting observations that they make regarding disseminated tumor cells, all which show the power of proteomics/phosphoproteomics profiling to yield important new insights into the physiological state of cancer cells.

The paper also well written and I found very little to find fault with, other than a bit of overselling their “platform” and results (understandable in today’s ultra-competitive publishing environment). However, they should be more transparent in pointing out the current limitations of the technique and their results. First, they are only analyzing two xenograft models, a number that is far too small to reach any general conclusions about disseminated GBM cells, although their results are consistent with several prior studies. Other caveats that should be mentioned:

We thank the reviewer for the helpful feedback. We have expanded our Result and Discussion section to clarify key limitations of our work, and they read as follows:

"Further investigation across a larger and more diverse cohort of EGFR driven and non-EGFR driven GBM xenograft models and patient specimens will be essential to define the broader mechanisms underlying GBM dissemination. INSIGHT provides a robust framework for systematically interrogating these processes in vivo."

"Several limitations of our study and of the INSIGHT platform remain, and addressing these through continued methodological refinement and further investigation are needed. First, fixation can negatively impact the epitope recognition of some antibodies. Antibodies targeting cytoplasmic markers and those used for immunohistochemistry will broaden the range of detectable markers. Second, we employed data-dependent acquisition, which, while effective for discovery-based proteomics, is inherently limited by stochastic precursor selection, impacting the reproducibility across experiments. Targeted acquisition strategies will increase sensitivity, reproducibility, and precision91,92. Third, dyes that rely on membrane integrity for live/dead cell discrimination become ineffective with fixation. While INSIGHT performed effectively in both normal and tumor-bearing tissues, strategies to discern live/dead cells without relying on membrane impermeability would minimize noise. Fourth, given that INSIGHT utilizes cryosections, sampling parallel tissue sections with spatial-omics technologies will increase the depth of biological insight. Concurrently, tissue sectioning likely results in underrepresentation of cell populations with extensive projections, owing to their increased susceptibility to structural disruption during processing. Fifth, as with all flow cytometric and microfluidic platforms, the identification and isolation of rare cell populations from large cellular backgrounds remains technically challenging, particularly when balancing processing speed, throughput, and feasibility with the goal of achieving near complete purity."

1. They have only tested this on brain tissue. The dissociation of other metastatic targets (e.g. lung, liver) might require an entirely different enzymatic digestion approach.

   We have further performed tissue dissociation on mouse liver, lung, spleen, in addition to brain, (shown in **Extended Data Figure 2c**). We agree that organ-specific enzymatic digestion strategies may be optimal for different tissues and will require further investigation. However, for the samples presented in Extended Data Figure 2c, collagenase III and the described protocol were sufficient to efficiently extract cells. The text in the manuscript was updated to:
   "We also quantified cell yields from the mouse liver, lung, spleen, and brain, demonstrating the potential applicability of INSIGHT across different tissues (Extended Data Fig. 2c)"
   "Cytoplasmic pan-cytokeratin could also be detected in 4T1 breast cancer tissue and lung from 4T1 breast cancer-bearing mice (Extended Data Fig. 2f). Collectively, these results highlight that fluorescently tagged proteins or cytoplasmic proteins may be targeted by INSIGHT to characterize the phosphoproteome and protein expression of cells, broadening the applicability of INSIGHT in genetically engineered model organisms."

2. They are using abundant, GBM tumor-specific cell surface markers to isolate the tumor cells. It would be nice to show the ability to use cytoplasmic markers of some combination of tags for other types of tumors.

   During manuscript revision, we further optimized the workflow to enable the detection of cytoplasmic proteins following permeabilization, as shown by pan cytokeratin detection in 4T1 breast tumor cells (**Extended Data Fig. 2f**). We wish to note several important limitations with this

approach: antibody access into cytoplasm requires permeabilization, which increases cellular leakiness and reduces effective cell density, necessitating higher centrifugal forces for pelleting. Although epitope-specific antibody detection is feasible, permeabilization inevitably would result in the loss of proteins and phosphoproteins, thereby leading to the loss of information. We want to note that the method is compatible with endogenous fluorescently tagged proteins, as demonstrated in Extended Data Fig. 2e, and where genetic modification is possible, this would circumvent the need for the use of antibodies against cytoplasmic proteins.

3. They are isolating human cells from mouse tissue and then excluding the mouse-specific peptides during the MS data analysis. This improves the apparent enrichment of the tumor cell achieved by flow cytometry. When this technique is applied to isolating human tumor cells from human tissues, they will not be able to use this “trick”. The authors should indicate the impact of filtering out mouse peptides on the overall enrichment they achieve for the tumor cells.

   We thank the reviewer for raising this important point. We would like to clarify that INSIGHT enables optimal enrichment and quantitative profiling of cell-type-specific proteomes and phosphoproteomes without reliance on species-dependent post-MS filtering. This is demonstrated both in mouse brain tissue, where oligodendrocytes and OPCs were selectively enriched (Fig. 1c–j), and in human glioblastoma patient samples, where tumor and myeloid cells were isolated without any species-based filtering (newly added **Extended Data Fig. 3a–d**).

   To illustrate both the rationale for and the effects of the mouse-peptide exclusion procedure, we have added **Reviewer Figure 1**, which shows the counts (1a) and reporter intensities (1b) of human-specific (h), mouse-specific (m), and shared human-mouse (hm) protein identifications with or without the exclusion of mouse-specific peptides. The reported counts represent IDs with non-zero TMT reporter intensity across all TMT channels in the plex, which included tumor cells, normal cells, and bulk dissociated tissue. Their corresponding TMT reporter intensities (1b) show that murine normal cell samples exhibit residual intensities for human proteins, whereas human tumor cells exhibit residual intensities for mouse proteins. These low but non-zero signals may arise from several distinct and anticipated sources that originate at the pre-MS and MS acquisition stages. To report ID counts specific to disseminated and primary tumor cells, we were motivated to address these sources, which led us to apply mouse-peptide exclusion exclusively in the PDX samples. Ultimately, the exclusion reduces the number of human proteins and eliminates the murine proteins.

Reviewer Figure 1

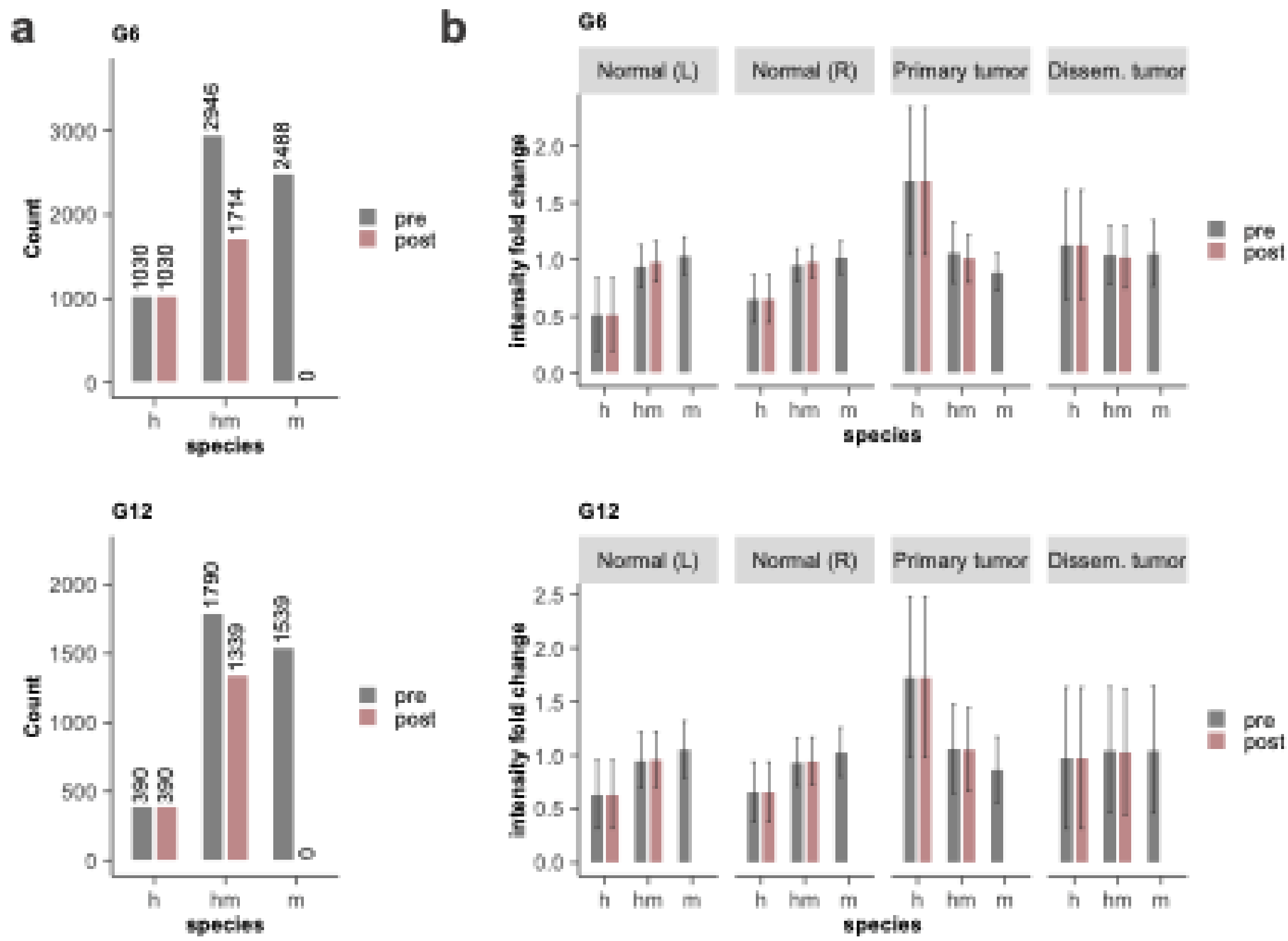

Reviewer #3 (Remarks to the Author): cancer proteomics and phophoproteomics, kinase signaling networks, bioinformatics

Summary

In this study, Ahn et al. have developed an innovative approach to perform phosphoproteomics on subsets of cell types from tissue samples. The method involves fixing cells prior to dissociation, followed by cell sorting and sample preparation for mass spectrometry-based proteomics and phosphoproteomics. This method addresses key challenges in comparing phosphoproteomes across cell types, especially when cells are disseminated. The authors present their optimisation of their method, such as testing different fixatives. However, the approach does cause some changes to the proteome and phosphoproteome. Including analyses of these altered sites and proteins would help future users to understand the trade-offs in using this method, and guide future improvements to the approach. This could be achieved through additional bioinformatics analyses.
As proof-of-principle, the authors applied their method to compare oligodendrocyte lineage cells and non-oligodendrocyte lineage cells from mouse brain tissue. The main application was comparing disseminated and primary glioblastoma cells in two different xenograft models of glioblastoma in mice (either EGFR amplification or activating mutation). This application highlights the strength of the method, which enables purification of disseminated cell types. The authors identified altered phosphosites in disseminated compared to primary tumor cells, including GluA2 Y876 phosphorylation, which they experimentally validated as impacting invasion phenotypes in LN229 cells. The regulatory kinase is listed as Src, but this is based off predictions, which may be complemented through complementary analyses and clear reporting, as the kinase may also be Fyn. With added bioinformatic analyses and transparent reporting of potential limitations of the method, this work could benefit many readers interested in cell type-specific phosphoproteomics.

Major comments

1. Lines 109-110 state that "fixation methods preserved both the phosphoproteome and the whole proteome (Fig. 1b)." This claim is a little strong. Fixation altered 12% of the measured proteome (~503 proteins), 7% of the measured pS/T phosphoproteome (~559 phosphopeptides) and 11% of the pY phosphoproteome (~122 phosphopeptides) (Fig. 1b). This difference isn't a major concern, as trade-offs are common in sample preparation methods. Because this method enables analyses that aren't possible with other approaches, the trade-off could likely be worthwhile for certain applications. A key addition that would strengthen the manuscript is:
    A. A transparent presentation of this difference in the results section.

       Following the reviewer's recommendation, we have updated the text as follows: "The 37°C incubation, common in single-cell tissue studies, significantly altered protein expression and phosphorylation compared to the control, whereas fixation methods, required for preserving physiological signaling, were associated with distinct, smaller magnitude changes in both the phosphoproteome and the whole proteome."

    B. Analyses that would give researchers considering this approach the information they need about the trade-off involved and how they might address the method's current limitations through future adaptations. For example, I'd like to get a sense of the kinds of proteins that are lost (since this shift towards only losing proteins is an important technical artefact), in a more in-depth look than the current analysis comparing peptide length. For example, what gene ontology cellular components are they annotated as? Are they enriched in extracellular proteins? Similarly for the phosphosites, are they dominated by known and predicted substrates of certain kinases or phosphatases?

       We thank the reviewer for these helpful comments. We have now included:
       - **Extended Data Figure 1b**: We performed GO term enrichment analysis to assess the biological processes associated with proteins lost under the conditions tested in Extended Data Fig. 1a, including fixation. Across all conditions, the depleted proteins were consistently enriched for metabolic processes. We have updated the text to explicitly note this observation: "Across all conditions, GO terms associated with metabolic processes were altered compared to control (Extended Data Fig. 1b)."
       - **Extended Data Figure 2c**: We assessed the subcellular localization of proteins depleted following fixation and dissociation of mouse brain tissue using INSIGHT, to determine whether specific compartments, such as extracellular proteins, were preferentially lost. We observed the depletion of proteins associated with cell projections, vesicular proteins, cytoplasmic proteins, and the enrichment of nuclear, endoplasmic reticulum, and mitochondrial proteins. These depletions likely originate from the sectioning step that allows quick fixation and the partial permeabilization by Cytofast. The Result section was updated as follows: "processing led to the depletion of cell projection, vesicular, and cytoplasmic proteins, likely due to sectioning and plasma membrane permeabilization by Cytofast, with the accompanying enrichment of nuclear, plasma membrane, endoplasmic reticulum, and mitochondrial proteins (Extended Data Fig. 2c)." The Methods section was updated as follows: "To assess the subcellular localization of proteins that are altered under various experimental conditions, subcellular

localization of each protein was retrieved from Uniprot and collapsed into high-level GO cellular component parent categories."

- **Supplementary Table 4**: To provide readers with the clear view of how predicted kinase activities change, we compiled a list of kinases whose substrates are predicted by Kinase Library to be depleted under the conditions tested (including fixation) in Extended Data Figure 1a. This Table is now referenced in the revised text: "The 37°C incubation, common in single-cell tissue studies, significantly altered protein expression and phosphorylation compared to the control, whereas fixation methods mitigated many of these changes in both the phosphoproteome and the whole proteome (Fig. 1b, Supplementary Table 4)."
- **Extended Data Figure 2d** and **Supplementary Table 5**: We list the kinases whose substrates are inferred by Kinase Library to be depleted after the dissociation of mouse brain tissue using INSIGHT. This analysis demonstrates preferential loss of some predicted CMGC and CAMK kinase activities, which regulate cell cycle, stress responsiveness, energy sensing, and MAPK signaling, accompanied by relative enrichment of AGC and a subset of CMGC kinases associated with survival signaling, apoptotic regulation, protein synthesis, and metabolic adaptation. We hypothesize that the observed changes are at least partially attributable to the loss of neurons and their long and extensive neuritic extensions during tissue sectioning and dissociation. Neuronal compartments are enriched for CaMK kinases (PMID: 18817731), and their preferential depletion would be expected to affect these kinase groups.

C. It would also strengthen the manuscript to flag proteins and phosphosites altered by fixation that were regulated in the xenograft experiments, ideally in a supplementary table. It would also be helpful to specify if this applies to any hits mentioned in the results. Given that all samples in these xenograft experiments were fixed and sorted, it could have a consistent effect and not be a problem. However, since it hasn't been established how fixation-induced alterations may vary across cell types, it would be worth noting.

   We have updated **Supplementary Table 2** with an additional column called "Altered.by.fixation" which denotes whether proteins and phosphopeptides were altered or unaltered by fixation, as determined by comparison of fixed and unfixed mouse brain control samples. Proteins and phosphopeptides that were only detected in the xenograft experiments were marked as "unknown" under the "Altered.by.fixation" column. The Methods section was updated as follows: "In Supplementary Table 2, the Altered.by.fixation column denotes whether proteins and phosphopeptides were altered or unaltered by fixation, as determined by comparison of fixed and unfixed mouse brain control samples. Proteins and phosphopeptides detected exclusively in the xenograft experiments were annotated as unknown."

2. Lines 312-318 report that G6 increases GRIA2/GluA2-pY876, whereas G12 increases GRIA1/GluA1. However, in Fig. 4e, it appears that GRIA2 total protein levels are not regulated in either model, and GRIA2 Y876 is only quantified in G6, not G12. This highlights a broader issue: differences between G6 and G12 may arise from a lack of quantification rather than true regulation. Since G6 and G12 samples are measured in separate TMT batches (Fig. 1a), cross-batch comparisons could be impacted by missing values due to stochastic quantification. Clarifying whether differences in the significant hits in each model reflect true regulation or missing data would help readers interpret these results more confidently. The same clarification would be

valuable for ion channels. How many of the ion channels listed as regulated in G12 were quantified in G6 but not regulated, rather than not quantified at all?

We thank the reviewer for raising this important point. We agree that some of the observed differences between G6 and G12 are attributable to batch effects and missing values inherent to data dependent acquisition in mass spectrometry-based proteomics. Moreover, because data dependent acquisition preferentially samples more abundant peptides, genuine differences in proteome composition between G6 and G12 can further reduce overlap through differential detectability, making it challenging to disentangle true biological divergence from stochastic missingness and technical effects. Future studies could address these limitations by quantifying samples within the same experimental run, incorporating a shared reference across batches, or employing acquisition strategies that minimize missing values, such as targeted approaches. We have added the following text in the Results section to acknowledge this limitation and clarify its potential impact on the observed differences between the two models: "Differences between G6 and G12 arise from both technical and biological sources. Separate mass spectrometry experiments introduced batch effects and stochastic missing values, while genuine differences in proteome and phosphoproteome compositions further reduced the overlap by altering peptide detectability." Additionally, we have added **Extended Data Figure 7e**, **Extended Data Figure 7f**, and **Supplementary Table 6** to demonstrate the distribution and the overlap of quantified proteins and phosphopeptides between G6 and G12 models, both globally and of ion channels and neurotransmitter receptors, to make clear to readers the nature of the data. Supplementary Data Table 6 summarizes which proteins and phosphopeptides were detected in both G6 and G12, and indicates whether each was significantly regulated in disseminated cells in both models or in only one of the two and whether they are categorized as neurotransmitter receptors or neuronal structures or ion channels (under column "Group"). For example, addressing the reviewer's specific question, of the 72 synaptic structural proteins, calcium channels, and potassium channels queried, 18 were detected in G12; among these, 14 were also detected in G6, whereas 10 were uniquely detected in G6. Importantly, of the 14 detected in both G6 and G12, 7 were not regulated in either with dissemination, and 7 were only upregulated in G12, suggesting that G12 may have preferentially increased the expression of the ion channels and synapse structures in disseminated tumor cells (Supplementary Table 6), as indicated in other data we presented in the manuscript. Furthermore, the data indicate that while shared signaling nodes exist, neurotransmitter receptor and ion channel signaling may be model specific at both the protein and phosphorylation levels.

3. Several biological hypotheses are based on enrichment of predicted kinases, identified from kinase library motif specificies among the regulated phosphosites. These analyses are performed well. While the kinase library is a valuable resource and one effective approach, clarity would be improved by highlighting that these are predicted kinases for the sites, rather than bona fide kinases that have been experimentally validated (e.g., by inhibitor studies and in vitro kinase assays of the entire protein). Because of these, I recommend the following changes to the text, and bioinformatic analyses:
    A. At multiple places in the text (lines 233-239, title in figure of Fig. 3e, line 347, 1264, 1302), clarity of the text could be improved by including the word predicted when referring to kinase-substrate relationships from the kinase library.

        We have updated the text to reflect this recommendation, and they now read as follows:
        - Lines 233-239: "This analysis predicted EGFR as top-ranking tyrosine kinase (Fig. 3d bottom, Extended Data Fig. 4d bottom), consistent with EGFR amplification in both PDX models. Additional serine/threonine kinases were predicted to be enriched

in GBM cells, including G protein-coupled receptor kinases (GRKs) and cyclin-dependent kinases (CDKs)37–40 as well as others that may contribute to GBM progression"

- Lines 346-347: "To identify the potential upstream regulators of the phosphorylation events that are increased in disseminated tumor cells, we performed prediction of kinase-substrate relationships using Kinase Library"
- Line 1264: "Phosphopeptides mapped to the human proteome and enriched in the G6 primary tumors compared to normal cells from the right hemisphere (1.5-fold change, adj. p-val ≤ 0.1) were used to predict active kinases based on the Kinase Library"
- Line 1302: "Active serine, threonine and tyrosine kinases were predicted in primary and disseminated tumor cells using the Kinase library."

B. The predicted kinase enrichment analyses would be strengthened with a separate enrichment analysis of reported substrates (e.g. from the PhosphoSitePlus database), and global analyses of altered regulatory (activating or inhibiting) phosphosites on kinases. These provide crucial orthogonal information to give a more comprehensive view of the likely active kinases. Phosphosites can fit the motifs of many kinases (Johnson et al., 2023, Fig. 3d, https://doi.org/10.1038/s41586-022-05575-3), so these additional lines of evidence can help clarify which of multiple potential active kinases may be regulatory in this case.

We thank the reviewer for this helpful suggestion. To provide orthogonal support for kinase activity inference by Kinase Library, we performed a substrate-based enrichment analysis using curated kinase-substrate relationships from PhosphoSitePlus, as recommended by the reviewer, testing for coordinated shifts in phosphorylation across substrates using rank-based enrichment and added **Extended Data Figure 8c**. The result further support a model in which disseminated G6 tumor cells exhibit reduced activity of cell cycle (CDK2) while increasing MEK/ERK signaling. We did not obtain statistically significant kinase enrichment for G12.

Furthermore, we added **Supplementary Table 7**, which lists all phosphorylated kinases quantified in G6 and G12 primary and disseminated tumor cells, annotated according to reported activating or inhibitory regulatory effects. For example, the phosphorylation of CDKL5 at Y171, which is increased in disseminated G6 cells, has been reported to enhance CDKL5 activity, a kinase implicated in neuronal migration, and may therefore facilitate G6 dissemination. In contrast, disseminated G12 tumor cells exhibit significantly reduced activation loop tyrosine phosphorylation of GSK3A/B relative to primary G12 cells. This shift is expected to relieve repression of beta-catenin/Snail/Slug signaling, promote EMT associated programs, and alter cellular plasticity, collectively providing a plausible mechanistic basis for G12 dissemination. To note this observation, we have updated the Result section as follows: "Disseminated G12 tumor cells exhibited significantly reduced activation loop tyrosine phosphorylation of GSK3A and GSK3B compared to primary G12 cells, indicating attenuation of GSK3 signaling and a shift toward signaling states associated with increased cellular plasticity and invasive potential."

Separately, we want to note that PhosphoSitePlus has suspended academic licensing of its datasets. The kinase-substrate relationships used here were downloaded in 2021. Regulatory phosphorylation site annotations were obtained from SIGNOR in 2025 and integrated with PhosphoSitePlus data downloaded in 2024.

C. Point 3B is particularly relevant to the finding of Y876-GluA2 phosphorylation, which is described as potentially being regulated by Src. The Log2(Score) in kinase library for phosphorylated Y876-GluA2 is 3.2 for Src and 3.0 for Fyn, suggesting that either kinase could be regulatory. It was helpful to supplement the results with the mRNA and protein levels for the potential regulatory kinases in this Fig. 5c. However, there are detectable levels of both Src and Fyn in the cells, and the relative level of a kinase does not often track with activity in the way that regulation of the activating or inhibitory sites do. Expanding this analysis to compare the abundances of any regulatory phosphosites on these kinases would help disentangle this further. In addition to this analysis, Fig. 5i should be edited so that the line between SRC and GluA2-pY876 is dashed to clarify that this kinase-substrate relationship is predicted, and hasn't been experimentally validated. Given the current evidence, it would be worth including Src/Fyn into the image, as the evidence does not clearly indicate only one of these is the regulatory kinase.

   The additional analysis done in point 3B (Supplementary Table 7) revealed that the phosphorylation of the conserved Src-family kinase activation loop site (Y416 in Src, Y420 in Fyn), a modification associated with increased kinase activity, was significantly reduced in disseminated tumor cells relative to primary tumor cells in the G6 model. This phosphorylation site is conserved across multiple Src-family members, and the corresponding phosphopeptide unfortunately could not be unambiguously assigned to a single kinase, precluding resolution of which specific family member(s) contribute to this effect. We have updated Figure 5i (now **Figure 5l**) to clarify that the Src/Fyn-GluA2-pY876 relationship is predicted and not certain in our context.

4. In line 340, it mentions that pY187-ERK2 increased in G6 and was undetected in G12. Why is MAPK1 Y187 presented in the G12 heatmap in Extended data Fig 9a if it wasn't detected in G12?

   We had written the sentence with the intention to indicate that T185/Y187-ERK2 was not detected in G12. Indeed, pY187-ERK2 was detected and increased in both G6 and G12, as indicated in Extended Data Figure 9a (G12) and 9c (G6). We have clarified the text, which now reads: "Furthermore, in G6, total protein and doubly phosphorylated T185/Y187-ERK2 remained unchanged between primary and disseminated tumors (T185/Y187-ERK2 was undetected in G12) while pY187-ERK2 increased in both G6 and G12."

5. Lines 450-452: "We also highlight that signaling circuitries of neuronal and non-neuronal programs interconnect during GBM dissemination by uncovering numerous phosphoproteins, proteins, and kinases and their relationships within those circuitries." This line should be toned down to more accurately reflect that the potential regulatory kinases were predicted, and would require future experimental validation.

   We have updated the text as follows: "We also highlight that signaling circuitries of neuronal and non-neuronal programs may potentially interconnect during GBM dissemination by predicting numerous phosphoproteins, proteins, and kinases to have relationships within those circuitries, which remain to be validated in the future."

Minor comments

1. It would be helpful to mention in the title and abstract that the work is done on xenografts in mice

During revision, we added INSIGHT analyses of human patient samples and murine tissues. The abstract now clarifies that newly added human patient samples (Extended Data Fig. 3a–d) were analyzed alongside glioblastoma patient-derived xenografts implanted in mice and other mouse tissues (Extended Data Fig. 2e), with dissemination examined specifically in the xenograft model in vivo.

2. Line 56-57: to supplement the comment that "phosphoproteomics requires rapid sample processing, which restricts the analysis to bulk tissues", it would be helpful to mention the recent development of deep visual phosphoproteomics, in which phosphoproteomes of laser microdissected cell populations from frozen mouse brain tissue sections were measured (Oliinyk et al., 2025, https://doi.org/10.1101/2025.05.29.656770).

   This preprint, posted in June 2025 after our December 2024 submission to the Nature Publishing Group and bioRxiv, is now cited in the revised manuscript's Discussion section. These complementary technical developments underscore a rapidly evolving phosphoproteomics landscape, in which increasingly sensitive and spatially resolved approaches are enabling deeper interrogation of cell-type-specific signaling in complex tissues. We are excited that INSIGHT contributes to this broader methodological trajectory.

3. Figure 1J: The Log2FC color scale isn't centred at 0, so 0 Log2FCs appear as blue in the graph. Please regenerate the plot with a centred color gradient, so that 0 is the middle grey color.

   We have updated the figure as recommended.

4. Line 212-214: When the authors state "we performed differential expression analysis of the phosphoproteome and proteome between primary tumor cells and normal cells, a task that has been previously impossible with tissue phosphoproteomics". This statement may be more accurate if it referred to measure diffuse, invasive cells, rather than primary tumor cells. The CPTAC consortium have compared primary tumor cells to normal cells in multiple tissue types including comparing high-grade gliomas to normal brain tissue (e.g. Liu et al., 2024, https://doi.org/10.1016/j.ccell.2024.06.004)

   We revised the text to clarify that our differential expression analysis compared normal cells and primary tumor cells originating from the same tissue, where both populations are spatially intermixed. In contrast, the CPTAC analysis compared the phosphoproteome of normal and tumor samples derived from distinct, unmatched tissues. The text now reads: "we performed differential expression analysis of the phosphoproteome and proteome between primary tumor cells and normal cells that were spatially intermixed within the same tissue, a task that has been previously impossible with tissue phosphoproteomics."

5. Line 258, 285, 311, 399, 1182, 1298, 1300, 1445, 1476, 1499: The manuscript frequently refers to the "expression" of phosphopeptides. Given that "expression" generally describes the synthesis of mRNA or a protein, this usage may be misleading. Phosphopeptide levels are influenced by both phosphorylation and dephosphorylation, covering more processes than protein synthesis. I recommend revising the terminology to "abundance" or "levels" to improve accuracy.

   We have updated our manuscript as recommended by the reviewer and read as follows:
   Line 258: "Reductions in EGFR and pT693-EGFR levels…"
   Line 285: "We therefore examined the systems-level abundance changes…"
   Line 311: "INSIGHT dataset revealed a statistically significant increase in the abundance…"

Line 1182: “(b) Differential abundance of phosphopeptides (pY, pS, and pT) and proteins…”
Line 1298: “(f) Relative protein and phosphoprotein levels of ion channels between…”
Line 1445: “Extended Data Figure 4. Phosphoproteome and protein abundance…”
Line 1476: “Extended Data Figure 5. Levels of EGFR and pT693-EGFR in G6 PDX tumor cells.”
Line 1499: “Extended Data Figure 6. Levels of EGFR and pT693-EGFR in G12 PDX tumor cells.”

6. It would be helpful if the methods section specified the threshold of the site percentile or Log2(Score) used from kinase library to create the sets of predicted substrates that were analysed in enrichment analysis.

   We have added the following text to the method: “For each kinase, only substrates ranked 1 (i.e., the highest-confidence predicted substrates based on percentile rank) were included in the enrichment analysis.”

7. Figure 4d: It could be helpful to specify that this enrichment was done on the proteome abundance ranks rather than mRNA levels, as it isn’t explicitly mentioned in the results text or the figure legend, but the results currently mention a “gene signature” and “top-ranked genes”, so it could be a place for confusion.

   We thank the reviewer for helping us be clear. We have updated the legend which now reads: “(d) GSEA plots for the gene set "GOBP Regulation of postsynaptic membrane potential" comparing disseminated versus primary tumor cells using their proteome abundance.”

8. The article often describes results as “significantly increased” (line 334, 363) or “showed a downward trend” (line 251) without sharing the fold-changes or p-values in the text. Including these would help readers interpret the findings.

   We appreciate the reviewer for this comment. We initially included all fold-changes and p-value comparisons in the main text. However, this caused the manuscript to significantly exceed the journal’s word limit, restricting other essential interpretations and discussions. To address this, we had decided to display the fold-change values directly within each figure plot and indicate the corresponding p-values, ensuring completeness while maintaining conciseness. We hope this approach is acceptable and effectively balances interpretability, readability and editorial requirement.
