# Supplementary material for: Uncovering the signaling networks of disseminated glioblastoma cells in vivo with INSIGHT
Source: Nat Commun. 2026 Aug 8;17:9536. doi: 10.1038/s41467-026-76587-0 (PMC13547291; doi:10.1038/s41467-026-76587-0)
Supplement: Supplementary file 3 [file 41467_2026_76587_MOESM1_ESM.pdf]

Supplementary Information for

Uncovering the signaling networks of disseminated glioblastoma cells *in vivo* with INSIGHT

Author list

Ryuhjin Ahn*[1,2], Alicia D. D'Souza[1,3], Lawrence Long[2], Yufei Cui[1,2], Jennifer Gantchev[4], Gerard Baquer[4,5], Danielle Burgenske[6], Katrina K. Bakken[6], Lauren L. Ott[6], Brett L. Carlson[6], Grace Zhou[7], Ishwar N. Kohale[1,2], Charles A. Whittaker[1,8], Heidi Temple[1], Tomer M. Yaron-Barir[9], Jeffrey Wyckoff[1,10], Terry C. Burns[6], Rachael A. Vaubel[6], Cameron T. Flower[1,11], Wei Huang[12], Ann Tuma[6], Jared L. Johnson[13,14], Nathalie Y R Agar[15,16], Josie Ursini-Siegel[17,18], Jann Sarkaria[6], and Forest M. White*[1,2,11].

Affiliations

[1] David H. Koch Institute for Integrative Cancer Research, Massachusetts Institute of Technology (MIT), Cambridge, MA, 02139, USA.
[2] Department of Biological Engineering, MIT, Cambridge, MA, 02139, USA.
[3] MIT-Harvard Health Sciences and Technology, Cambridge, Massachusetts, 02139, USA.
[4] Department of Neurosurgery, Brigham and Women's Hospital, Harvard Medical School, Boston, MA, USA.
[5] Department of Electronic Engineering, Rovira i Virgili University, Tarragona, Spain.
[6] Mayo Clinic, Rochester, MN, 55905, USA.
[7] MIT, Cambridge, MA, 02139, USA.a
[8] Barbara K. Ostrom (1978) Bioinformatics and Computing Core Facility of the Swanson Biotechnology Center, David H. Koch Institute for Integrative Cancer Research
[9] Columbia University Vagelos College of Physicians and Surgeons, New York, NY 10032, USA
[10] Microscopy Core Facility of the Swanson Biotechnology Center, David H. Koch Institute for Integrative Cancer Research
[11] Program in Computational and Systems Biology, MIT, Cambridge, MA, 02139, USA.
[12] Preclinical Imaging and Testing Facility of the Swanson Biotechnology Center, David H. Koch Institute for Integrative Cancer Research, MIT, Cambridge, MA 02139, USA.
[13] Department of Cell Biology, Harvard Medical School, Boston, MA 02115, USA.
[14] Dana-Farber Cancer Institute, Harvard Medical School, Boston, MA 02215, USA.
[15] Department of Radiology, Brigham and Women's Hospital, Harvard Medical School, Boston, MA, USA.
[16] Department of Cancer Biology, Dana-Farber Cancer Institute, Boston, MA, USA.
[17] Division of Experimental Medicine, McGill University, Montreal, Quebec, Canada.
[18] Lady Davis Institute for Medical Research, Jewish General Hospital, Montreal, Quebec, Canada.

*To whom correspondence should be addressed:
Forest M. White, Ph.D., at fwhite@mit.edu and Ryuhjin Ahn, Ph.D., at ryuhjin.ahn@mcgill.ca

This file includes:

Supplementary Figure 1 to Supplementary Figure 11

Supplementary Figure 1. Optimization of fixation method to preserve the phosphoproteome.

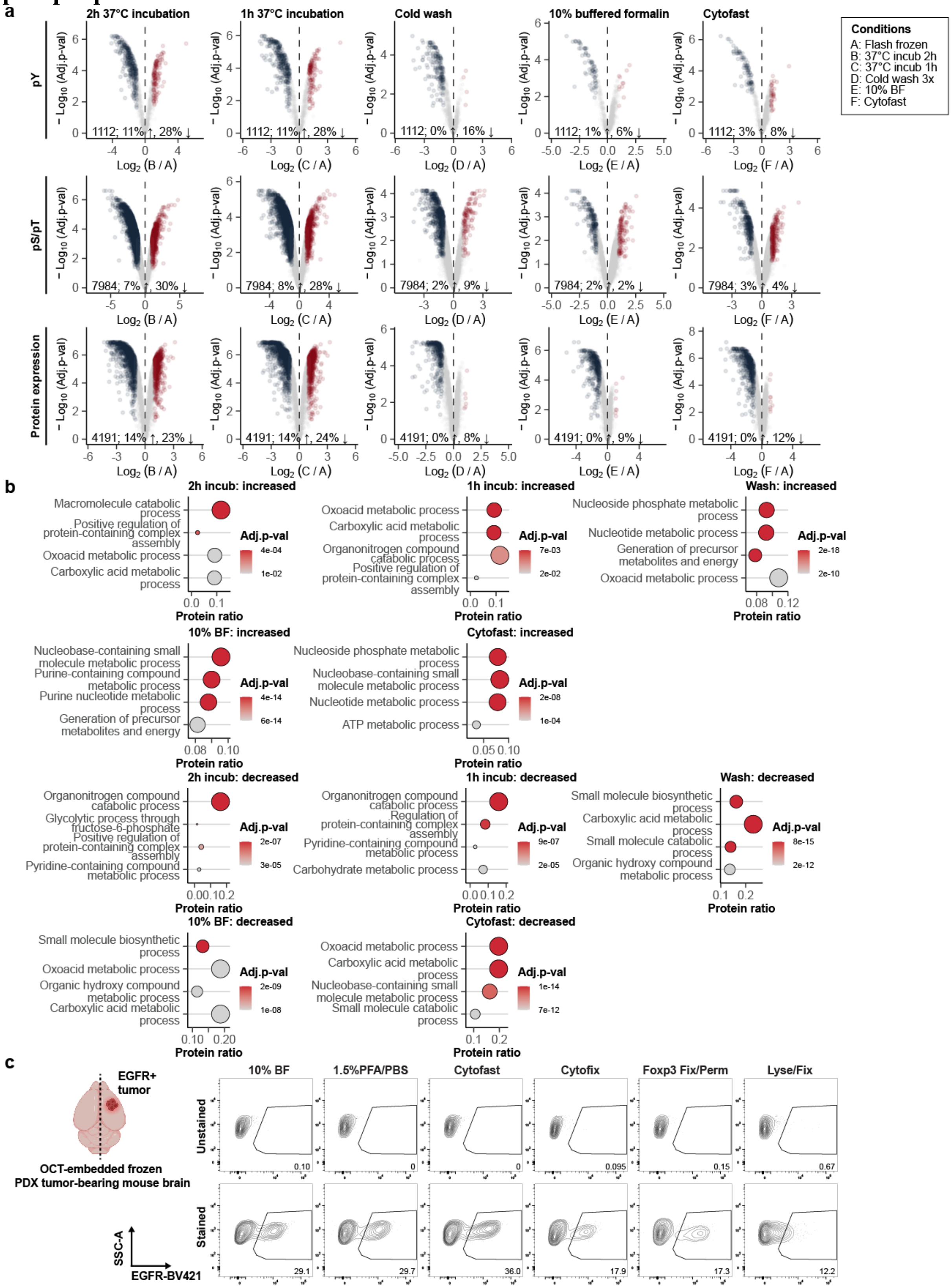

(a) Volcano plots depict the differential expression of phosphopeptides containing pY, pS, and pT as well as proteins by MS with indicated conditions (incubation at 37°C for 1 or 2 hours, PBS ice cold wash (3x) without fixative, 10% buffered formalin, and Cytofast) compared to the flash-frozen mouse brain tissues. The number of total identifications and the percentage of up- and down-regulated identifications (fold-change ≥ 2, adjusted p-value ≤ 0.05) are shown in the left bottom corner.

(b) GO term enrichment analysis of up and down-regulated proteins under indicated conditions compared to control in (a).

(c) EGFR-amplified GBM PDX was injected into the right hemisphere of a mouse. Brain was removed, flash frozen in OCT compound, cut down the mid-sagittal plane, and each hemisphere was cryosectioned, and the sections were fixed with various fixatives as indicated before being dissociated for flow cytometry analysis. Representative flow plots of EGFR are shown for the following fixatives: 10% buffered formalin (BF), 1.5% paraformaldehyde, Cytofast (Biolegend), Cytofix (BD Biosciences), Foxp3 fix/perm buffer (Biolegend), and Phosflow lyse/fix buffer (BD Biosciences). The percentage of cells shown in the bottom right. Part of the figure was made using BioRender.

Supplementary Figure 2. Phosphoproteome and proteins remain intact after the INSIGHT workflow allowing for antibody or fluorescent endogenous protein-based cell isolation.

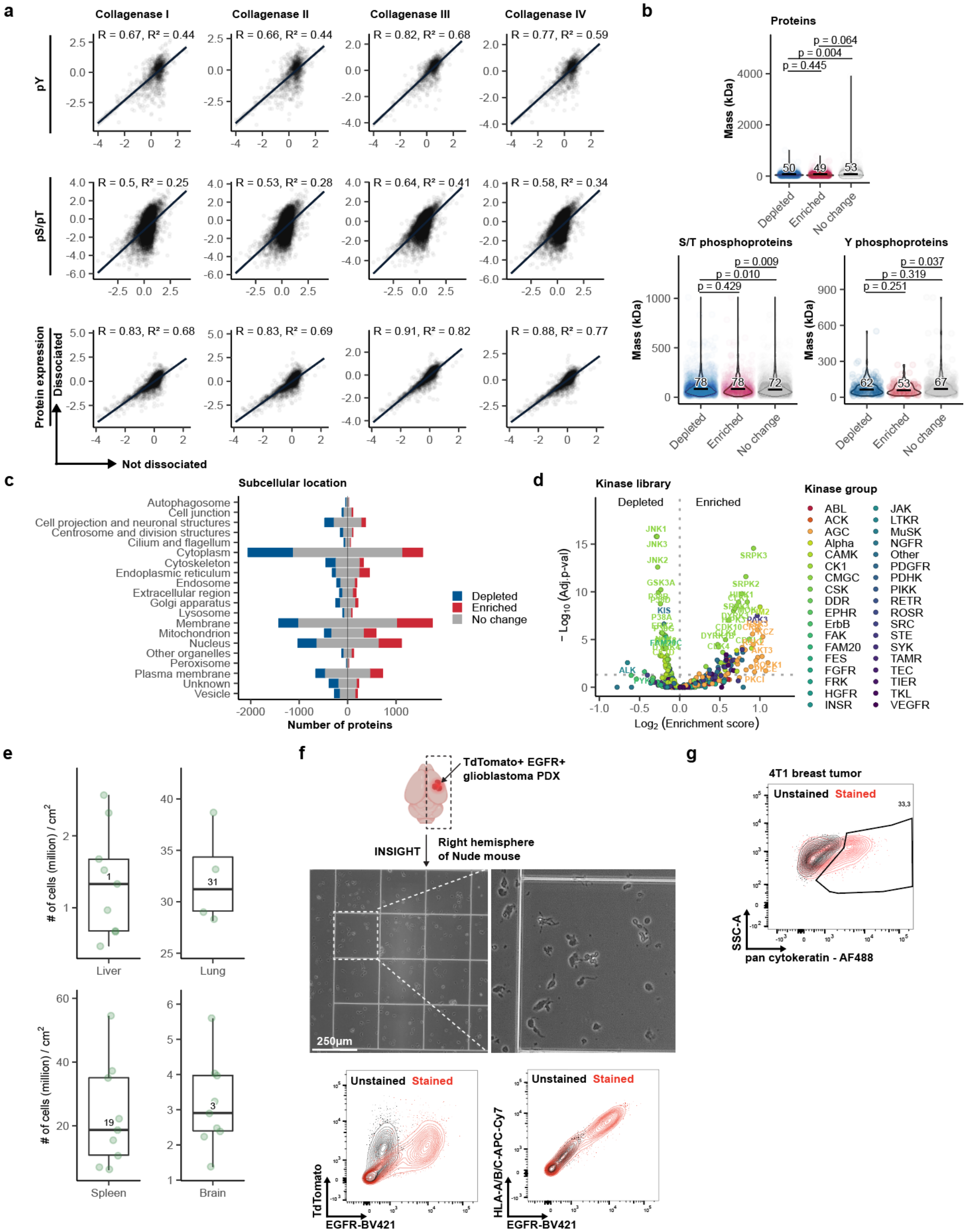

(a) Proteome and phosphoproteome quantified by MS after dissociation with collagenases I, II, III, and IV were compared to that of fixed but not dissociated controls. X and Y axes indicate log-transformed mean-normalized fold changes for each ID. R = Pearson correlation coefficient. R^2 = coefficient of determination.

(b) Molecular weights (kilodaltons; kDa) of proteins and phosphoproteins that are enriched or depleted following fixation and dissociation, compared to the control that was fixed without dissociation in **a**. Median values are shown in each violin plot. The p-values were calculated by pair-wise t-test.

(c) Subcellular location of the proteins that are depleted and enriched with INSIGHT on mouse brain tissue

(d) Kinases whose substrates are predicted by Kinase Library to be depleted and enriched with INSIGHT on mouse brain tissue

(e) Number of cells per cm^2 retrieved from applying INSIGHT on the liver, lung, spleen, and brain of mice. The area was calculated based on the cross-sectional area of the tissue measured during cryosection.

(f) TdTomato+ EGFR+ GBM PDX implanted in the right hemisphere of a mouse was analyzed using INSIGHT. Brightfield images of the cells dissociated are shown. Scale bar = 250 µm. Part of the figure was made using BioRender. (bottom) Flow cytometry contour plots displaying cells positive for TdTomato, EGFR and HLA-A/B/C under unstained (black) and stained (red) conditions using cells isolated.

(g) Orthotopically injected 4T1 breast tumor was fixed, dissociated, and permeabilized using INSIGHT, and probed for pan cytokeratin using anti-pan cytokeratin-AF488 antibody.

Supplementary Figure 3. Application of INSIGHT on patient tissue and G6 and G12 PDX tumor-bearing mouse brains.

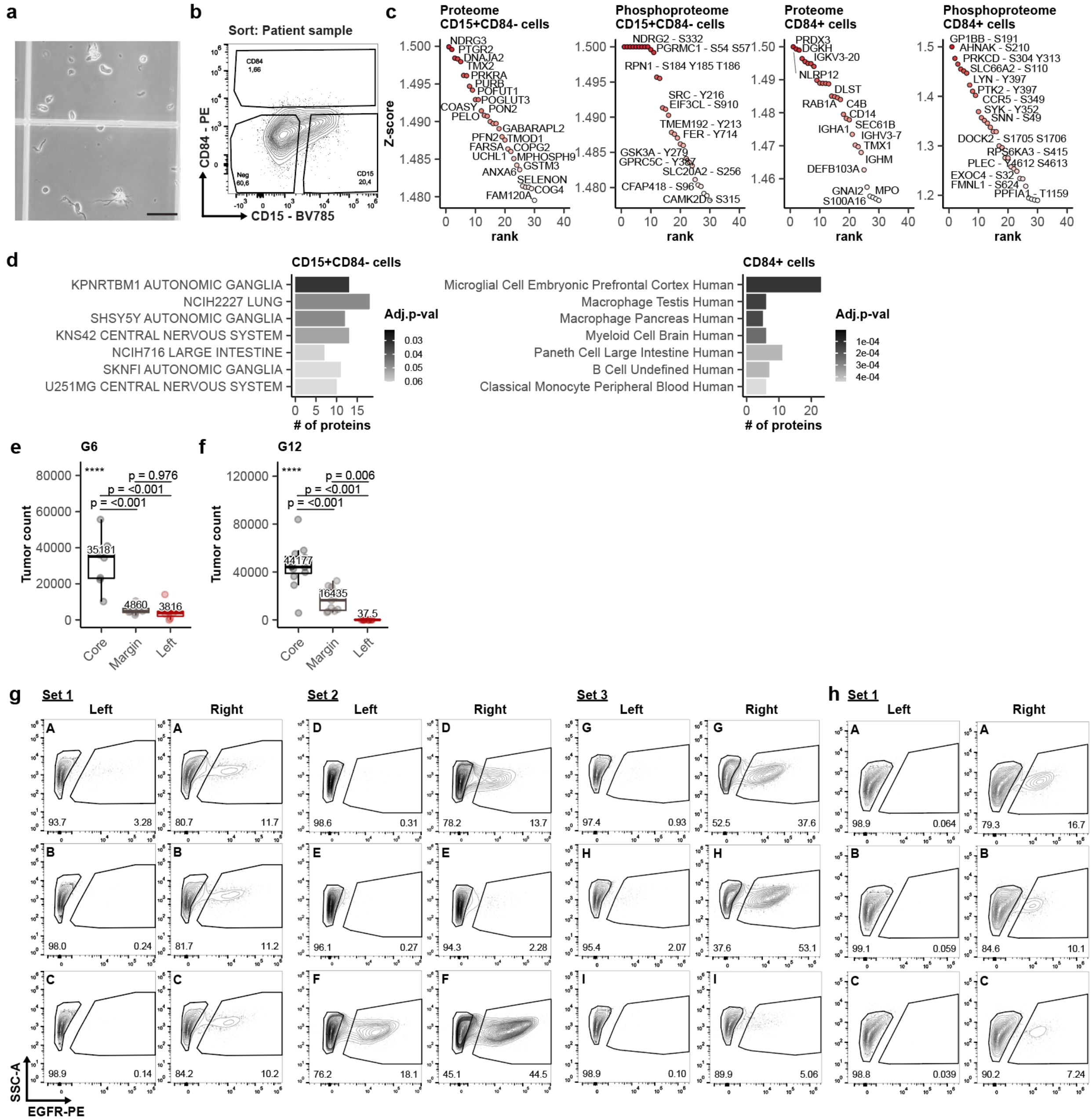

(a) Fixed and dissociated glioblastoma patient tissue using INSIGHT. Scale bar = 50 µm

(b) Gating strategy used to sort the fixed and dissociated patient tissue based on CD15 and CD84 positivity using INSIGHT.

(c) Top most differentially expressed proteins and phosphopeptides in the sorted CD15^{+}CD84^{-} cells and CD84^{+} cells.

(d) Enrichment analysis on the top most differentially expressed protein in CD15^{+}CD84^{-}cells and CD84^{+} cells using Cancer Cell Line Encyclopedia (left) and Cell Marker 2024 database (right), respectively. The analysis indicates that CD15^{+}CD84$^{–}$ cells exhibit expression profiles most

similar to glioblastoma cell lines in the Cancer Cell Line Encyclopedia (927 cancer cell lines), whereas CD84^{+} cells show expression patterns consistent with myeloid cells.

(e-f). Coronal sections of (a) G6 and (b) G12 PDX tumor-bearing mouse brains with the highest primary tumor burden were analyzed for tumor count based on three regions: tumor core, tumor margin, and the left hemisphere. ANOVA p-value (**** p-value < 0.0001) is shown at the top left. Adjusted p-values between conditions shown.

(g-h) Representative FACS profile of (c) G6 and (d) G12 PDX tumor-bearing brains subjected to INSIGHT.

Supplementary Figure 4. Phosphoproteome and protein abundance within primary tumor cells and normal cells from the G12 PDX model.

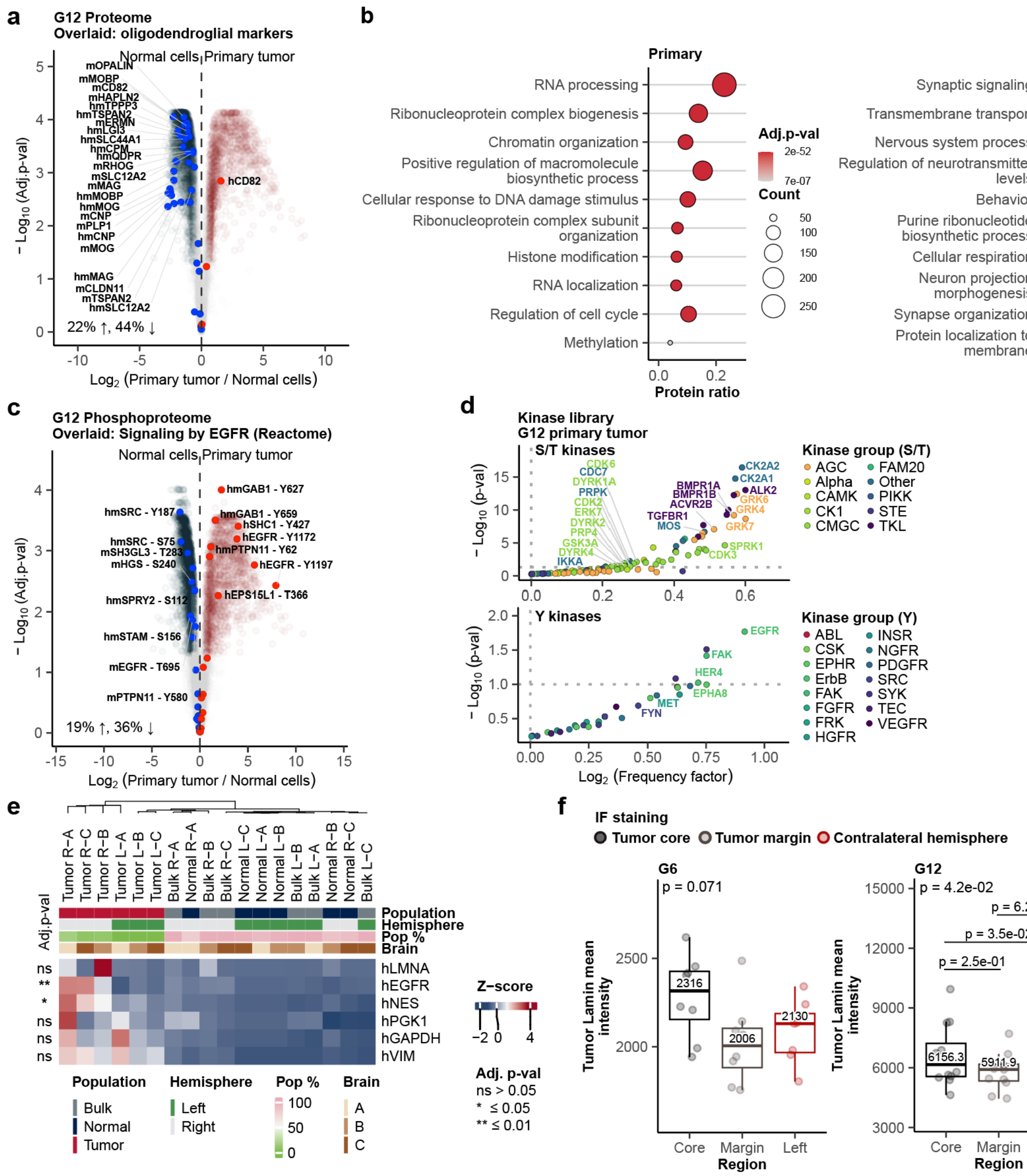

(a) Volcano plot illustrating differentially enriched proteins between EGFR+ primary tumor cells and normal cells from the left hemisphere of the G12 PDX model, with oligodendrocyte markers

overlaid to highlight the distinct separation between the two cell subpopulations. The percentage of significantly differentially expressed IDs (1.5-fold change, adj. p-val ≤ 0.05) is shown in the bottom left corner. Lowercase prefixes indicate proteins specific to human (h), mouse (m), or shared between human and mouse (hm).

(b) Biological processes significantly enriched (adj. p-val ≤ 0.05) in G12 primary tumor cells (left) and normal cells (right) from the right hemisphere based on their protein expression (1.5-fold change, adj. p-val ≤ 0.05). The top annotations are shown.

(c) Volcano plot showing the differentially enriched phosphopeptides in primary tumor cells and normal cells from the right hemisphere of the G12 PDX model. EGFR signaling components based on the Reactome database are labeled. The percentage of significantly differentially expressed IDs (1.5-fold change, adj. p-val ≤ 0.05) is shown in the bottom left corner. Lowercase prefixes indicate proteins specific to humans (h), mice (m), or shared between humans and mice (hm).

(d) Phosphopeptides (containing pY, pS, and pT) mapped to the human proteome and enriched in the G12 primary tumors compared to normal cells from the right hemisphere (1.5-fold change, adj. p-val ≤ 0.1) were used to infer active kinases based on the Kinase Library[4].

(e) Levels of human Lamin A/C (LMNA), Nestin (NES), Vimentin (VIM), and EGFR across bulk cell mixtures, normal cells (EGFR-), and EGFR+ tumor cell populations from the left (L) and right (R) hemispheres of the G12 PDX model by INSIGHT. Olig2 was not detected. GAPDH and PGK1 housekeeping proteins are also shown. Adjusted p-values were calculated using the Benjamini-Hochberg correction.

(f) Lamin A/C levels were quantified in the tumor core, tumor margin, and left hemisphere of G6 and G12 PDX models by immunofluorescence staining. GAPDH and PGK1 are housekeeping proteins. ANOVA p-value shown at the top left. Adjusted p-values between conditions shown.

Supplementary Figure 5. Levels of EGFR and pT693-EGFR in G6 PDX tumor cells.

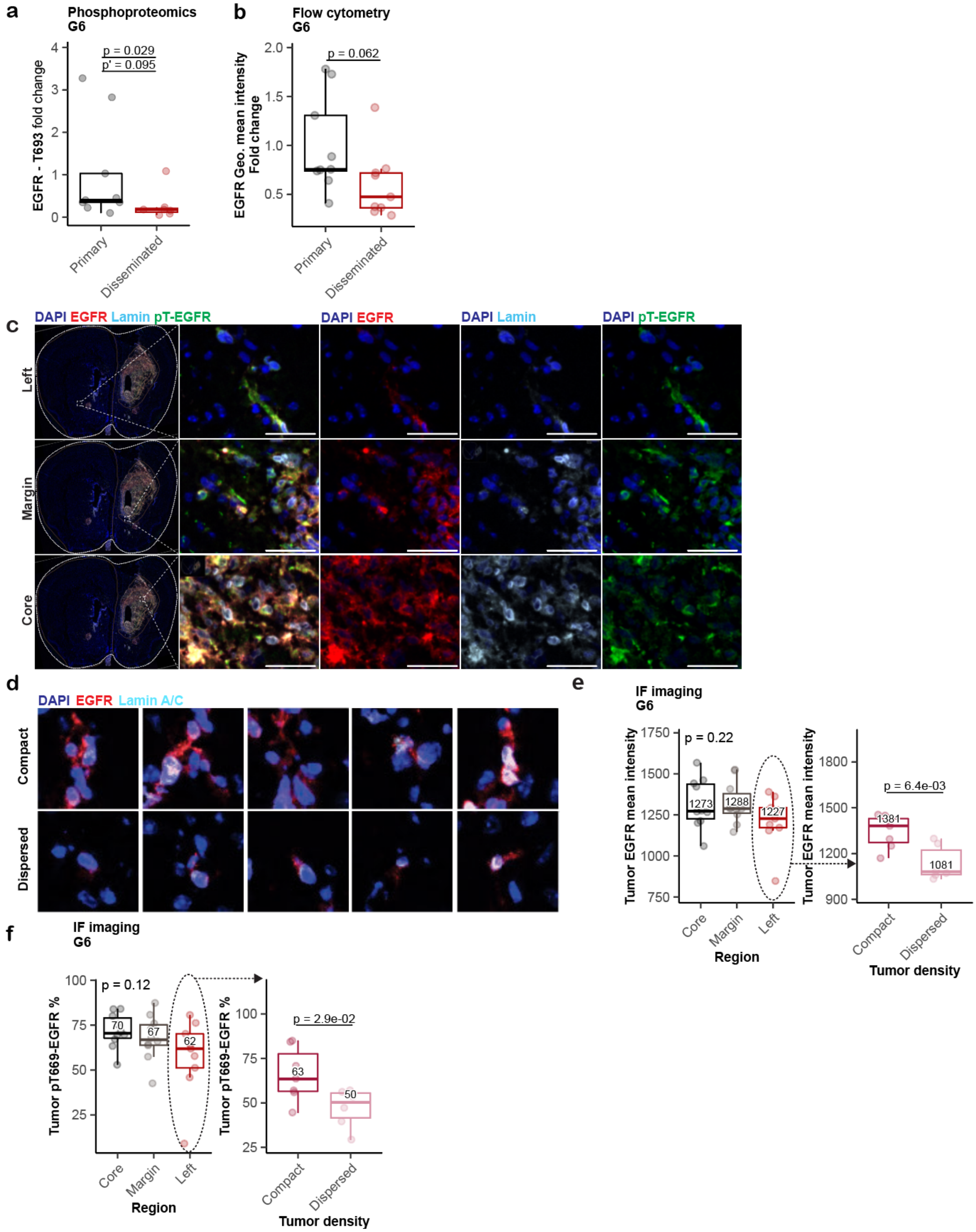

(a) pT693-EGFR fold change between primary and disseminated G6 tumor cells by INSIGHT. Both Benjamini-Hochberg adjusted-p value (p’) and unadjusted p-values (p) are shown.

(b) Geometric mean intensity of EGFR in primary and disseminated G6 tumor cells based on FACS. P-values by student t-test.

(c) G6 PDX tumor-bearing brains were coronally sectioned and stained with anti-pT693-EGFR antibody, human-specific anti-Lamin A/C antibody, and human-specific anti-EGFR antibody and DAPI (nuclei). Shown are representative images. Images in the first column show the whole brain. Dotted lines define the left hemisphere, the primary tumor in the right hemisphere, and tumors at the margin of the primary tumor in the right hemisphere. The box with a white dotted line indicates the location of the four images within the section. Scale bar = 40 μm.

(d) Tumor cells from the left hemisphere of G6 traveling as singlets in a dispersed manner and as groups in a compact manner as observed in samples from **c**.

(e) EGFR mean intensity in tumors quantified by co-IF as in **c**. Compact and dispersed cells in the left hemisphere were further analyzed in the right panel. ANOVA p-value (ns = not significant) is shown at the top left. P-values for the compact versus dispersed cell comparison calculated by Student's t-test.

(f) Percentage of G6 tumor cells that are positive for pT693-EGFR by co-IF as done in **c** and **d**. Cells that are compact or dispersed were further analyzed (right panel)In all co-IF experiments, ANOVA p-value (**** = p-value ≤ 0.0001, *** = p-value ≤ 0.001; ** = p-value ≤ 0.01; * = p-value ≤ 0.05) is shown at the top left. Adjusted p-values shown between conditions.

Supplementary Figure 6. Levels of EGFR and pT693-EGFR in G12 PDX tumor cells.

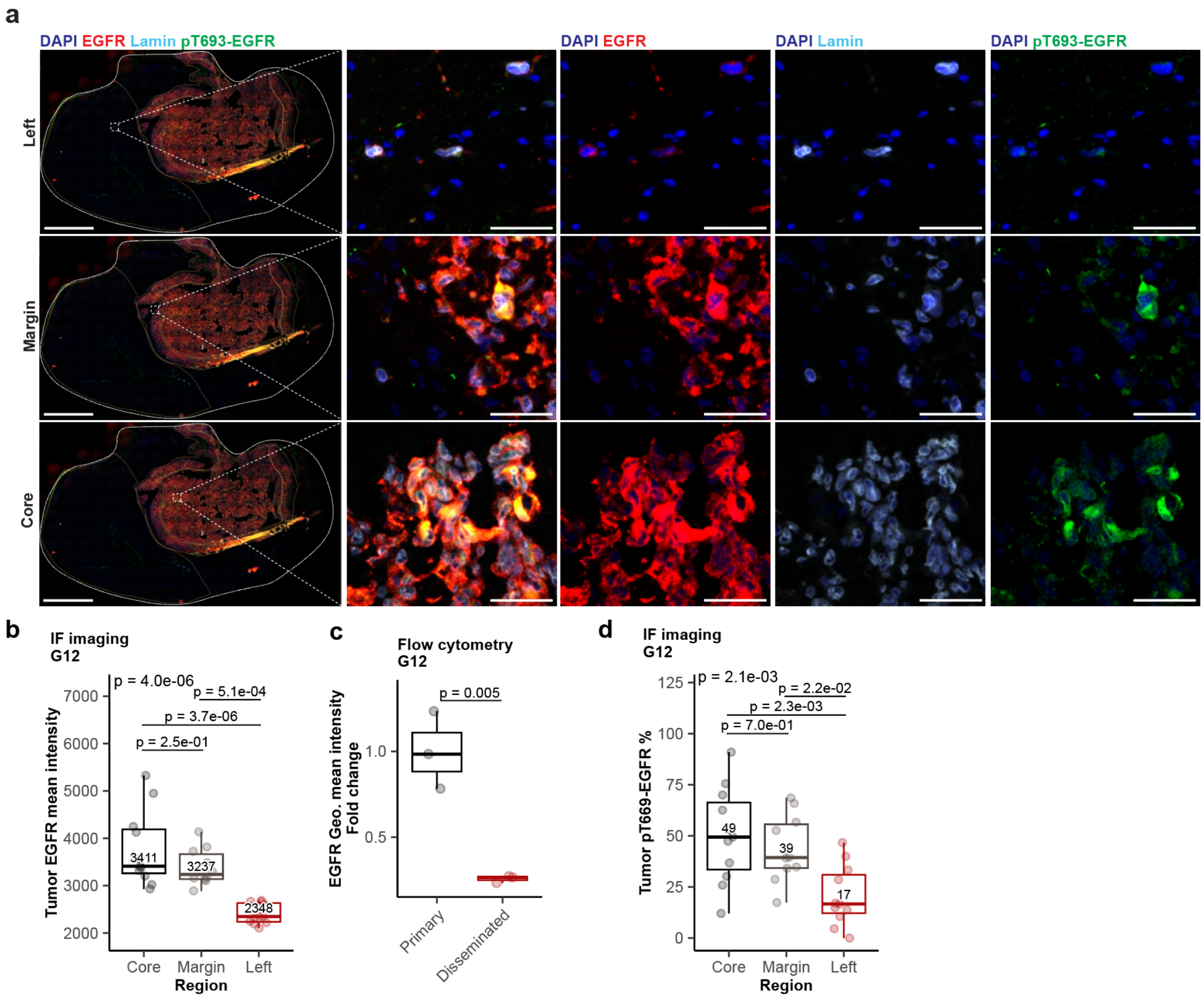

(a) G12 PDX tumor-bearing brains were coronally sectioned and stained with anti-pT693-EGFR antibody, human-specific anti-Lamin A/C antibody, and human-specific anti-EGFR antibody and DAPI (nuclei). Shown are representative images. Images in the first column show the whole brain. Dotted lines define the left hemisphere, the primary tumor in the right hemisphere, and tumors at the margin of the primary tumor in the right hemisphere. The box with a white dotted line indicates the location of the four images within the section. Scale bar = 40 µm for magnified images, 2mm for whole sections.

(b) EGFR mean intensity in tumors quantified by co-IF in **a**.

(c) Geometric mean intensity of EGFR in primary and disseminated tumor cells based on FACS. P-values by student t-test.

(d) Percentage of G12 tumor cells that are positive for pT693-EGFR by co-IF as done in **a**.

In all co-IF experiments, ANOVA p-value is shown at the top left. Adjusted p-values between conditions were determined using Tukey's HSD test.

Supplementary Figure 7. Signalome of disseminated and primary tumor cells *in vivo*.

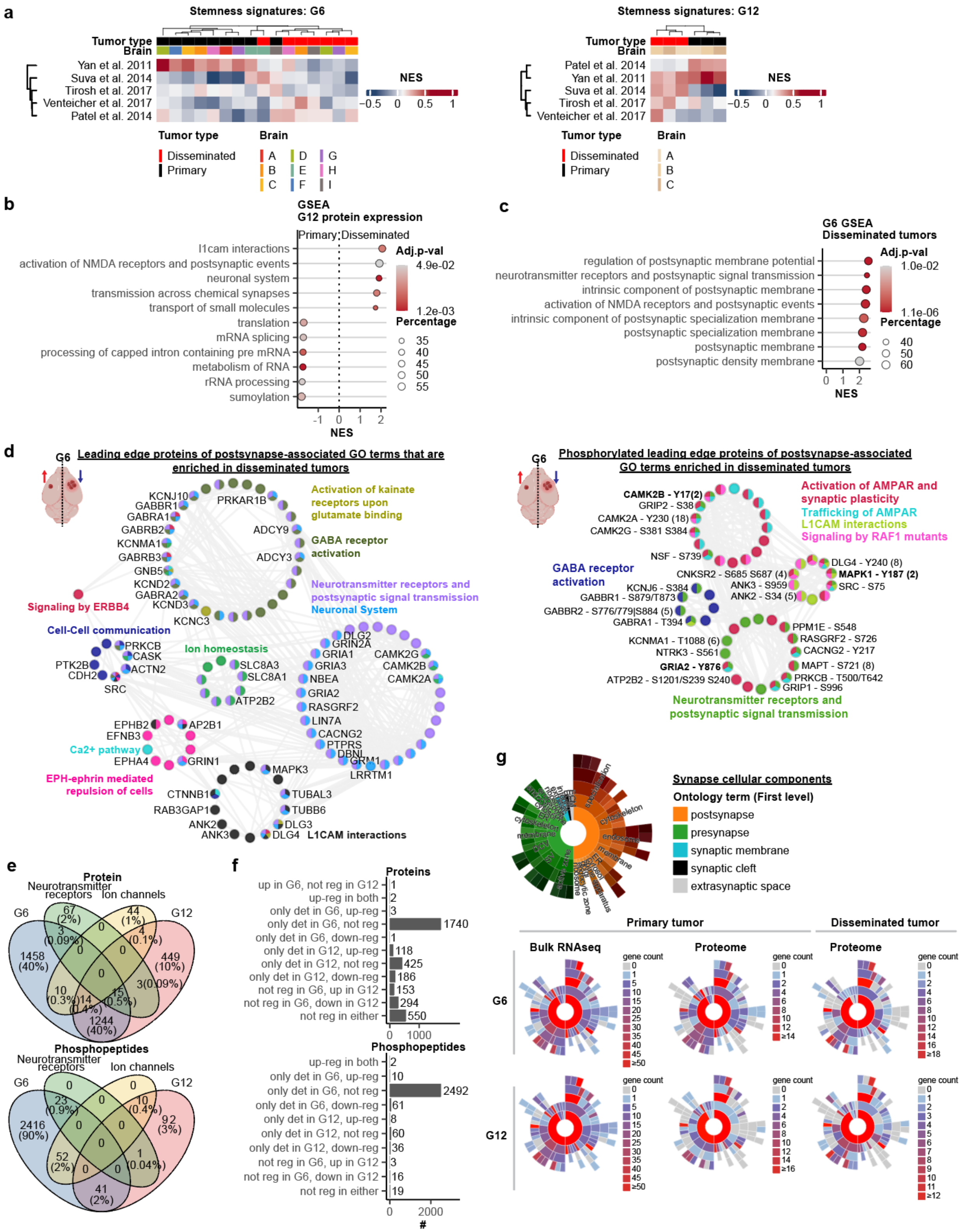

(a) Primary and disseminated tumor cells based on their whole proteome were assessed for the enrichment of glioblastoma and glioma stem-like gene signatures.
(b) Gene Set Enrichment Analysis (GSEA) results on the proteins quantified in the primary and disseminated G12 tumors (log_2 protein expression ratios) based on the Reactome database.
(c) GSEA results on the proteins quantified in the primary and disseminated G6 tumors (log_2 protein expression ratios) across all MSigDB databases and filtered for those associated with postsynaptic signaling with significance (adj. p-val ≤ 0.05)
(d) Pathway enrichment analysis of proteins (left) and phosphoproteins (right) that were increased in disseminated G6 tumors compared to primary G6 tumors (fold change > 1) and were detected as leading-edge proteins in **c**. Part of the figure was made using BioRender.
(e) Four-way Venn diagrams depict the distribution and overlap of detected neurotransmitter receptors and ion channels in G6 and G12 tumors at the protein level (top) and phosphopeptide level (bottom). Numbers indicate counts, with percentages relative to the total detected proteins or phosphopeptides shown in parentheses.
(f) Bar plots summarize proteins (top) and phosphopeptides (bottom) according to their quantification and regulation status across G6 and G12 models. Categories indicate whether features were detected (det) in only one model or both, and whether they were up-regulated (reg), down-regulated, or not significantly regulated in each model. Continued in **Supplementary Table 6**.
(g) Ontology analysis of synaptic components, annotated in SynGO[130], in primary tumor cells using bulk RNAseq, as well as in primary tumor and disseminated cells from INSIGHT datasets. The left sunburst plot illustrates the first-level ontology terms related to synaptic structures. The right panels show the count distribution of transcripts, proteins, and phosphoproteins annotated with each ontology term, using concentric heatmaps.

Supplementary Figure 8. Overlap between synapse and dissemination-associated gene signatures.

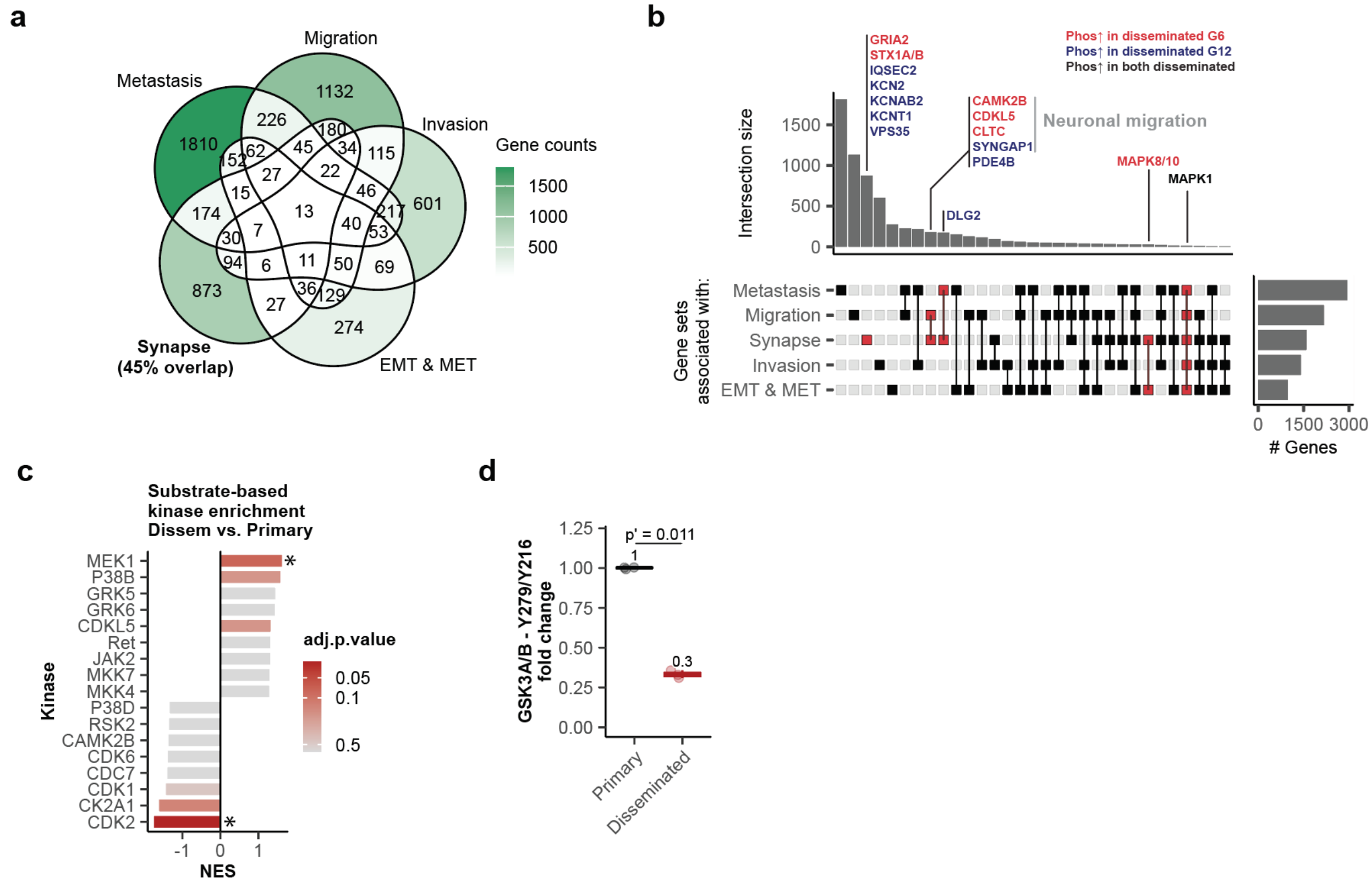

(a) Venn diagram showing the overlap of gene sets associated with shown biological processes. Numbers indicate gene counts within each subset, with color intensity reflecting the size of the gene group (darker green indicates higher counts). Synapse-associated genes showed a 45% overlap with other processes.

(b) UpSet plot showing intersections of gene sets shown in **a** and where differentially activated phosphoproteins belonged. The top bars represent intersection sizes. Genes with increased phosphorylation in disseminated G6 tumors (red), G12 tumors (blue), or both (black) are indicated. Phosphoproteins, including GluA2/GluA2, CaMKIIb, CAMK2B, and MAPK8/10, are highlighted for their role in neuronal migration. The bottom matrix displays combinations of gene sets in each intersection, with red indicating significant overlaps. Right bar chart on the right shows the total gene count for each set.

(c) Substrate based kinase enrichment analysis comparing disseminated and primary G6 tumor cells. Positive normalized enrichment scores (NES) indicates enrichment in disseminated cells, whereas negative NES indicates enrichment in primary cells. Kinases are ordered by NES, and color denotes adjusted p-value. Asterisks indicate kinases passing the significance threshold (0.1).

(d) Box plots show fold changes of total GSK3A/B-pY279/216 in G12 primary (gray) and disseminated (red) tumors as assessed by mass spectrometry. BH-adjusted p-values (p') are shown.

Supplementary Figure 9. Differentially expressed phosphopeptides and proteins in primary and disseminated tumor cells *in vivo*.

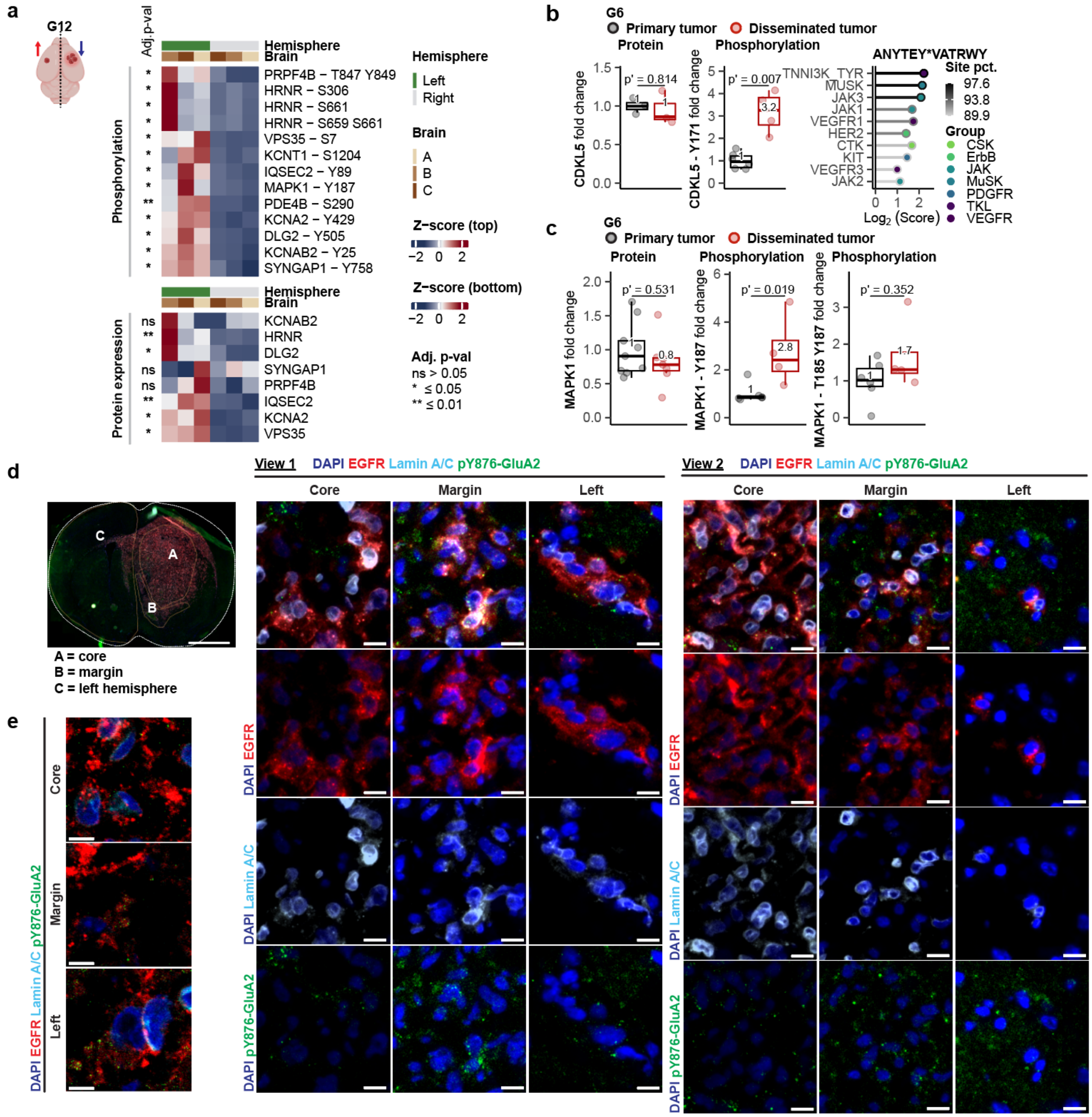

(a) Heatmap depicting the differentially upregulated (fold change ≥ 1.5 and adj. p-val ≤0.05) phosphopeptides (top panel) and their corresponding proteins (bottom panel; shown only if they were detected) in G12 disseminated and primary tumors. BH-corrected adjusted p-values between the primary (Tumor R) and disseminated (Tumor L) are shown on the left side of the heatmap. Z-scores represent normalized expression changes.

(b) CDKL5 total protein expression and phosphorylation (pY171) between primary and disseminated tumors. BH-adjusted p-values (p') are shown. The lollipop plots (right) represent predicted kinases for the matching phosphorylation sites (left) based on the Kinase Library. The phosphopeptide queried is shown at the top of the lollipop plots, with the phosphorylated residue

indicated in red (*). The site percentile (pct.) indicates the percentile score of the phosphopeptide in the phosphoproteome as the substrate for the kinase listed.

(c) Box plots show fold changes of total MAPK1 (ERK2) and pY187-MAPK1, and pT185/Y187-MAPK1 in primary (gray) and disseminated (red) tumors. BH-adjusted p-values (p') are shown.

(d) Co-IF of the coronal section of G6 PDX-tumor bearing brain. The top left panel shows the whole section, with regions of interest labeled: A (tumor core), B (tumor margin), and C (left hemisphere). Staining included DAPI (blue, nuclei), human EGFR (red), human Lamin A/C (cyan), and pY876-GluA2 (green). Representative images are shown. Scale bar =10 µm for magnified images and 2 mm for the whole section.

(e) Confocal imaging of the co-IF done in **d**. Scale bar = 5 µm.

Supplementary Figure 10. Proliferation-associated signaling is suppressed in disseminated tumor cells relative to primary tumor cells of G6 and G12 PDX models.

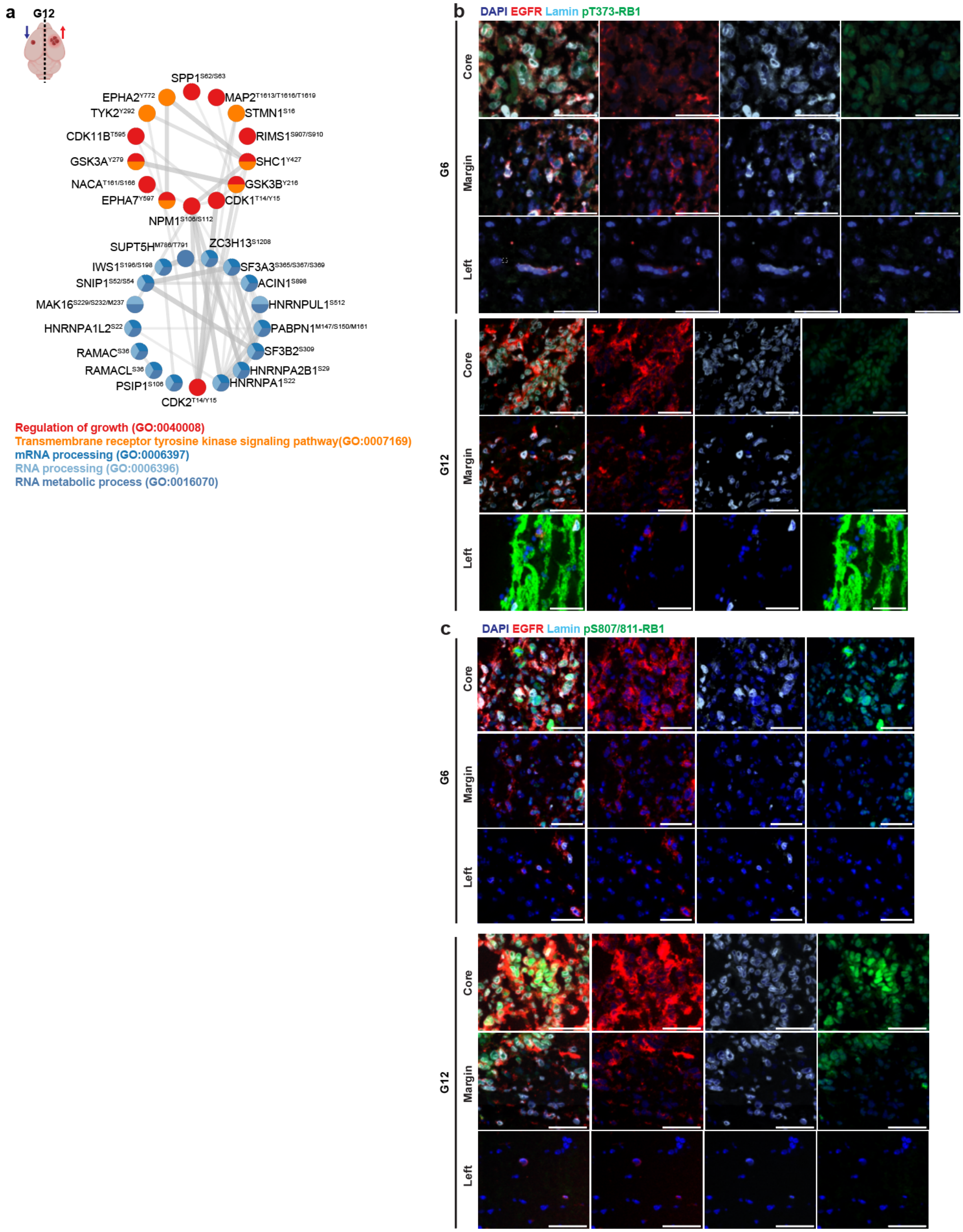

(a) Pathway enrichment analysis of phosphoproteins significantly downregulated (fold-change ≥ 1.5, adjusted p-val ≤ 0.1) in disseminated tumor cells relative to primary tumor cells in the G12 PDX model was performed using STRING. Membership of the phosphoproteins in each pathway is denoted by the same colors as the pathway annotated.

(b-c) G6 and G12 PDX tumor-bearing brains were coronally sectioned and analyzed by co-IF microscopy. Images from separate channels for (b) pT373-RB1 and (c) pS807/811-RB1 from **Figure 6d-g** are shown. Scale bars = 40 µm.

Supplementary Figure 11. Gating strategies

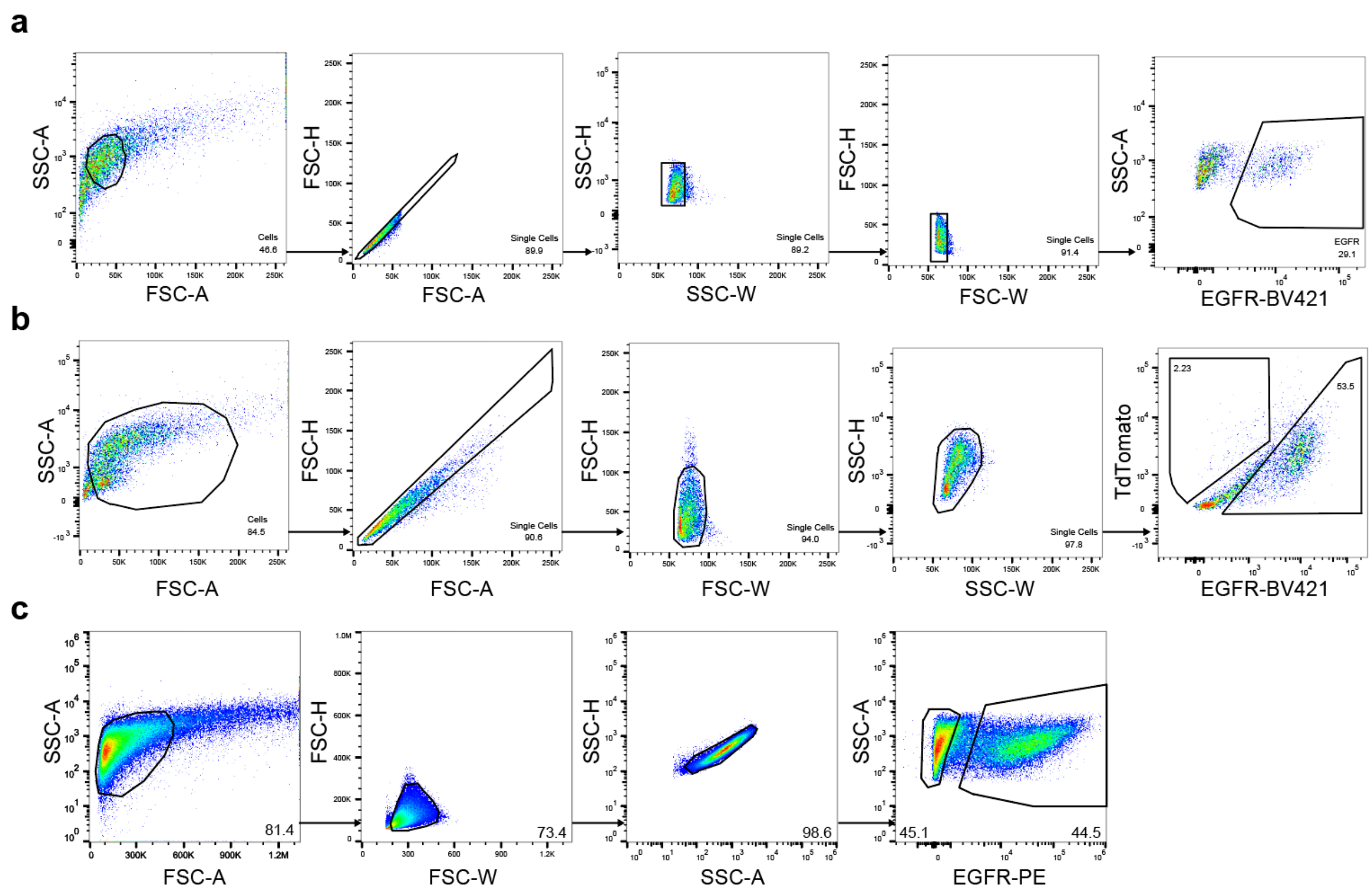

(a) Gating strategy used to evaluate fixation conditions with anti-EGFR-BV421 staining (BioLegend, Cat# 352911; 1:200).
(b) Gating strategy for mixed normal and TdTomato-positive tumor cell populations stained with either anti-HLA-A/B/C-APC-Cy7 [W6/32] (BioLegend, Cat# 311426; 1:2000) or anti-EGFR-BV421 (BioLegend, Cat# 352911; 1:100–1:200).
(c) Gating strategy for dissociated patient-derived xenograft tumor-bearing brains. Cells were first gated by FSC-A versus SSC-A to exclude debris and aggregates, followed by FSC-W versus FSC-H and SSC-H versus SSC-A to isolate singlets. EGFR-positive and EGFR-negative populations were then separated using EGFR-PE fluorescence versus SSC-A.
