# Supplementary material for: Uncovering the signaling networks of disseminated glioblastoma cells in vivo with INSIGHT
Source: Nat Commun. 2026 Aug 8;17:9536. doi: 10.1038/s41467-026-76587-0 (PMC13547291; doi:10.1038/s41467-026-76587-0)
Supplement: Supplementary file 4 [file 41467_2026_76587_MOESM2_ESM.pdf]

Descriptions of Additional Supplementary Files

Supplementary Data 1: Characteristics of the Mayo Clinic glioblastoma patient-derived xenograft (PDX) models G6 and G12. The table summarizes patient demographics, tumor pathology, molecular features, genomic alterations, treatment history, survival, and xenograft characteristics. Data were obtained from the Mayo Clinic Brain Tumor Patient-Derived Xenograft National Resource.

Supplementary Data 2: Differentially abundant proteins and phosphosites in primary and disseminated G6 and G12 PDXs. The table reports differential expression set assignment, log2 fold change (logFC), nominal P value, adjusted P value, gene symbols, protein names, phosphorylated residue(s), UniProt accession(s), and whether the phosphosite was identified as altered by tissue fixation.

Supplementary Data 3: Re-analyzed RNAseq result of G6 and G12. The table contains Ensembl gene identifiers, gene symbols, species, and normalized expression values for the G6 and G12 samples.

Supplementary Data 4: Kinases whose substrates are predicted by Kinase Library to be depleted under the conditions tested in Extended Data Figure 1a. For each pathway, the table reports the log2 enrichment score (enrichment_value_log2), nominal P value (p_value), Benjamini-Hochberg-adjusted P value (adjusted_p_value), absolute -log10-transformed nominal P value (p_value_log10_abs), absolute -log10-transformed adjusted P value (adjusted_p_value_log10_abs)

Supplementary Data 5: Kinases whose substrates are inferred by Kinase Library to be depleted after the dissociation of mouse brain tissue using INSIGHT. The table reports kinase name and group, the number of hits and set sizes for upregulated, downregulated, and background phosphosite sets, enrichment statistics (enrichment value, log2 enrichment value, nominal P value, adjusted P value, and corresponding -log10-transformed values) for upregulated and downregulated phosphosites, and the dominant direction of enrichment with its associated statistics.

Supplementary Data 6: Distribution and the overlap of quantified proteins and phosphopeptides between G6 and G12 models, both globally and of ion channels and neurotransmitter receptors. The table summarizes which proteins and phosphopeptides were detected in both G6 and G12, and indicates whether each was significantly regulated in disseminated cells in both models or in only one of the two and whether they are categorized as neurotransmitter receptors or neuronal structures or ion channels

Supplementary Data 7: List of all phosphorylated kinases quantified in G6 and G12 primary and disseminated tumor cells, annotated according to reported activating or inhibitory regulatory effects.
